# Discovery of ZLC491 as a Potent, Selective, and Orally Bioavailable CDK12/13 PROTAC Degrader

**DOI:** 10.1021/acs.jmedchem.4c01596

**Published:** 2024-10-10

**Authors:** Licheng Zhou, Kaijie Zhou, Yu Chang, Jianzhang Yang, Bohai Fan, Yuhan Su, Zilu Li, Rahul Mannan, Somnath Mahapatra, Ming Ding, Fengtao Zhou, Weixue Huang, Xiaomei Ren, Jian Xu, George Xiaoju Wang, Jinwei Zhang, Zhen Wang, Arul M. Chinnaiyan, Ke Ding

**Affiliations:** † International Cooperative Laboratory of Traditional Chinese Medicine Modernization and Innovative Drug Discovery of Chinese Ministry of Education (MOE), Guangzhou City Key Laboratory of Precision Chemical Drug Development, College of Pharmacy, Jinan University, 855 Xingye Avenue East, Guangzhou 511400, China; ‡ State Key Laboratory of Chemical Biology, Shanghai Institute of Organic Chemistry, Chinese Academy of Sciences, #345 Lingling Rd., Shanghai 200032, China; § University of Chinese Academy of Sciences, No. 1 Yanxihu Road, Huairou District, Beijing 101408, China; ∥ Michigan Center for Translational Pathology, University of Michigan, Ann Arbor, Michigan 48109, United States; ⊥ Department of Pathology, University of Michigan, Ann Arbor, Michigan 48109, United States; # School of Life Science and Technology, China Pharmaceutical University, 639 Longmian Avenue, Nanjing 211198, China; ∇ Livzon Research Institute, Livzon Pharmaceutical Group Inc., No. 38, Chuangye North Road, Jinwan District, Zhuhai 519000, China; ○ Rogel Cancer Center, University of Michigan, Ann Arbor, Michigan 48109, United States; ◆ Howard Hughes Medical Institute, University of Michigan, Ann Arbor, Michigan 48109, United States; ¶ Department of Urology, University of Michigan, Ann Arbor, Michigan 48109, United States

## Abstract

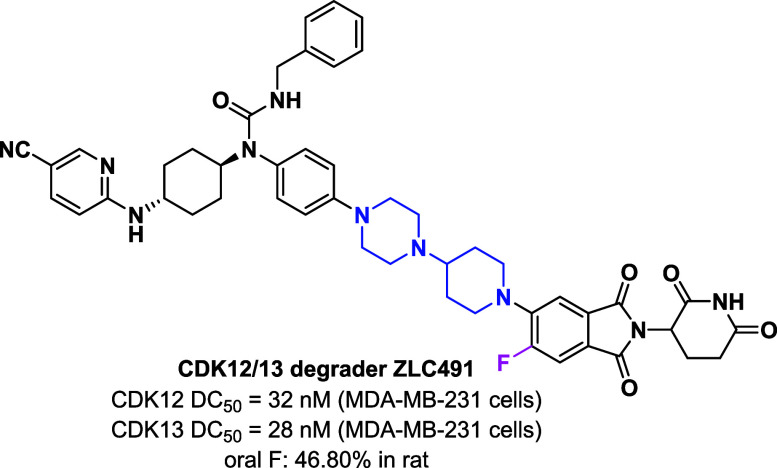

Selective degradation of cyclin-dependent kinases 12 and 13 (CDK12/13) emerges as a new potential therapeutic approach for triple-negative breast cancer (TNBC) and other human cancers. While several proteolysis-targeting chimera (PROTAC) degraders of CDK12/13 were reported, none are orally bioavailable. Here, we report the discovery of **ZLC491** as a potent, selective, and orally bioavailable CDK12/13 PROTAC degrader. The compound effectively degraded CDK12 and CDK13 with DC_50_ values of 32 and 28 nM, respectively, in TNBC MDA-MB-231 cells. Global proteomic assessment and mechanistic studies revealed that **ZLC491** selectively induced CDK12/13 degradation in a cereblon- and proteasome-dependent manner. Furthermore, the molecule efficiently suppressed transcription and expression of long genes, predominantly a subset of genes associated with DNA damage response, and significantly inhibited proliferation of multiple TNBC cell lines. Importantly, **ZLC491** achieved an oral bioavailability of 46.8% in rats and demonstrated potent *in vivo* degradative effects on CDK12/13 in an MDA-MB-231 xenografted mouse model.

## Introduction

Cyclin-dependent kinases (CDKs) are evolutionarily conserved serine/threonine protein kinases that regulate cell cycle progression and gene transcription by interacting with their corresponding cyclins.^1^ CDK12 and its paralog CDK13 are transcription-associated CDKs with cyclin K (CCNK) as the partner protein. Upon complex formation with CCNK, CDK12/13 phosphorylate the carboxy-terminal domain of RNA polymerase II to orchestrate transcription elongation and termination.^2−4^ CDK12/13 primarily regulate the transcription of DNA-damage response (DDR) genes to maintain genomic stability. Increasing evidence suggests the therapeutic potential of targeting CDK12/13 in a variety of human cancers,^5−8^ including triple-negative breast cancer (TNBC), which has limited treatment options.^9−13^ For example, CDK12 depletion by siRNA inhibited the migration and invasion of TNBC MDA-MB-231 cells, and this effect was reversed by reintroducing CDK12 with *CDK12* cDNA.^14^ Double knockdown of CDK12 and CDK13 by Clustered Regularly Interspaced Short Palindromic Repeats (CRISPR)-Cas9 led to reduced DDR gene expression and suppressed colony formation of MDA-MB-231 cells.^10^ Notably, pharmacological inhibition of CDK12 and CDK13 by small-molecule inhibitors demonstrated potent antitumor efficacy alone or in combination with cisplatin in an orthotopic TNBC patient-derived xenograft (PDX) model.^10^

**Figure 1:**
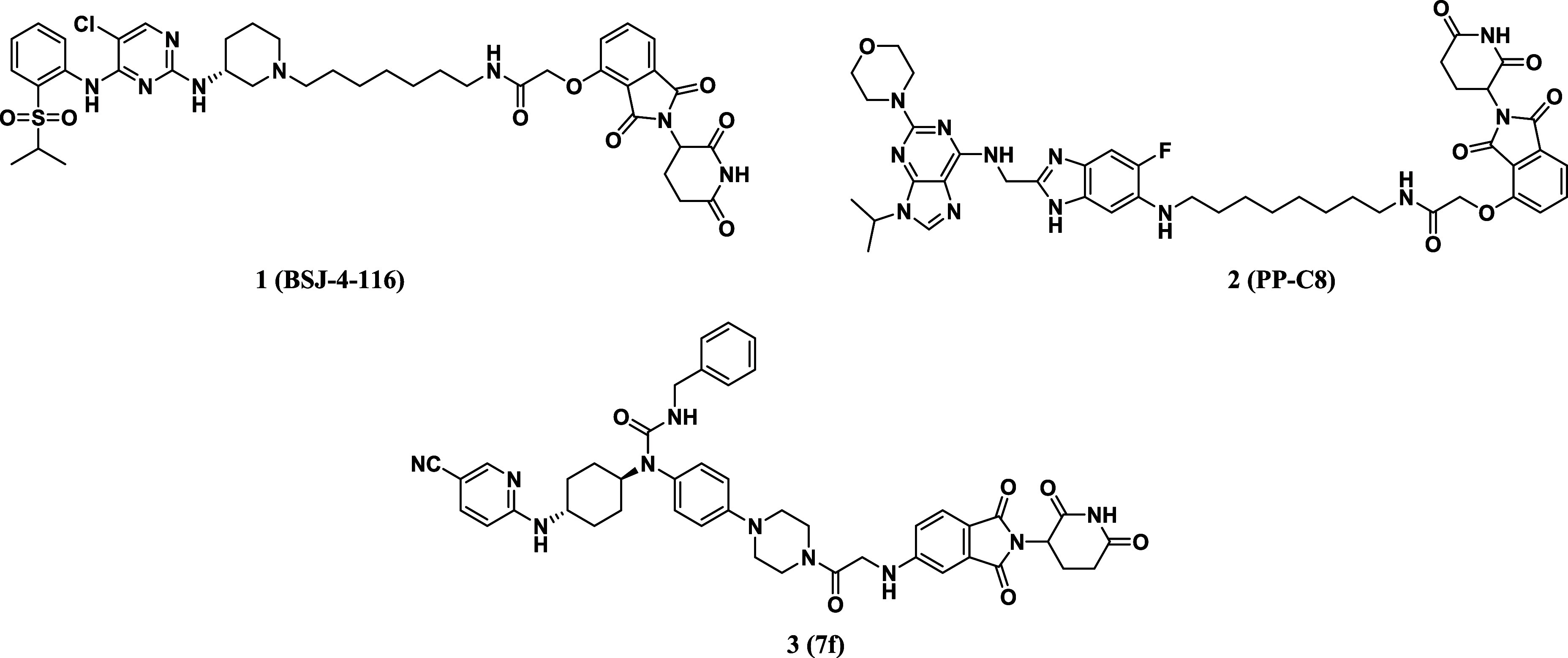
Chemical structures of previously reported CDK12/CDK13 PROTACs.

Despite progress in developing CDK12/13 kinase inhibitors,^10,15−18^ none have reached clinical trials. Therefore, discovering new CDK12/13 modulators is highly desirable. Recently, several Proteolysis TArgeting Chimeras (PROTACs) degraders for CDK12/13 were reported, including BSJ-4-116 (**1**),^19^ PP-C8 (**2**)^20^ and 7f (**3**)^21^ (Figure 1). Compound **1**, the first selective CDK12 degrader, was reported by Gray and coworkers in 2021. This compound demonstrated potent antiproliferative activity in T-cell acute lymphoblastic leukemia cells and showed a synergistic effect with poly(ADP-ribose) polymerase (PARP) inhibitor olaparib. Compound **2**, another selective CDK12 PROTAC degrader disclosed by Zhu and coworkers in 2022, also synergized with PARP inhibitor in TNBC cells. Our group reported the discovery of compound **3** in 2022 as a potent and selective dual PROTAC degrader of CDK12/13. Compound **3** effectively degraded CDK12 and CDK13 and significantly inhibited the proliferation of multiple TNBC cell lines. However, these compounds were shown to degrade multiple other proteins in addition to CDK12/13 in the global proteomic assays, and none demonstrated acceptable oral bioavailability. Here, we report the discovery of **ZLC491**, a new highly selective and orally bioavailable CDK12/13 PROTAC degrader.

## Results and Discussion

### Molecular Design

**Figure 2:**
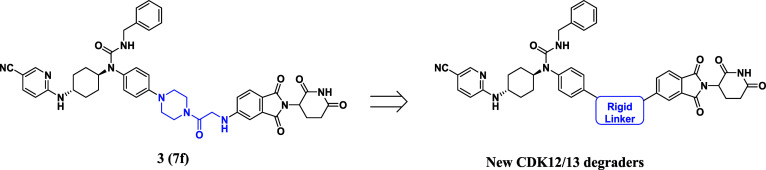
Design of new CDK12/13 PROTACs with rigid linkers.

We previously reported the discovery of compound **3** as the first dual PROTAC degrader of CDK12 and CDK13.^21^ Compound **3** potently degraded CDK12 and CDK13 with DC_50_ values of 2.2 and 2.1 nM, respectively, in MDA-MB-231 cells, however, it also significantly decreased the levels of several other proteins in global proteomic studies. Additionally, this compound demonstrated poor pharmacokinetic (PK) properties, with nondetectable oral bioavailability and high clearance values in rats.^21^ Studies have suggested that introducing a conformationally rigid linker in a PROTAC degrader could improve degradation potency, selectivity, and PK properties.^22,23^ Therefore, a series of new CDK12/13 degraders were designed and synthesized by introducing rigid linkers in the lead compound **3** (Figure 2), aiming to improve the target specificity and PK profiles. The degradation efficiency of the new molecules was assessed by immunoblotting after treatment with the compounds at 0.3 μM in MD-MBA-231 cells for 15 h.

### Chemical Synthesis

**Scheme 1:**
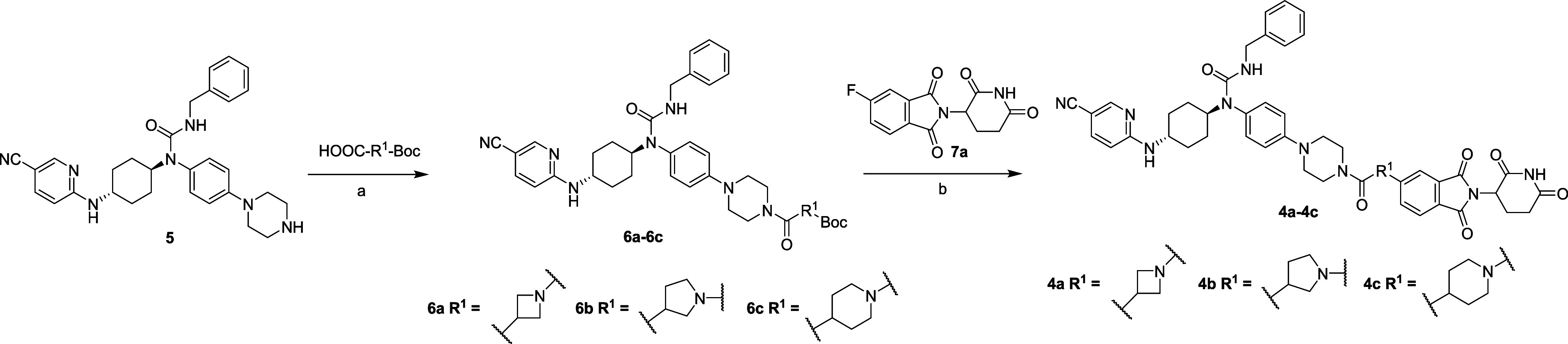
Synthesis of Compounds **4a**–**c** Reagents and conditions: (a) 2-(7-azabenzotriazol-1-yl)-*N*′,*N*′,*N*′-tetramethyluronium hexafluorophosphate (HATU), *N*,*N*-diisopropylethylamine (DIPEA), *N*,*N*-dimethylformamide (DMF), room temperature (rt), 2 h; (b) (i) trifluoroacetic acid (TFA), dichloromethane (CH_2_Cl_2_), rt, 1 h; (ii) DIPEA, 100 °C, 2 h.

The synthesis of compounds **4a**–**c** is depicted in Scheme 1. The starting material **5** was synthesized according to the previously reported procedure.^21^ The amide coupling reaction between compound **5** and various carboxylic acids afforded compounds **6a**–**c**. Deprotection of *tert*-butoxycarbonyl group of compounds **6a**–**c** and subsequent nucleophilic substitution with compound **7a** provided compounds **4a**–**c**.

**Scheme 2:**
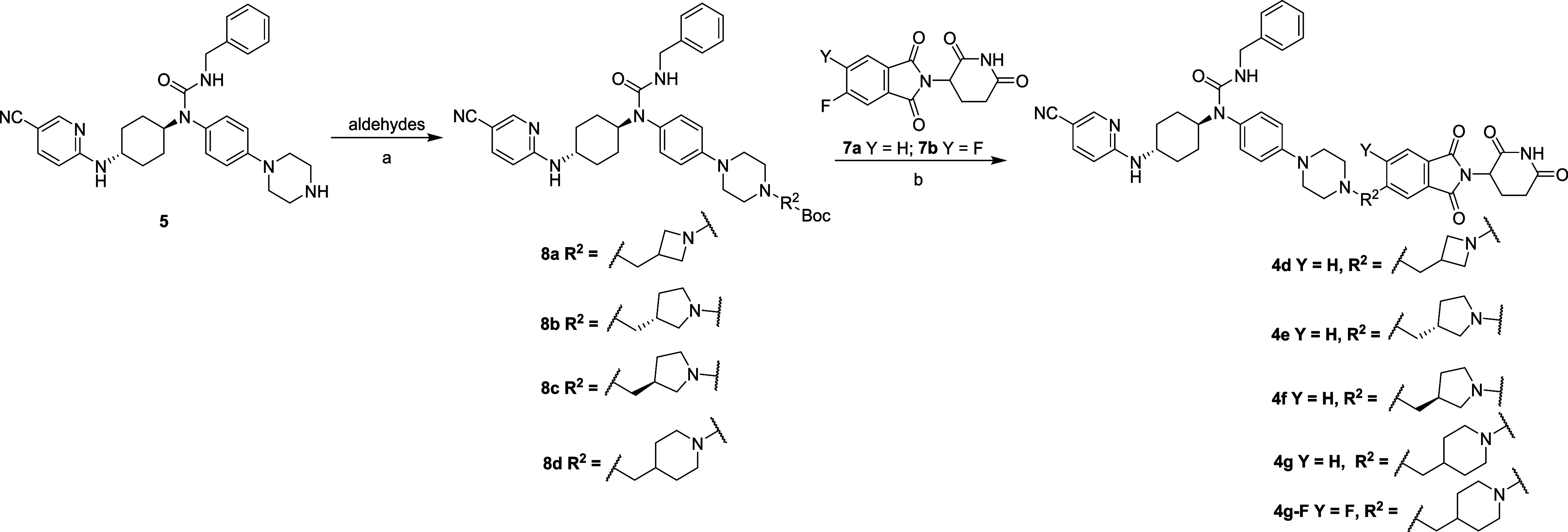
Synthesis of Compounds **4d**–**g** and **4g**–**F** Reagents and conditions: (a) aldehydes, sodium triacetoxyborohydride (NaBH(OAc)_3_), AcOH, CH_2_Cl_2_, rt, 8 h; (b) (i) TFA, CH_2_Cl_2_, rt, 1 h; (ii) DIPEA,100 °C, 2 h.

The synthetic routes of compounds **4d**–**g** and **4g**–**F** are shown in Scheme 2. The reductive amination reaction of compound **5** with various aldehydes yielded intermediates **8a**–**d**. Deprotection of *tert*-butoxycarbonyl group of compounds **8a**–**d** and subsequent nucleophilic substitution with compound **7a** or **7b** provided compounds **4d**–**g** and **4g**–**F**.

**Scheme 3:**
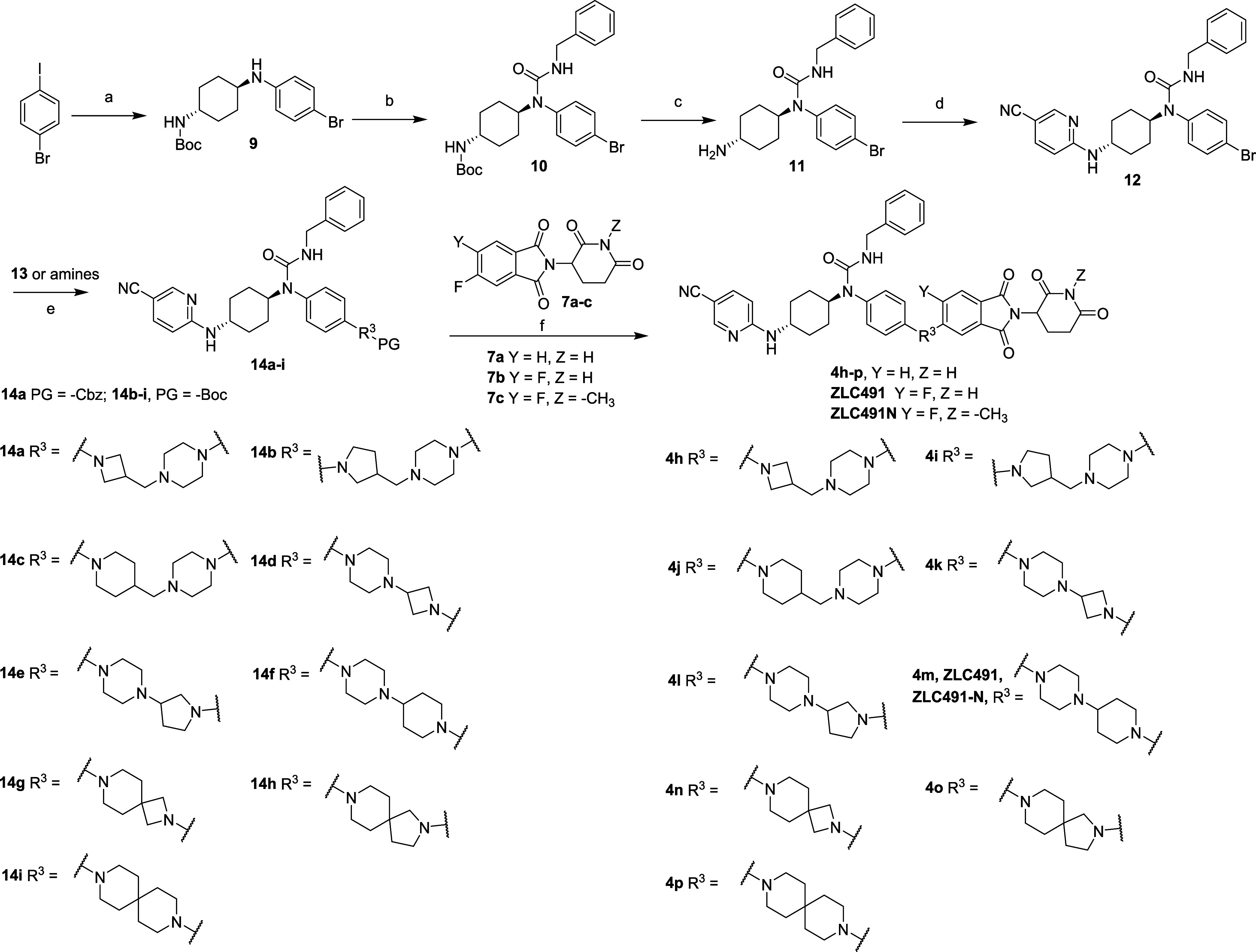
Synthesis of Compounds **4h**–**p**, **ZLC491** and **ZLC491N** Reagents and conditions: (a) *trans*-*N*-Boc-1,4-cyclohexanidiamine, tris(dibenzylideneacetone) dipalladium (Pd_2_(dba)_3_), 4,5-bis(diphenylphosphino)-9,9-dimethylxanthene (Xantphos), sodium *tert*-butoxide (*tert*-BuONa), toluene, 100 °C, overnight; (b) benzyl isocyanate, DIPEA, DMF, 95 °C, overnight; (c) TFA, CH_2_Cl_2_, rt, 1 h; (d) 5-cyano-2-fluoropyridine, cesium carbonate (Cs_2_CO_3_), DMF, rt, overnight; (e) **14a**: intermediate **13**, Pd_2_(dba)_3_, Xantphos, *tert*-BuONa, toluene, 110 °C, 4 h; **14b**–**i**: amines, Pd_2_(dba)_3_, Xantphos, *tert*-BuONa, toluene, 110 °C, overnight; (f) **4h**: (i) Pd/C, H_2_, rt; (ii) DIPEA, 100 °C, 2 h; **4i**–**p**, **ZLC491** and **ZLC491N**: (i) TFA, CH_2_Cl_2_, rt, 1 h; (ii) DIPEA, 100 °C, 2 h.

Compounds **4h**–**p**, **ZLC491** and **ZLC491N** were synthesized according to the methods in Scheme 3. Compound **9** was prepared by a coupling reaction between 1-bromo-4-iodobenzene and *trans*-*N*-Boc-1,4-cyclohexanidiamine. The nucleophilic substitution reaction of compound **9** with benzyl isocyanate and subsequent deprotection by trifluoroacetic acid provided compound **11**, which further reacted with 5-cyano-2-fluoropyridine to offer intermediate **12**. Compounds **14a**–**i** were synthesized by the Buchwald–Hartwig amination reaction between compound **12** and intermediate **13** or amines. Compounds **4h**–**p**, **ZLC491** and **ZLC491N** were obtained by the deprotection reaction and a following substitution reaction.

### Discovery of New CDK12/13 Degrader ZLC491

Compounds **4a**–**c** were initially designed and synthesized by cyclizing the secondary amine of the linker in compound **3**. The resulting compounds exhibited decreased degrading potencies compared to the lead molecule **3** (Table 1). For instance, compound **4b** degraded CDK12 and CDK13 by 61% and 65%, respectively, at 0.3 μM in MDA-MB-231 cells, while the corresponding values of compound **3** were 75% and 84% under the same conditions. To decrease the hydrophobicity, compounds **4d**–**g**, where the neutral carboxylamide linker in **4a**–**c** was replaced by a hydrophilic alkaline moiety, were designed and synthesized. The ethylene-linked molecules (**4d**–**g**) generally exhibited increased degradative potency compared to their carboxylamide linker counterparts (**4a**–**c**). Compound **4g** showed degradative ratios of 74% and 84% against CDK12 and CDK13, respectively.

**Table 1:**
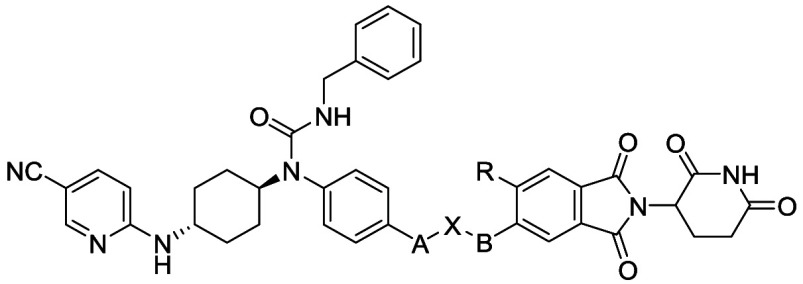

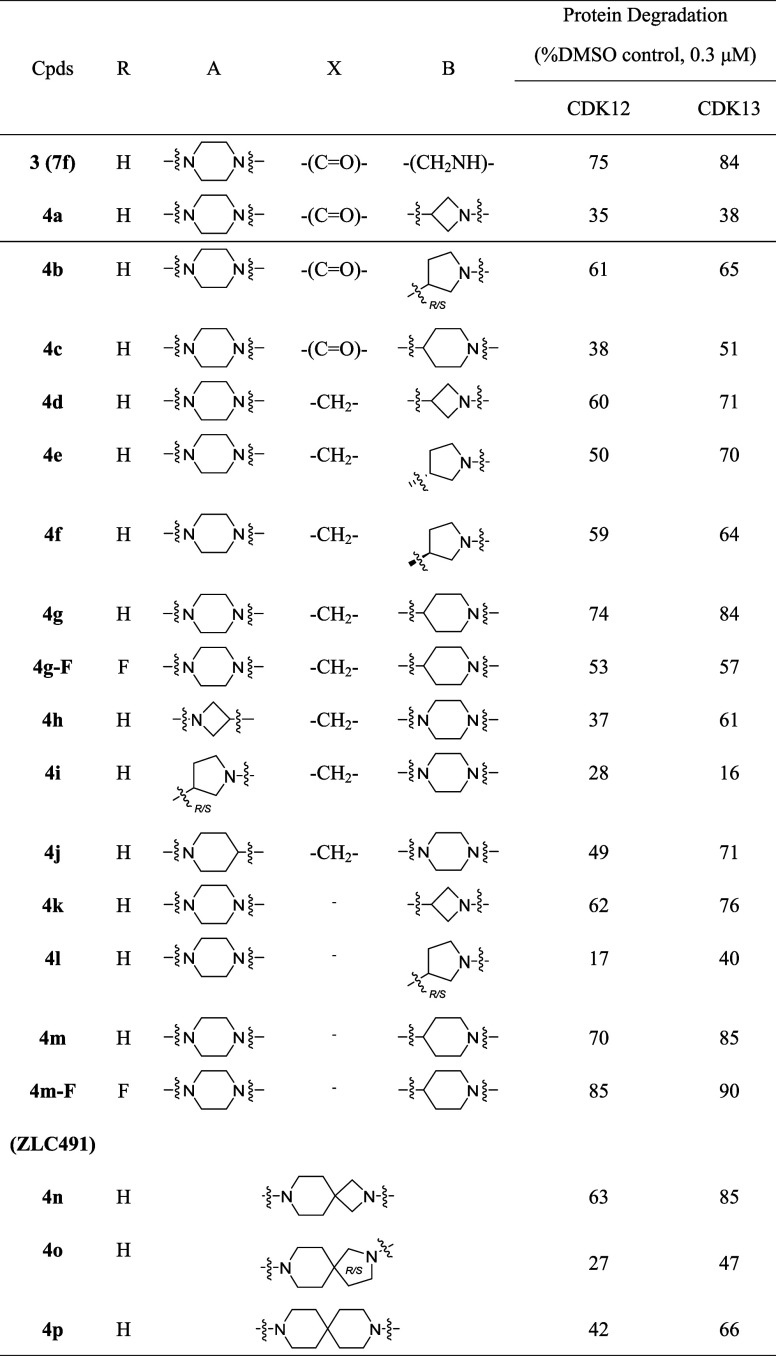
Degradation Efficiency of Compounds **4a**–**p**, **4g-F** and **ZLC491**^a^

a MDA-MB-231 cells were treated with compounds at 0.3 μM for 15 h. CDK12/13 protein levels were determined by immunoblotting and normalized against α-tubulin. Data represent the geometric mean of three replicates.

It is well recognized that introducing a fluorine (F) atom can potentially improve the PK properties of the molecule. For example, the PROTAC degrader ARV-110, which bears a fluorine-substituted thalidomide, achieved good oral bioavailability.^24,25^ Therefore, we designed and synthesized compound **4g**–**F** by introducing F into compound **4g**. However, compound **4g**–**F** degraded CDK12 and CDK13 by 53% and 57%, respectively, and was less active than compound **4g**.

To investigate the optimal combination style, compounds **4h**–**j** were constructed by exchanging the nitrogen-containing aliphatic rings on both sides of the ethylene moiety in compounds **4d**–**g**. These compounds (**4h**–**j**) were generally less potent than **4d**–**g** for CDK12/13 degradation. To further increase the rigidity of the linker, we designed and synthesized compounds **4k**–**m** by directly connecting two nitrogen-containing aliphatic rings. The most potent compound (**4m**) displayed degradative rates of 70% and 85% for CDK12 and CDK13, respectively (Table 1).

Next, we designed and synthesized compound **4m-F** (**ZLC491**) by introducing F into compound **4m**. This compound degraded CDK12 and CDK13 by 85% and 90%, respectively, and was more active than compound **4m.** Additionally, spiro-cyclically linking molecules (**4n**–**p)** with increased conformational rigidity were designed and synthesized. While the most potent spiro-cyclical linking compound (**4n**) was found to degrade CDK12 and CDK13 by 63% and 85%, respectively, at 0.3 μM (Table 1), it was less potent than **4m-F**.

### ZLC491 Induced Degradation of CDK12/13 in a Concentration- and Time-Dependent Manner

**Figure 3:**
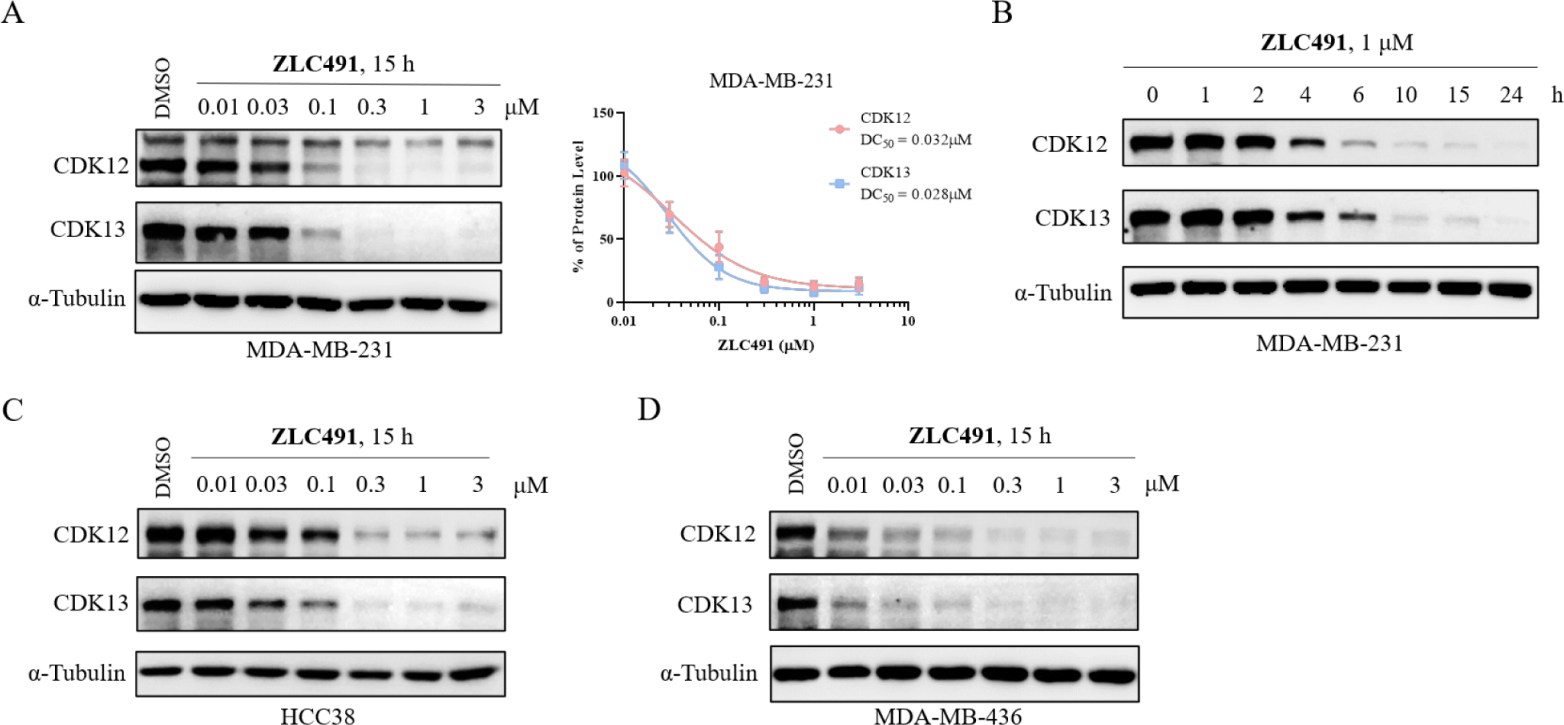
Compound **ZLC491** effectively reduced CDK12/13 protein level in a concentration- and time-dependent fashion. (A) Immunoblotting of CDK12 and CDK13 in MDA-MB-231 cells treated with increasing concentrations of **ZLC491** for 15 h (left). Percent remaining CDK12 and CDK13 proteins were plotted for DC_50_ determination (right). Protein levels were quantified using ImageJ and normalized to corresponding α-tubulin, then plotted using Graphpad Prism 8.0. The data were representative of three independent experiments; (B) immunoblotting of CDK12 and CDK13 proteins in MDA-MB-231 cells treated with **ZLC491** for various time points; immunoblotting of CDK12 and CDK13 in HCC38 (C) and MDA-MB-436 (D) cells treated with increasing concentrations of **ZLC491** for 15 h. α-Tubulin was used as a loading control.

Compound **ZLC491** showed the best degradation rates for CDK12/13 in preliminary screening. To further characterize the compound, we determined the half-degradation concentration (DC_50_) values of **ZLC491** in MDA-MB-231 cells. **ZLC491** dose-dependently degraded CDK12 and CDK13 with DC_50_ values of 32 nM and 28 nM, respectively (Figures 3A and S5A). We also treated MDA-MB-231 cells with 1 μM **ZLC491** at various time points. The results revealed that **ZLC491** effectively degraded CDK12/13 in a time-dependent manner, with a significant reduction of CDK12/13 observed as early as 4 h (Figure 3B). Additionally, **ZLC491** was found to deplete CDK12/13 in a concentration-dependent manner in other TNBC cells including HCC38 and MDA-MB-436 cells (Figure 3C,D). These results collectively suggested that **ZLC491** induced CDK12/13 degradation in a concentration- and time-dependent fashion.

### ZLC491 Induced CDK12/13 Degradation in a Cereblon (CRBN)- and Proteasome-Dependent Manner

**Figure 4:**
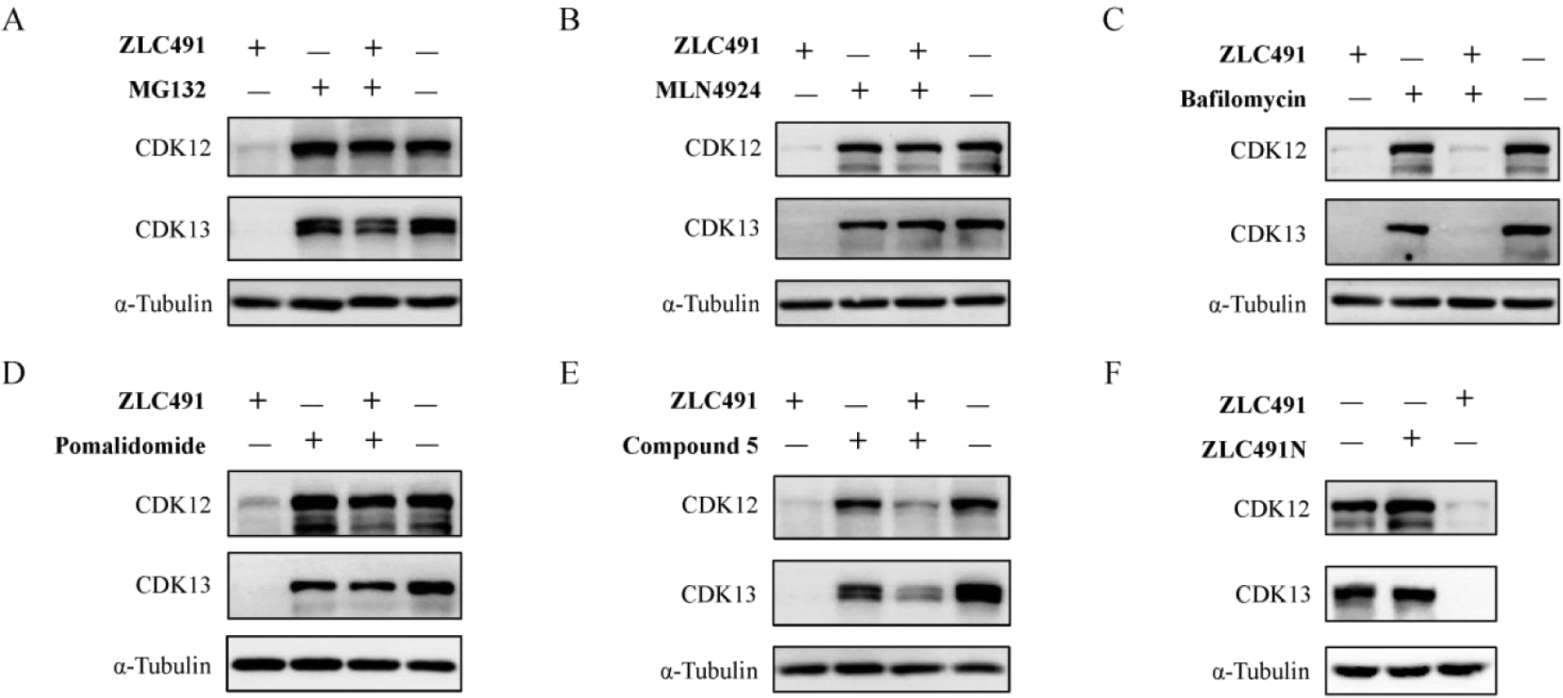
**ZLC491** induced CDK12/13 degradation in a CRBN- and proteasome-dependent fashion. Immunoblotting of CDK12 and CDK13 in MDA-MB-231 cells treated with 1 μM **ZLC491** for 10 h with or without pretreatment with 1 μM MG132 (A), 0.1 μM MLN4924 (B), 0.1 μM bafilomycin (C), 5 μM pomalidomide (D) and 8 μM compound **5** (E) for 2 h; (F) immunoblotting of CDK12 and CDK13 in MDA-MB-231 cells treated with 1 μM **ZLC491N** for 10 h. α-Tubulin was used as a loading control.

We further investigated the mechanism of action of **ZLC491**-mediated CDK12/13 degradation. The results revealed that pretreatment of MDA-MB-231 cells with the proteasome inhibitor MG132 or the NEDD8-activating enzyme (NAE) inhibitor MLN4924, but not the lysosomal inhibitor bafilomycin, significantly blocked the **ZLC491**-induced CDK12/13 degradation (Figure 4A–C). Additionally, pretreatment with CRBN ligand pomalidomide or the warhead compound **5** also reversed the **ZLC491**-mediated CDK12/13 degradation in MDA-MB-231 cells (Figure 4D,E). We also designed and synthesized the negative control compound **ZLC491N** (Scheme 3) by introducing a methyl group on thalidomide moiety of **ZLC491**, which showed no degradative effect on CDK12/13 (Figure 4F). These results suggested that **ZLC491** induced CDK12/13 degradation in a CRBN- and proteasome-dependent manner.

### ZLC491 Degraded CDK12/13 with High Selectivity

**Figure 5:**
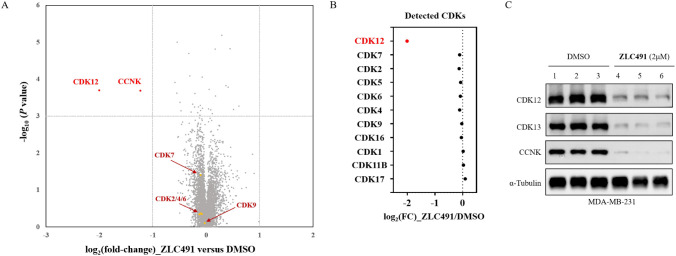
**ZLC491** degraded CDK12/13 with high selectivity. (A) Global proteomic analysis of **ZLC491** in MDA-MB-231 cells after 8 h treatment of DMSO or 2 μM **ZLC491**; (B) mass-spec quantification of CDKs changes; (C) immunoblotting of target protein degradation in samples for proteomic analysis.

The global proteomic selectivity of **ZLC491** was investigated by using the tandem mass tag (TMT) labeled mass-spectrometry. The results indicated that **ZLC491** was a very specific CDK12/13 degrader, with only CDK12 and its partner protein CCNK significantly decreased in MDA-MB-231 cells (Figure 5A). Notably, **ZLC491** was selective for CDK12 over other CDKs (Figure 5A,B). CDK13 was not detected in either degrader treatment groups or DMSO control groups, likely due to its insufficient cellular expression for mass spectrometry detection. Therefore, we conducted an immunoblotting analysis using the same samples and found that CDK13 was significantly degraded to a degree similar to CDK12 and CCNK (Figure 5C). We also confirmed **ZLC491** dose-dependently degraded CCNK in MDA-MB-231 cells by Western blotting (Figure S3).

### ZLC491 Effectively Reduced DDR Gene Expression and Potently Inhibited TNBC Cell Growth Alone or in Combination with DNA Damaging Agent or PARP Inhibitor

**Figure 6:**
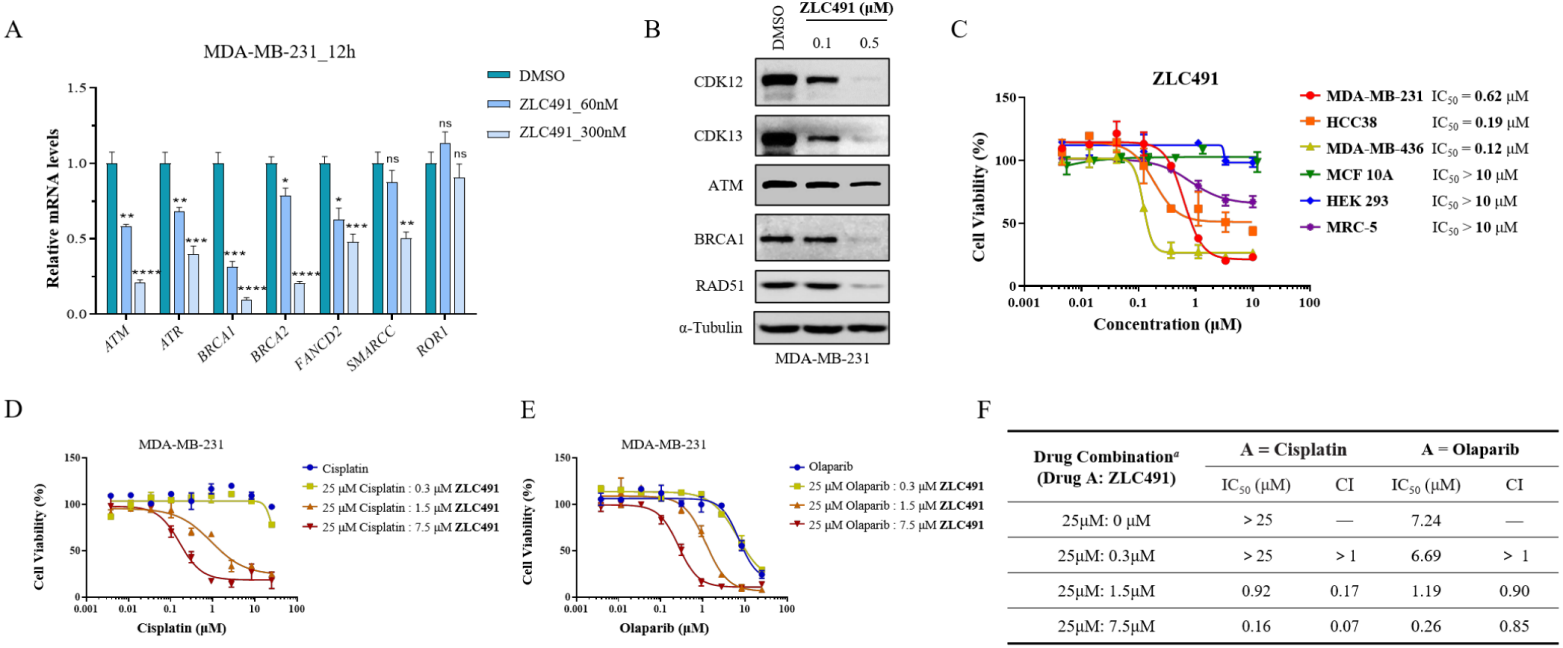
**ZLC491** effectively reduced DDR gene expression and potently inhibited TNBC cell growth alone or combined with DNA damaging agents or PARP inhibitors. (A) Transcription level of DDR genes in MDA-MB-231 cells determined by RT-qPCR upon the treatment with vehicle or **ZLC491** at different doses for 12 h, *ROR*1 was used as a negative control; (B) immunoblotting of DDR proteins in MDA-MB-231 cells after the treatment of **ZLC491** at different doses for 48 h; (C) cell antiproliferative activity of **ZLC491** in multiple TNBC cells as well as several noncancerous cells. Cells were treated with the compound for 5 days; (D,E) antiproliferative potency of cisplatin or olaparib alone or combined with **ZLC491** at different doses in MDA-MB-231 cells for 5 days. Data are plotted using the percentage of absorbance relative to DMSO controls. (F) IC_50_ values and combination index (CI) in drug combination assay. In this model, CI scores estimate the interaction between the two drugs. If CI < 1, the drugs have a synergistic effect and if CI > 1, the drugs have an antagonistic effect. CI = 1 means the drugs have an additive effect. ^a^The drug combination in the table refers to the starting concentration of the two combined drugs, that would both go through serial dilution when treating cells.

We next evaluated the effect of **ZLC491** on the expression of DDR genes regulated by CDK12/13. As shown in Figure 6A, real-time quantitative polymerase chain reaction (RT-qPCR) assays revealed that **ZLC491** concentration-dependently suppressed the transcription of several DDR genes, including ataxia telangiectasia mutated (*ATM*), ataxia telangiectasia and Rad3 related (*ATR*), breast cancer susceptibility gene type 1 and 2 (*BRCA*1, *BRCA*2), Fanconi anemia complementation group D2 (*FANCD*2) and SWI/SNF related, matrix associated, actin dependent regulator of chromatin, subfamily C (*SMARCC*). Further, the protein levels of DDR-relative proteins, including DNA homologous recombination repair protein BRCA1 and RAD51, and DNA double-strand break/single-strand break repair protein ATM, were also reduced upon treatment with **ZLC491** (Figure 6B). These results suggested that **ZLC491** effectively reduced the expression of DDR genes.

The antiproliferative effects of **ZLC491** against TNBC cells were also evaluated. The results in Figure 6C showed that **ZLC491** potently inhibited the growth of MDA-MB-231, HCC38, and MDA-MB-436 cells with IC_50_ values of 0.62, 0.19 and 0.12 μM, respectively, while it was significantly less active in several noncancerous cell lines including mammary epithelial cells MCF 10A, human embryonic kidney cells HEK 293 and human embryonic lung cells MRC-5 (IC_50_*s* > 10 μM). As expected, the negative control compound **ZLC491N** showed no antiproliferative effects in these cell lines at the indicated concentrations (Figure S4). Previous studies demonstrated that CDK12/13 inhibition or degradation synergized with DNA-damaging therapeutics to impair TNBC cell growth.^26,27^ We thus investigated the synergistic effects of **ZLC491** with DNA cross-linker cisplatin. Dose–response assays revealed potent synergy between **ZLC491** and cisplatin in MDA-MB-231 cells (Figure 6D). A similar synergistic effect was observed for the combination of **ZLC491** and the PARP inhibitor olaparib (Figure 6E). These results suggested that **ZLC491** potently inhibited MDA-MB-231 cell growth alone or in combination with DNA damaging agents or PARP inhibitors (Figure 6F).

### ZLC491 Was Orally Bioavailable in Rats

We further evaluated the *in vivo* PK properties of compound **ZLC491** and the counterpart **4m** (without F substitution) in rats, and the results were summarized in Table 2. It was revealed that both **ZLC491** and **4m** showed significantly improved PK profiles compared to compound **3**. While compound **3** was not orally bioavailable,^21^ compound **4m** displayed a bioavailability of 16% when orally dosed. The PK profiles of F-containing compound **ZLC491** were further improved. It displayed an oral bioavailability of 46.8%, with a maximum plasma concentration (*C*_max_) of 1309.81 ng/mL and an area under the drug concentration-time curve (AUC) of 12759.01 h·ng/mL (Table 2).

**Table 2:** PK Parameters of Compounds **4m** and **ZLC491** in Rats^a^

Compds	Route	*T*_1/2_ (h)	*C*_max_ (ng/mL)	AUC (0 – t) (h·ng/mL)	CL (mL/min/kg)	*F* (%)
**4m**	i.v.2.5 mg/kg	2.38	2593.29	4722.41	9.85	-
	p.o.10 mg/kg	3.04	308.30	3108.90	-	16.46
**ZLC491**	i.v.2.5 mg/kg	2.46	4586.27	6815.36	6.38	-
	p.o.10 mg/kg	2.92	1309.81	12759.01	-	46.80

a Data represent the geometric mean of three replicates.

### ZLC491 Depleted CDK12 and CDK13 Proteins *In Vivo*

**Figure 7:**
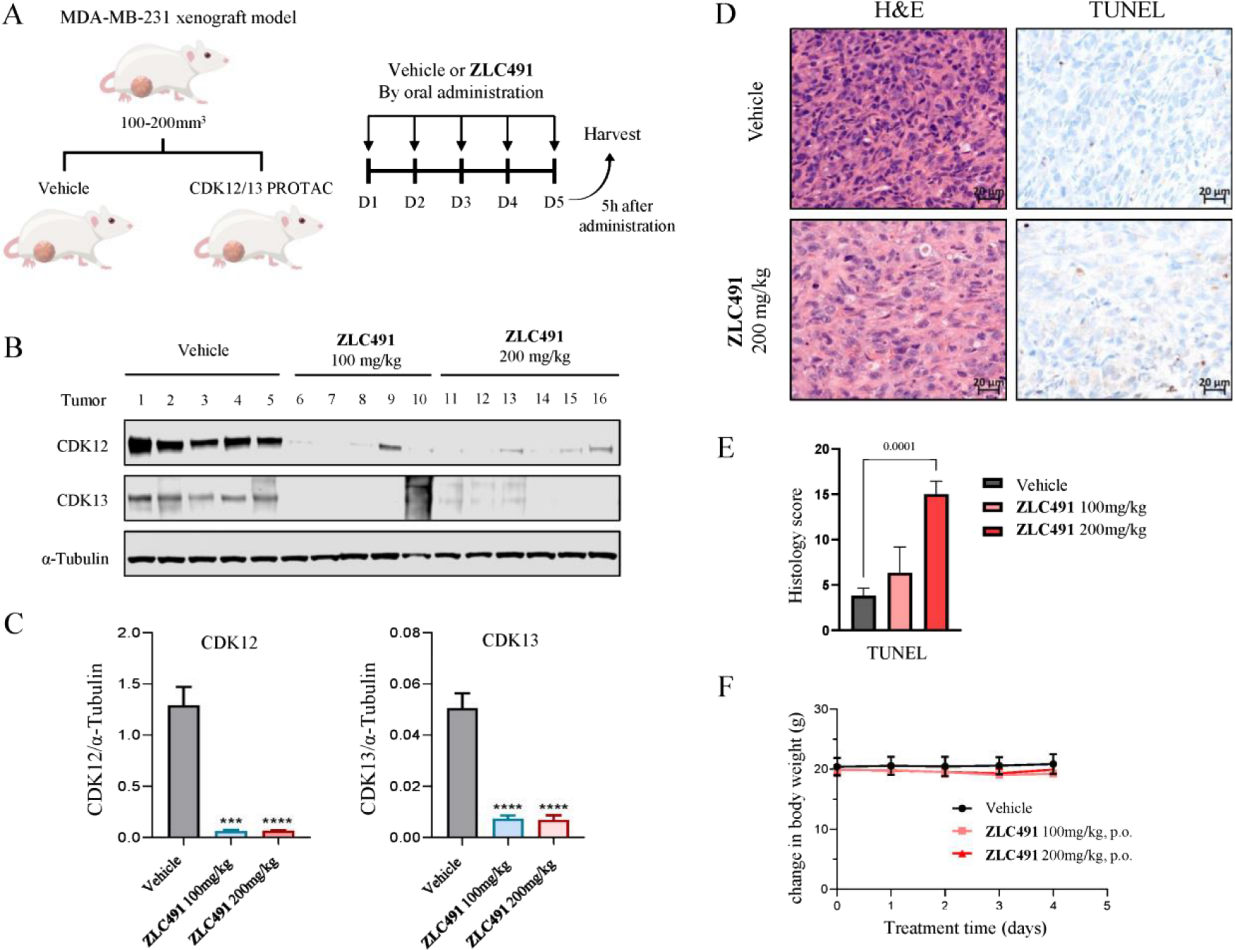
**ZLC491** depleted CDK12 and CDK13 proteins *in vivo*. (A) Study design of pharmacodynamics assessment on target engagement of **ZLC491** in the MDA-MB-231 xenograft model; (B) immunoblotting of CDK12, CDK13, and α-tubulin in tissue lysate of MDA-MB-231 xenografts model after **ZLC491** treatment; (C) protein levels were quantified using ImageJ and normalized to corresponding α-tubulin, then plotted using GraphPad Prism 8.0; (D) immunohistochemistry (IHC) analysis on the tumors by H&E staining and TUNEL assays; (E) TUNEL histology scores were analyzed and plotted by GraphPad Prism 8.0; (F) body weight change during the treatment.

We further performed pharmacodynamic studies of **ZLC491** in an MDA-MB-231 xenografted mouse model. **ZLC491** was orally administrated at two doses of 100 and 200 mg/kg, respectively, for 5 consecutive days (Figure 7A). Tumor tissues were harvested on day 5 and subjected to Western blot analysis. The results showed that compared to the vehicle control group, **ZLC491** significantly depleted CDK12 and CDK13 proteins in tumor tissues at both doses, demonstrating its strong degradation efficiency on CDK12 and CDK13 *in vivo* (Figure 7B, C). Additionally, we conducted further histological analysis on the tumors following **ZLC491** treatment. As shown in Figure 7D,E, marked apoptosis and increased TUNEL signaling were observed in the H&E staining and TUNEL immunohistochemistry (IHC) assays, particularly at the higher dose of 200 mg/kg, suggesting that **ZLC491** induced apoptotic cell death in the tumors. Importantly, **ZLC491** was well tolerated by the treated mice, as indicated by the absence of body weight loss during the treatment (Figure 7F).

## Conclusions

In summary, we report the discovery of **ZLC491** as a potent, highly selective, and orally bioavailable CDK12/13 PROTAC degrader. **ZLC491** efficiently degraded CDK12 and CDK13 with DC_50_ values of 32 and 28 nM, respectively, in TNBC MDA-MB-231 cells. Global proteomics assessment demonstrated that **ZLC491** selectively targeted CDK12/13. Mechanistic studies revealed that **ZLC491** induced CDK12/13 degradation in a cereblon- and proteasome-dependent manner. **ZLC491** effectively suppressed the expression of downstream DNA damage response genes and potently inhibited the proliferation of multiple TNBC cell lines alone or in combination with DNA damaging agents or PARP inhibitors. Importantly, **ZLC491** achieved an oral bioavailability of 46.8% and demonstrated potent degradative effects on CDK12/13 in the MDA-MB-231 xenografted mouse model. This study provided a potent, highly selective, and orally bioavailable CDK12/13 PROTAC degrader for further development as a targeted therapeutic agent for TNBC.

## Experimental Section

### General Methods for Chemistry

All reagents and solvents in chemical synthesis were purchased from commercial vendors and used without further purification, unless indicated otherwise. All reactions were monitored by thin-layer chromatography (TLC) and visualized by UV light visualization (254 and 365 nm). All compounds were purified by a column chromatography on silica gel (200–300 mesh). The ^1^H, ^19^F and ^13^C NMR spectra were recorded on Agilent DD2 500 spectrometer (Agilent Technologies Inc., USA) or Bruker AVANCE 600 spectrometer (Bruker Company, Germany) and referenced with respect to appropriate internal standards or residual solvent peaks (CDCl_3_ = 7.26 ppm, DMSO-*d*_6_ = 2.50 ppm). The following abbreviations were used in reporting spectra, s (singlet), d (doublet), t (triplet), q (quartet), m (multiplet). The spectra of high-resolution mass (HRMS) were monitored by Bruker MaXis 4G TOF Mass Spectrometer and an ESI source. The purities of all final compounds were monitored by HPLC analysis with the Agilent 1200 system. HPLC condition: Triart C18 reversed-phase column, 5 μm, 4.6 mm × 250 mm, and flow rate 1.0 mL/min, starting with a 15 min gradient from 0.1% TFA in water and acetonitrile 9:1 mixture to 0.1% TFA in acetonitrile, then ending with 0.1% TFA in acetonitrile for 5 min. The purity of all final compounds was confirmed to be >95% by HPLC analysis with the Agilent 1260 system.

#### 3-Benzyl-1-((1*r*,4*r*)-4-((5-cyanopyridin-2-yl)amino)cyclohexyl)-1-(4-(piperazin-1-yl)phenyl)urea (**5**)

The synthetic procedure for compound **5** has been described in our previous study.^21 1^ H NMR (500 MHz, DMSO-*d*_6_) δ 8.29 (s, 1H), 7.60 (d, *J* = 9.2 Hz, 1H), 7.48 (s, 1H), 7.28–7.25 (m, 2H), 7.18–7.15 (m, 3H), 7.00–6.95 (m, 4H), 6.47 (d, *J* = 8.9 Hz, 1H), 5.56 (s, 1H), 4.26 (m, 1H), 4.14 (d, *J* = 5.9 Hz, 2H), 3.48 (s, 1H), 3.37–3.34 (m, 1H),3.11–3.09 (m, 4H), 2.85–2.83 (m, 4H), 1.90 (d, *J* = 11.4 Hz, 2H), 1.76 (d, *J* = 10.7 Hz, 2H), 1.33–1.27 (m, 2H), 1.13–1.06 (m, 2H).

### General Procedure for the Synthesis of **6a**–c

To a stirred solution of compound **5** (153 mg, 0.3 mmol) in anhydrous DMF (2 mL) were added HATU (137 mg, 0.36 mmol), DIPEA (0.1 mL, 0.6 mmol) and 1-*N*-Boc-3-azetidinecarboxylic acid (67 mg, 0.33 mmol). The mixture was stirred for 2 h at room temperature until completed as indicated by TLC. After quenching with water, the resulting mixture was filtered. The filter residue was solved with dichloromethane/methanol (10:1, v/v), dried over anhydrous Na_2_SO_4_, and purified using column chromatography to afford target compound **6a** (120 mg, 59%). Compound **6b**–**c** was prepared by a procedure similar to that used for compound **6a**.

#### *tert*-Butyl 3-(4-(4-(3-Benzyl-1-((1*r*,4*r*)-4-((5-cyanopyridin-2-yl)amino)cyclohexyl)ureido)phenyl)piperazine-1-carbonyl)azetidine-1-carboxylate (**6a**).

^1^H NMR (500 MHz, DMSO-*d*_6_) δ 8.29 (d, *J* = 2.1 Hz, 1H), 7.60 (d, *J* = 9.2 Hz, 1H), 7.48 (s, 1H), 7.28–7.25 (m, 2H), 7.18–7.14 (m, 3H), 7.03–6.99 (m, 4H), 6.46 (d, *J* = 8.9 Hz, 2H), 5.61–5.59 (m, 1H), 4.28–4.23 (m, 1H), 4.14 (d, *J* = 5.8 Hz, 2H), 3.72–3.68 (m, 1H), 3.63–3.60 (m, 4H), 3.40 (s, 2H), 3.19–3.17 (m, 3H), 3.16–3.12 (m, 4H), 1.90 (d, *J* = 10.9 Hz, 2H), 1.76 (d, *J* = 12.3 Hz, 2H), 1.37 (s, 9H), 1.32–1.27 (m, 2H), 1.13–1.06 (m, 2H). MS (ESI) for C_39_H_49_N_8_O_4_ [M + H]^+^, calcd: 693.4, found: 692.8.

#### *tert*-Butyl 3-(4-(4-(3-Benzyl-1-((1*r*,4*r*)-4-((5-cyanopyridin-2-yl)amino)cyclohexyl)ureido)phenyl)piperazine-1-carbonyl)pyrrolidine-1-carboxylate (**6b**)

^1^H NMR (500 MHz, DMSO-*d*_6_) δ 8.29 (dd, *J* = 2.3, 0.7 Hz, 1H), 7.60 (dd, *J* = 9.0, 2.1 Hz, 1H), 7.47 (d, *J* = 7.6 Hz, 1H), 7.29–7.24 (m, 2H), 7.20–7.14 (m, 3H), 7.06–6.98 (m, 4H), 6.46 (d, *J* = 8.9 Hz, 1H), 5.61–5.55 (m, 1H), 4.29–4.24 (m, 1H), 4.14 (d, *J* = 6.0 Hz, 2H), 3.67 (s, 2H), 3.62 (d, *J* = 6.0 Hz, 2H), 3.45–3.40 (m, 3H), 3.27–3.15 (m, 5H), 2.04 (d, *J* = 8.8 Hz, 1H), 1.96–1.89 (m, 3H), 1.77 (d, *J* = 12.0 Hz, 2H), 1.40 (s, 9H), 1.34–1.23 (m, 4H), 1.13–1.06 (m, 2H).

#### *tert*-Butyl 4-(4-(4-(3-Benzyl-1-((1*r*,4*r*)-4-((5-cyanopyridin-2-yl)amino)cyclohexyl)ureido)phenyl)piperazine-1-carbonyl)piperidine-1-carboxylate (**6c**)

^1^H NMR (500 MHz, DMSO-*d*_6_) δ 8.29 (s, 1H), 7.60 (d, *J* = 9.2 Hz, 1H), 7.48 (d, *J* = 9.5 Hz, 1H), 7.26 (m, 2H), 7.19–7.13 (m, 3H), 7.05–6.99 (m, 4H), 6.46 (d, *J* = 9.0 Hz, 1H), 5.59 (t, *J* = 5.6 Hz, 1H), 4.25 (m, 1H), 4.14 (d, *J* = 5.8 Hz, 2H), 3.94 (s, 2H), 3.67 (s, 2H), 3.59 (s, 2H), 3.21 (s, 2H), 3.16 (s, 2H), 2.92–2.74 (m, 3H), 1.90 (d, *J* = 11.6 Hz, 2H), 1.76 (d, *J* = 12.9 Hz, 2H), 1.62 (d, *J* = 11.5 Hz, 2H), 1.44–1.36 (m, 12H), 1.35–1.23 (m, 3H), 1.13–1.04 (m, 2H).

### General Procedure for the Synthesis of **8a**–d

To a stirred solution of compound **5** (153 mg, 0.3 mmol) in anhydrous CH_2_Cl_2_ (3 mL) were added 1-(*tert*-butoxycarbonyl)-3-azetidinecarboxaldehyde (61 mg, 0.33 mmol), CH_3_COOH (17 μL) and sodium triacetoxyborohydride (127 mg, 0.6 mmol). The resulted suspension was then backfilled with argon (3 cycles) and stirred for 8 h until completed as indicated by TLC. After quenching with sodium bicarbonate solution, the mixture was extracted with dichloromethane/methanol (10:1, v/v). The organic layer was washed with brine, dried over anhydrous Na_2_SO_4_, concentrated under reduced pressure and purified using column chromatography to afford target compound **8a** (135 mg, 40%). Compounds **8b**–**d** were prepared by a procedure similar to that used for compound **8a**.

#### *tert*-Butyl 3-((4-(4-(3-Benzyl-1-((1*r*,4*r*)-4-((5-cyanopyridin-2-yl)amino)cyclohexyl)ureido)phenyl)piperazin-1-yl)methyl)azetidine-1-carboxylate (**8a**)

^1^H NMR (500 MHz, DMSO-*d*_6_) δ 8.29 (d, *J* = 2.3 Hz, 1H), 7.62–7.58 (m, 1H), 7.46 (s, 1H), 7.26 (m, 2H), 7.16 (m, 3H), 6.98 (m, 4H), 6.46 (d, *J* = 8.9 Hz, 1H), 5.57 (t, *J* = 6.0 Hz, 1H), 4.25 (m, 1H), 4.14 (d, *J* = 6.1 Hz, 2H), 3.91 (s, 2H), 3.48 (m, 3H), 3.15 (d, *J* = 5.3 Hz, 4H), 2.75 (m, 1H), 2.56 (d, *J* = 7.4 Hz, 2H), 2.47 (m, 2H), 1.90 (d, *J* = 12.0 Hz, 2H), 1.76 (d, *J* = 12.1 Hz, 2H), 1.37 (m, 11H), 1.30 (q, *J* = 12.8 Hz, 2H), 1.09 (q, *J* = 12.5 Hz, 2H).

#### *tert*-Butyl (*S*)-3-((4-(4-(3-Benzyl-1-((1*r*,4*s*)-4-((5-cyanopyridin-2-yl)amino)cyclohexyl)ureido)phenyl)piperazin-1-yl)methyl)pyrrolidine-1-carboxylate (**8b**)

^1^H NMR (500 MHz, DMSO-*d*_6_) δ 8.29 (s, 1H), 7.60 (d, *J* = 8.8 Hz, 1H), 7.48 (s, 1H), 7.28–7.25 (m, 2H), 7.18–7.14 (m, 3H), 7.01–6.95 (m, 4H), 6.46 (d, *J* = 8.7 Hz, 1H), 5.58 (s, 1H), 4.30–4.21 (m, 1H), 4.14 (d, *J* = 5.4 Hz, 2H), 3.42–3.36 (m, 1H), 3.31 (s, 1H), 3.22–3.14 (m, 5H), 2.92 (s, 1H), 2.58–2.52 (m, 2H), 2.31 (d, *J* = 4.4 Hz, 2H), 1.90 (d, *J* = 9.7 Hz, 2H), 1.76 (d, *J* = 9.9 Hz, 2H), 1.58–1.49 (m, 1H), 1.39 (s, 9H), 1.36–1.22 (m, 4H), 1.15–1.04 (m, 2H), 0.88–0.80 (m, 3H).

#### *tert*-Butyl (*R*)-3-((4-(4-(3-Benzyl-1-((1*r*,4*r*)-4-((5-cyanopyridin-2-yl)amino)cyclohexyl)ureido)phenyl)piperazin-1-yl)methyl)pyrrolidine-1-carboxylate (**8c**)

^1^H NMR (500 MHz, DMSO-*d*_6_) δ 8.29 (d, *J* = 2.2 Hz, 1H), 7.60 (d, *J* = 8.0 Hz, 1H), 7.48 (d, *J* = 4.9 Hz, 1H), 7.28–7.25 (m, 2H), 7.18–7.14 (m, 3H), 7.02–6.95 (m, 4H), 6.46 (d, *J* = 9.2 Hz, 1H), 5.59 (d, *J* = 4.4 Hz, 1H), 4.30–4.22 (m, 1H), 4.14 (d, *J* = 6.0 Hz, 2H), 3.41–3.37 (m, 2H), 3.33–3.28 (m, 1H), 3.22–3.14 (m, 5H), 2.95–2.88 (m, 1H), 2.57–2.52 (m, 3H), 2.49–2.44 (m, 1H), 2.31 (d, *J* = 7.3 Hz, 2H), 1.91–1.89 (m, 3H), 1.76 (d, *J* = 10.7 Hz, 2H), 1.57–1.50 (m, 1H), 1.39 (s, 9H), 1.34–1.22 (m, 2H), 1.15–1.05 (m, 2H).

#### *tert*-Butyl 4-((4-(4-(3-Benzyl-1-((1*r*,4*r*)-4-((5-cyanopyridin-2-yl)amino)cyclohexyl)ureido)phenyl)piperazin-1-yl)methyl)piperidine-1-carboxylate (**8d**)

^1^H NMR (500 MHz, DMSO-*d*_6_) δ 8.29 (d, *J* = 2.1 Hz, 1H), 7.60 (d, *J* = 8.7 Hz, 1H), 7.49 (d, *J* = 6.6 Hz, 1H), 7.28–7.26 (m, 2H), 7.18–7.14 (m, 3H), 7.03–6.96 (m, 4H), 6.46 (d, *J* = 8.8 Hz, 1H), 5.59 (t, *J* = 5.6 Hz, 1H), 4.28–4.23 (s, 1H), 4.14–4.13(d, *J* = 6.0 Hz, 2H), 3.92 (s, 2H), 3.50 (s, 1H), 3.17 (s, 4H), 2.47 (s, 4H), 2.17 (d, *J* = 6.6 Hz, 2H), 1.90 (d, *J* = 9.7 Hz, 2H), 1.77–1.68 (m, 5H), 1.39 (s, 9H), 1.33–1.26 (m, 2H), 1.13–1.08 (m, 2H), 0.98–0.94 (m, 2H), 0.84–0.81 (m, 2H).

#### *tert*-Butyl ((1*r*,4*r*)-4-((4-Bromophenyl)amino)cyclohexyl)carbamate (**9**)

To a solution of 1-bromo-4-iodobenzene (5.0 g, 17.7 mmol) in anhydrous toluene (150 mL) were added Pd_2_(dba)_3_ (1.37 g, 1.5 mmol), Xantphos (1.7 g, 2.94 mmol), *tert*-BuONa (2.8 g, 29.4 mmol) and *trans*-*N*-Boc-1,4-cyclohexanedimine (3.2 g, 14.7 mmol). The resulting suspension was then evacuated and backfilled with argon (3 cycles) and heated at 100 °C overnight under stirring. After cooling, the reaction solution was filtered. The solvent was evaporated under reduced pressure and purified using column chromatography to afford target compound **9** (1.6 g, 29%). ^1^H NMR (500 MHz, DMSO-*d*_6_) δ 7.16–7.13 (m, 2H), 6.79 (d, *J* = 7.9 Hz, 1H), 6.49 (dd, *J* = 8.9, 2.0 Hz, 2H), 5.63 (d, *J* = 8.0 Hz, 1H), 3.21–3.19 (m, 1H), 3.06–3.03 (m, 1H), 1.93 (d, *J* = 12.6 Hz, 2H), 1.77 (d, *J* = 12.5 Hz, 2H), 1.28–1.21 (m, 1H), 1.16–1.11 (m, 2H).

#### *tert*-Butyl ((1*r*,4*r*)-4-(3-Benzyl-1-(4-bromophenyl)ureido)cyclohexyl) carbamate (**10**)

To a solution of compound **9** (3.9 g, 10.6 mmol) in anhydrous DMF (4 mL) were added benzyl isocyanate (4.2 g, 31.7 mmol) and DIPEA (1.59 g, 12.3 mmol). The mixture was heated at 95 °C overnight. After the reaction completed as indicated by TLC, the reaction mixture was filtered. The solvent was evaporated under reduced pressure and purified using column chromatography to afford target compound **10** (3.2 g, 59%).

^1^H NMR (500 MHz, DMSO-*d*_6_) δ 7.64–7.62 (m, 2H), 7.27–7.25 (m, 2H), 7.19–7.15 (m, 3H), 7.12–7.10 (m, 2H), 6.69 (d, *J* = 7.9 Hz, 1H), 6.01 (t, *J* = 6.1 Hz, 1H), 4.24–4.16 (m, 1H), 4.13 (d, *J* = 6.1 Hz, 2H), 2.94 (m, 1H), 1.72 (d, *J* = 11.7 Hz, 4H), 1.34 (s, 9H), 1.24–1.21 (m, 2H), 1.03–0.96 (d, *J* = 12.1 Hz, 2H).

#### 1-((1*r*,4*r*)-4-Aminocyclohexyl)-3-benzyl-1-(4-bromophenyl)urea (**11**)

To a solution of compound **10** (2.0 g, 4 mmol) in anhydrous CH_2_Cl_2_ (2 mL) was added TFA (1 mL). The mixture was stirred for 1 h. Then the reaction was evaporated under reduced pressure and purified using column chromatography to afford target compound **11** (656 mg, 41%). ^1^H NMR (500 MHz, DMSO-*d*_6_) δ 7.65–7.63 (m, 2H), 7.28–7.25 (m, 2H), 7.19–7.11 (m, 5H), 6.04 (m, 1H), 4.23–4.18 (m, 1H), 4.12 (d, *J* = 5.3 Hz, 2H), 2.79 (m, 1H), 1.89 (d, *J* = 12.8 Hz, 2H), 1.79 (d, *J* = 12.2 Hz, 2H), 1.38 (m, 2H), 1.09–1.01 (m, 2H).

#### 3-Benzyl-1-(4-bromophenyl)-1-((1*r*,4*r*)-4-((5-cyanopyridin-2-yl)amino)cyclohexyl)urea (**12**)

To a stirred solution of compound **11** (4.01 g, 10 mmol) in anhydrous DMF (20 mL) were added Cs_2_CO_3_ (3.9 g, 12 mmol) and 5-cyano-2-fluoropyridine (1.22 g, 10 mmol). The resulting solution was stirred overnight at room temperature. After the reaction completed as indicated by TLC, the mixture was filtered. The solvent was evaporated under reduced pressure and the residues were purified using column chromatography to afford target compound **12** (4.5 g, 89%). ^1^H NMR (500 MHz, DMSO-*d*_6_) δ 8.30 (d, *J* = 2.2 Hz, 1H), 7.65–7.60 (m, 3H), 7.48 (d, *J* = 6.5 Hz, 1H), 7.29–7.26 (m, 2H), 7.19–7.13 (m, 5H), 6.47 (d, *J* = 8.9 Hz, 1H), 6.03 (t, *J* = 5.9 Hz, 1H), 4.30–4.23 (m, 1H), 4.14 (d, *J* = 6.0 Hz, 2H), 3.51 (s, 1H), 1.91 (d, *J* = 11.0 Hz, 1H), 1.78 (d, *J* = 11.4 Hz, 2H), 1.31 (m, 2H), 1.10 (m, 2H).

#### 4-((1-(4-(3-Benzyl-1-((1*r*,4*r*)-4-((5-cyanopyridin-2-yl)amino)cyclohexyl)ureido)phenyl)azetidin-3-yl)methyl)piperazine-1-carboxylate (**14a**)

To a solution of compound **12** (201 mg, 0.4 mmol) in anhydrous toluene (5 mL) were added Pd_2_(dba)_3_ (37 mg, 0.04 mmol), Xantphos (46 mg, 0.08 mmol), compound **13** (216 mg) and *tert*-BuONa (77 mg, 0.8 mmol). The resulting suspension was then evacuated and backfilled with argon (3 cycles) and heated at 110 °C for 4 h under stirring. After cooling, the reaction solution was filtered. The solvent was evaporated under reduced pressure and purified using column chromatography to afford target compound **14a**. ^1^H NMR (500 MHz, DMSO-*d*_6_) δ 8.29 (d, *J* = 2.3 Hz, 1H), 7.60 (d, *J* = 8.9 Hz, 1H), 7.47 (d, *J* = 7.8 Hz, 1H), 7.40–7.32 (m, 5H), 7.28–7.25 (m, 2H), 7.18–7.14 (m, 3H), 6.95 (d, *J* = 8.5 Hz, 2H), 6.47–6.43 (m, 3H), 5.48 (t, *J* = 5.9 Hz, 1H), 5.07 (s, 2H), 4.29–4.21 (m, 1H), 4.14 (d, *J* = 6.1 Hz, 2H), 3.94 (t, *J* = 7.5 Hz, 2H), 3.48 (t, *J* = 6.4 Hz, 2H), 3.39 (s, 4H), 2.96–2.88 (m, 1H), 2.59 (d, *J* = 7.4 Hz, 2H), 2.36 (s, 4H), 1.90 (d, *J* = 12.1 Hz, 2H), 1.75 (d, *J* = 12.1 Hz, 2H), 1.31–1.28 (m, 2H), 1.26–1.23 (m, 3H), 1.12–1.04 (m, 2H).

### General Procedure for the Synthesis of **14b**–i

To a solution of compound **12** (503 mg, 1 mmol) in anhydrous toluene (10 mL) were added Pd_2_(dba)_3_ (92 mg, 0.1 mmol), Xantphos (115 mg, 0.2 mmol), *tert*-butyl 4-(pyrrolidin-3-ylmethyl)piperazine-1-carboxylate (323 mg, 1.2 mmol) and *tert*-BuONa (192 mg, 2 mmol). The resulting suspension was then evacuated and backfilled with argon (3 cycles) and heated at 110 °C overnight under stirring. After cooling, the reaction solution was filtered. The solvent was evaporated under reduced pressure and purified using column chromatography to afford target compound **14b** (126 mg, 18%). Compounds **14c-i** were prepared by a procedure similar to that used for compound **14b**.

#### *tert*-Butyl 4-((1-(4-(3-Benzyl-1-((1*r*,4*r*)-4-((5-cyanopyridin-2-yl)amino)cyclohexyl)ureido)phenyl)pyrrolidin-3-yl)methyl)piperazine-1-carboxylate (**14b**)

^1^H NMR (500 MHz, DMSO-*d*_6_) δ 8.29 (d, *J* = 2.3 Hz, 1H), 7.60 (dd, *J* = 9.0, 2.4 Hz, 1H), 7.47 (d, *J* = 7.6 Hz, 1H), 7.28–7.25 (m, 2H), 7.20–7.13 (m, 3H), 6.95 (d, *J* = 8.6 Hz, 2H), 6.54 (d, *J* = 8.5 Hz, 2H), 6.46 (d, *J* = 8.9 Hz, 1H), 5.45 (t, *J* = 6.1 Hz, 1H), 4.30–4.22 (m, 1H), 4.14 (d, *J* = 6.1 Hz, 2H), 3.47 (s, 1H), 3.39–3.34 (m, 2H), 3.31 (s, 4H), 3.25–3.01 (m, 1H), 3.00 (dd, *J* = 9.5, 6.2 Hz, 1H), 2.59–2.53 (m, 1H), 2.38–2.32 (m, 6H), 2.11–2.06 (m, 1H), 1.90 (d, *J* = 12.0 Hz, 2H), 1.80–1.66 (m, 3H), 1.39 (s, 9H), 1.34–1.26 (m, 2H), 1.14–1.10 (m, 2H).

#### *tert*-Butyl 4-((1-(4-(3-Benzyl-1-((1*r*,4*r*)-4-((5-cyanopyridin-2-yl)amino)cyclohexyl)ureido)phenyl)piperidin-4-yl)methyl)piperazine-1-carboxylate (**14c**)

^1^H NMR (500 MHz, DMSO-*d*_6_) δ 8.29 (s, 1H), 7.60 (d, *J* = 9.3 Hz, 1H), 7.47 (d, *J* = 8.5 Hz, 1H), 7.28–7.25 (m, 2H), 7.18–7.14 (m, 3H), 6.99–6.95 (m, 4H), 6.46 (d, *J* = 9.6 Hz, 1H), 5.58–5.52 (m, 1H), 4.29–4.21 (m, 1H), 4.14 (d, *J* = 5.6 Hz, 2H), 3.72 (d, *J* = 11.8 Hz, 2H), 3.30 (s, 4H),2.67 (t, *J* = 11.5 Hz, 2H), 2.29 (s, 4H), 2.16 (d, *J* = 6.2 Hz, 2H), 1.90 (d, *J* = 9.8 Hz, 2H), 1.81–1.75 (m, 4H), 1.39 (s, 9H), 1.33–1.23 (m, 4H), 1.21–1.06 (m, 4H).

#### *tert*-Butyl 3-(4-(4-(3-Benzyl-1-((1*r*,4*r*)-4-((5-cyanopyridin-2-yl)amino)cyclohexyl)ureido)phenyl)piperazin-1-yl)azetidine-1-carboxylate (**14d**)

^1^H NMR (500 MHz, DMSO-*d*_6_) δ 8.29 (d, *J* = 2.3 Hz, 1H), 7.60 (d, *J* = 9.0 Hz, 1H), 7.47 (s, 1H), 7.28–7.25 (m, 2H), 7.18–7.15 (m, 3H), 7.03–6.96 (m, 4H), 6.46 (d, *J* = 8.9 Hz, 1H), 5.57 (d, *J* = 6.6 Hz, 1H), 4.26 (t, *J* = 12.2 Hz, 1H), 4.14 (d, *J* = 6.0 Hz, 2H), 3.86 (s, 2H), 3.71 (s, 2H), 3.19 (s, 4H), 3.08 (s, 1H), 2.43 (s, 4H), 1.90 (d, *J* = 12.0 Hz, 2H), 1.76 (d, *J* = 12.1 Hz, 2H), 1.38 (s, 10H), 1.34–1.26 (m, 3H), 1.13–1.06 (m, 2H).

#### *tert*-Butyl 3-(4-(4-(3-Benzyl-1-((1*r*,4*r*)-4-((5-cyanopyridin-2-yl)amino)cyclohexyl)ureido)phenyl)piperazin-1-yl)pyrrolidine-1-carboxylate (**14e**)

^1^H NMR (500 MHz, DMSO-*d*_6_) δ 8.29 (d, *J* = 2.5 Hz, 1H), 7.60 (d, *J* = 8.9 Hz, 1H), 7.50 (d, *J* = 17.1 Hz, 1H), 7.28–7.25 (m, 2H), 7.18–7.14 (m, 3H), 7.01–6.96 (m, 4H), 6.46 (d, *J* = 8.9 Hz, 1H), 5.60 (d, *J* = 6.4 Hz, 1H), 4.28–4.23 (m, 1H), 4.14 (d, *J* = 6.0 Hz, 2H), 3.53 (d, *J* = 8.5 Hz, 1H), 3.42–3.38 (m, 2H), 3.18 (s, 5H), 3.02–2.95 (m, 1H), 2.81–2.76 (m, 1H), 2.63–2.55 (m, 2H), 2.06 (s, 1H), 1.90 (d, *J* = 11.6 Hz, 2H), 1.76 (d, *J* = 12.0 Hz, 2H), 1.71–1.64 (m, 1H), 1.40 (s, 9H), 1.353– 1.24 (m, 2H), 1.14–1.04 (m, 2H).

#### *tert*-Butyl 4-(4-(4-(3-Benzyl-1-((1*r*,4*r*)-4-((5-cyanopyridin-2-yl)amino)cyclohexyl)ureido)phenyl)piperazin-1-yl)piperidine-1-carboxylate (**14f**)

^1^H NMR (500 MHz, DMSO-*d*_6_) δ 8.29 (d, *J* = 2.3 Hz, 1H), 7.60 (d, *J* = 9.0 Hz, 1H), 7.48 (s, 1H), 7.28–7.25 (m, 2H), 7.18–7.14 (m, 3H), 7.01–6.94 (m, 4H), 6.46 (d, *J* = 8.9 Hz, 1H), 5.59 (s, 1H), 4.29–4.20 (m, 1H), 4.14 (d, *J* = 6.1 Hz, 2H), 3.98 (d, *J* = 28.3 Hz, 2H), 3.17–3.15 (m, 4H), 2.72 (s, 1H), 2.62–2.60 (m, 4H), 2.40 (t, *J* = 11.3 Hz, 1H), 1.90 (d, *J* = 11.8 Hz, 2H), 1.77 (d, *J* = 11.7 Hz, 4H), 1.39 (s, 10H), 1.33–1.22(m, 5H), 1.14–1.05 (m, 2H).

#### *tert*-Butyl 7-(4-(3-Benzyl-1-((1*r*,4*r*)-4-((5-cyanopyridin-2-yl)amino)cyclohexyl)ureido)phenyl)-2,7-diazaspiro[3.5]nonane-2-carboxylate (**14g**)

^1^H NMR (500 MHz, DMSO-*d*_6_) δ 8.28 (t, *J* = 1.9 Hz, 1H), 7.59 (d, *J* = 9.2 Hz, 1H), 7.45 (s, 1H), 7.28–7.20 (m, 2H), 7.17–7.13 (m, 3H), 6.96–6.92 (m, 4H), 6.45 (d, *J* = 9.0 Hz, 1H), 5.53 (d, *J* = 6.6 Hz, 1H), 4.26–4.21 (m, 1H), 4.13 (d, *J* = 6.2 Hz, 2H), 3.58 (s, 3H), 3.29 (s, 2H), 3.14 (s, 4H), 1.88 (d, *J* = 12.2 Hz, 2H), 1.76–1.74 (m, 6H), 1.37 (s, 9H), 1.31–1.22 (m, 2H), 1.07 (d, *J* = 12.7 Hz, 2H).

#### *tert*-Butyl 8-(4-(3-Benzyl-1-((1*r*,4*r*)-4-((5-cyanopyridin-2-yl)amino)cyclohexyl)ureido)phenyl)-2,8-diazaspiro[4.5]decane-2-carboxylate (**14h**)

^1^H NMR (500 MHz, DMSO-*d*_6_) δ 8.29 (d, *J* = 2.3 Hz, 1H), 7.62–7.58 (m, 1H), 7.47 (d, *J* = 7.5 Hz, 1H), 7.28–7.25 (m, 2H), 7.19–7.13 (m, 3H), 6.99 (s, 4H), 6.46 (d, *J* = 8.8 Hz, 1H), 5.54 (d, *J* = 6.4 Hz, 1H), 4.28–4.23 (m, 1H), 4.15 (d, *J* = 6.0 Hz, 2H), 3.31–3.28(m, 2H), 3.23–3.19 (m, 3H), 3.12 (s, 2H), 1.93–1.87 (m, 2H), 1.79–1.71 (m, 4H), 1.61–1.58 (m, 4H), 1.40 (s, 9H), 1.38–1.22 (m, 4H), 1.13–1.06 (m, 2H).

#### *tert*-Butyl 9-(4-(3-Benzyl-1-((1*r*,4*r*)-4-((5-cyanopyridin-2-yl)amino)cyclohexyl)ureido)phenyl)-3,9-diazaspiro[5.5]undecane-3-carboxylate (**14i**)

^1^H NMR (500 MHz, DMSO-*d*_6_) δ 8.29 (d, *J* = 2.3 Hz, 1H), 7.60 (d, *J* = 8.9 Hz, 1H), 7.47 (d, *J* = 7.6 Hz, 1H), 7.28–7.25 (m, 2H), 7.19–7.13 (m, 3H), 6.99–6.95 (m, 4H), 6.46 (d, *J* = 8.8 Hz, 1H), 5.53 (t, *J* = 6.0 Hz, 1H), 4.28–4.23 (m, 1H), 4.14 (d, *J* = 6.0 Hz, 2H), 3.33 (s, 2H), 3.18 (s, 4H), 1.90 (d, *J* = 11.9 Hz, 2H), 1.76 (d, *J* = 12.1 Hz, 2H), 1.58–1.56 (m, 4H), 1.41–1.39 (m, 14H), 1.35–1.21 (m, 4H), 1.09 (q, *J* = 12.6 Hz, 2H).

### General Procedure for the Synthesis of **4a**–**g**, **4i**–**p**, **4g-F**, **ZLC491** and **ZLC491N**

To a stirred solution of compound **6a** in anhydrous CH_2_Cl_2_ (1 mL) was added TFA (0.5 mL). The mixture was stirred for 1 h at room temperature. The reaction solution was concentrated under reduced pressure, and then saturated Na_2_CO_3_ solution was added and extracted with dichloromethane/methanol (10:1, v/v). The organic layer was washed with brine, dried over anhydrous Na_2_SO_4_, and then concentrated under reduced pressure. The residue was used directly in the next step without further purification. To a solution of the residue in DMF were added DIPEA (3 eq) and compound **7a** (1 eq). The resulting solution was heated at 100 °C for 2 h under stirring. Upon cooling, the solvent was removed in vacuo. The residue was then purified by silica gel column chromatography to yield target compound **4a** (31 mg, 20%). Compounds **4b**–**g**, **4i**–**p**, **4g-F**, **ZLC491** and **ZLC491N** were prepared by a procedure similar to that used for compound **4a**.

#### 3-Benzyl-1-((1*r*,4*r*)-4-((5-cyanopyridin-2-yl)amino)cyclohexyl)-1-(4-(4-(1-(2-(2,6-dioxopiperidin-3-yl)-1,3-dioxoisoindolin-5-yl)azetidine-3-carbonyl)piperazin-1-yl)phenyl)urea (**4a**)

^1^H NMR (600 MHz, DMSO-*d*_6_) δ 11.07 (s, 1H), 8.29 (d, *J* = 2.2 Hz, 1H), 7.66 (d, *J* = 8.3 Hz, 1H), 7.60 (d, *J* = 8.2 Hz, 1H), 7.51–7.44 (m, 1H), 7.28–7.26 (m, 2H), 7.19–7.15 (m, 3H), 7.05–7.01 (m, 4H), 6.84 (d, *J* = 1.8 Hz, 1H), 6.70 (dd, *J* = 8.4, 2.0 Hz, 1H), 6.47 (d, *J* = 8.9 Hz, 1H), 5.58 (t, *J* = 5.5 Hz, 1H), 5.06 (dd, *J* = 12.8, 5.4 Hz, 1H), 4.28–4.25 (m, 3H), 4.18–4.15 (m, 4H), 4.00–3.97 (m, 1H), 3.65 (s, 2H), 3.49 (s, 3H), 3.24–3.21 (m, 4H), 2.91–2.84 (m, 1H), 2.59–2.53 (m, 2H), 2.03–2.00 (m, 1H), 1.91 (d, *J* = 10.3 Hz, 2H), 1.77 (d, *J* = 10.6 Hz, 2H), 1.33–1.28 (m, 2H), 1.13–1.07 (m, 2H). ^13^C NMR (151 MHz, DMSO) δ 172.80, 170.10, 169.35, 167.43, 167.15, 159.24, 156.77, 154.98, 153.10, 149.92, 141.30, 138.40, 133.80, 131.60, 128.76, 128.03, 126.71, 126.25, 124.83, 119.12, 117.24, 115.97, 114.30, 108.62, 104.56, 94.00, 53.63, 52.92, 48.72, 48.07, 47.75, 44.42, 43.48, 41.30, 31.30, 31.21, 30.98, 30.20, 22.19. HRMS (ESI) for C_47_H_48_N_10_O_6_ [M + H]^+^, calcd: 849.3831, found: 849.3839. HPLC purity: 98.84%.

#### 3-Benzyl-1-((1*r*,4*r*)-4-((5-cyanopyridin-2-yl)amino)cyclohexyl)-1-(4-(4-(1-(2-(2,6-dioxopiperidin-3-yl)-1,3-dioxoisoindolin-5-yl)pyrrolidine-3-carbonyl)piperazin-1-yl)phenyl)urea (**4b**)

^1^H NMR (600 MHz, DMSO-*d*_6_) δ 11.07 (s, 1H), 8.29 (d, *J* = 2.3 Hz, 1H), 7.65 (d, *J* = 8.4 Hz, 1H), 7.62–7.58 (m, 1H), 7.51–7.44 (m, 1H), 7.27 (dd, *J* = 8.3, 6.9 Hz, 2H), 7.20–7.14 (m, 3H), 7.07–7.01 (m, 4H), 6.94 (d, *J* = 2.2 Hz, 1H), 6.87–6.82 (m, 1H), 6.47 (d, *J* = 8.9 Hz, 1H), 5.58 (t, *J* = 6.1 Hz, 1H), 5.06 (dd, *J* = 12.7, 5.5 Hz, 1H), 4.30–4.23 (m, 1H), 4.15 (d, *J* = 6.0 Hz, 2H), 3.74 (q, *J* = 5.8 Hz, 2H), 3.70–3.62 (m, 4H), 3.58–3.44 (m, 4H), 3.28 (d, *J* = 5.3 Hz, 2H), 3.20 (d, *J* = 5.7 Hz, 2H), 2.92–2.84 (m, 1H), 2.60–2.52 (m, 2H), 2.29–2.22 (m, 1H), 2.20–2.14 (m, 1H), 2.04–1.99 (m, 1H), 1.91 (d, *J* = 11.7 Hz, 2H), 1.81–1.74 (m, 2H), 1.34–1.27 (m, 2H), 1.13–1.07 (m, 2H). ^13^C NMR (151 MHz, DMSO) δ 172.83, 170.43, 170.16, 167.71, 167.24, 159.25, 156.79, 153.11, 151.66, 149.94, 141.30, 138.42, 134.00, 131.61, 128.72, 128.04, 126.71, 126.26, 124.95, 119.13, 115.93, 115.78, 115.44, 109.12, 105.67, 94.01, 52.93, 50.59, 48.70, 48.33, 47.75, 47.52, 44.80, 43.49, 41.35, 31.31, 30.97, 30.21, 28.51, 22.25, 22.07. HRMS (ESI) for C_48_H_50_N_10_O_6_ [M + H]^+^, calcd: 863.3988, found: 863.3993. HPLC purity: 98.54%.

#### 3-Benzyl-1-((1*r*,4*r*)-4-((5-cyanopyridin-2-yl)amino)cyclohexyl)-1-(4-(4-(1-(2-(2,6-dioxopiperidin-3-yl)-1,3-dioxoisoindolin-5-yl)piperidine-4-carbonyl)piperazin-1-yl)phenyl)urea (**4c**)

^1^H NMR (500 MHz, DMSO-*d*_6_) δ 11.10 (s, 1H), 8.30 (d, *J* = 1.8 Hz, 1H), 7.67 (d, *J* = 8.5 Hz, 1H), 7.60 (d, *J* = 8.5 Hz, 1H), 7.54–7.46 (m, 1H), 7.34 (s, 1H), 7.28–7.24 (m, 3H), 7.19–7.15 (m, 3H), 7.05–7.00 (m, 4H), 6.47 (d, *J* = 8.9 Hz, 1H), 5.60–5.59 (m, 1H), 5.07 (dd, *J* = 12.8, 5.3 Hz, 1H), 4.29–4.24 (m, 1H), 4.15 (d, *J* = 5.5 Hz, 2H), 4.08 (d, *J* = 12.6 Hz, 2H), 3.71 (s, 2H), 3.61 (s, 2H), 3.49 (s, 1H), 3.23 (s, 2H), 3.17 (s, 2H), 3.11–3.02 (m, 3H), 2.92–2.85 (m, 1H), 2.60–2.54 (m, 2H), 2.03–2.00 (m, 1H), 1.90 (d, *J* = 10.5 Hz, 2H), 1.78–1.73 (m, 4H), 1.67–1.60 (m, 2H), 1.33–1.27 (m, 2H), 1.13–1.06 (m, 2H). ^13^C NMR (151 MHz, DMSO) δ 172.81, 172.34, 170.10, 167.60, 166.96, 159.24, 156.77, 154.82, 153.09, 149.92, 141.30, 138.33, 134.05, 131.60, 128.70, 128.02, 126.70, 126.24, 125.03, 119.11, 117.64, 117.62, 115.88, 108.96, 107.85, 93.99, 52.92, 48.73, 48.41, 47.84, 46.72, 44.62, 43.48, 41.07, 36.80, 31.30, 30.98, 30.20, 27.42, 22.19. HRMS (ESI) for C_49_H_52_N_10_O_6_ [M + H]^+^, calcd: 877.4144, found: 877.4139. HPLC purity: 99.24%.

#### 3-Benzyl-1-((1*r*,4*r*)-4-((5-cyanopyridin-2-yl)amino)cyclohexyl)-1-(4-(4-((1-(2-(2,6-dioxopiperidin-3-yl)-1,3-dioxoisoindolin-5-yl)azetidin-3-yl)methyl)piperazin-1-yl)phenyl)urea (**4d**)

^1^H NMR (500 MHz, DMSO-*d*_6_) δ 11.07 (s, 1H), 8.29 (d, *J* = 2.2 Hz, 1H), 7.64 (d, *J* = 8.3 Hz, 1H), 7.60 (d, *J* = 10.2 Hz, 1H), 7.47 (d, *J* = 7.0 Hz, 1H), 7.28–7.25 (m, 2H), 7.18–7.15 (m, 3H), 7.02–6.97 (m, 4H), 6.78 (d, *J* = 1.5 Hz, 1H), 6.65 (dd, *J* = 8.4, 1.7 Hz, 1H), 6.47 (d, *J* = 8.8 Hz, 1H), 5.59–5.69 (m, 1H), 5.07–5.03 (m, 1H), 4.29–4.24 (m, 1H), 4.17–4.14 (m, 4H), 3.73–3.70 (m, 2H), 3.50 (s, 1H), 3.50–3.46 (m, 1H),3.19 (s, 4H), 3.05 (s, 1H), 2.90–2.84 (m, 1H), 2.66 (d, *J* = 5.4 Hz, 2H), 2.60–2.54 (m, 6H), 2.02–2.00 (m, 1H), 1.91 (d, *J* = 9.7 Hz, 2H), 1.77 (d, *J* = 10.2 Hz, 2H), 1.33–1.26 (m, 2H), 1.13–1.06 (m, 2H). ^13^C NMR (151 MHz, DMSO) δ 172.80, 170.11, 167.49, 167.18, 159.24, 156.80, 155.19, 153.10, 150.12, 141.34, 138.34, 133.82, 131.50, 128.22, 128.02, 126.69, 126.23, 124.80, 119.12, 116.68, 115.42, 114.06, 108.80, 104.35, 93.98, 61.76, 55.75, 52.90, 52.85, 48.70, 48.63, 47.63, 43.46, 31.30, 30.98, 30.20, 29.01, 27.00, 22.21. HRMS (ESI) for C_47_H_50_N_10_O_5_ [M + H]^+^, calcd: 835.4038, found: 835.4043. HPLC purity: 99.75%.

#### 3-Benzyl-1-((1*r*,4*s*)-4-((5-cyanopyridin-2-yl)amino)cyclohexyl)-1-(4-(4-(((3*S*)-1-(2-(2,6-dioxopiperidin-3-yl)-1,3-dioxoisoindolin-5-yl)pyrrolidin-3-yl)methyl)piperazin-1-yl)phenyl)urea (**4e**)

^1^H NMR (600 MHz, DMSO-*d*_6_) δ 11.07 (s, 1H), 8.29 (d, *J* = 2.2 Hz, 1H), 7.64 (d, *J* = 8.4 Hz, 1H), 7.60 (d, *J* = 8.7 Hz, 1H), 7.48 (s, 1H), 7.28–7.25 (m, 2H), 7.18–7.15 (m, 3H), 7.02–6.97 (m, 4H), 6.90 (d, *J* = 1.8 Hz, 1H), 6.81 (dd, *J* = 8.6, 2.1 Hz, 1H), 6.47 (d, *J* = 8.9 Hz, 1H), 5.57 (t, *J* = 5.6 Hz, 1H), 5.05 (dd, *J* = 12.7, 5.5 Hz, 1H), 4.28–4.23 (m, 1H), 4.15 (d, *J* = 5.9 Hz, 2H), 3.59–3.57 (m, 1H), 3.52–3.49 (m, 2H), 3.43–3.37 (m, 2H), 3.21 (s, 4H), 3.17–3.14 (m, 1H), 2.91–2.85 (m, 1H), 2.68–2.63 (m, 1H), 2.59–2.56 (m, 5H), 2.41 (d, *J* = 6.9 Hz, 2H), 2.18–2.14 (m, 1H), 2.02–1.99 (m, 1H), 1.90 (d, *J* = 10.4 Hz, 2H), 1.81–1.76 (m, 3H), 1.31–1.27 (m, 2H), 1.13–1.07 (m, 2H).^13^C NMR (151 MHz, DMSO) δ 173.29, 170.63, 168.20, 167.71, 159.72, 157.29, 153.57, 152.35, 150.61, 141.80, 138.78, 134.49, 131.98, 129.96, 128.63, 128.50, 127.16, 126.71, 125.44, 119.59, 115.96, 115.84, 115.73, 109.36, 105.93, 94.46, 61.37, 53.56, 53.39, 52.68, 49.15, 49.10, 48.14, 47.69, 43.94, 35.99, 31.78, 31.47, 30.67, 29.74, 22.73. HRMS (ESI) for C_48_H_52_N_10_O_5_ [M + H]^+^, calcd: 849.4195, found: 849.4200. HPLC purity: 98.55%.

#### 3-Benzyl-1-((1*r*,4*r*)-4-((5-cyanopyridin-2-yl)amino)cyclohexyl)-1-(4-(4-(((3*R*)-1-(2-(2,6-dioxopiperidin-3-yl)-1,3-dioxoisoindolin-5-yl)pyrrolidin-3-yl)methyl)piperazin-1-yl)phenyl)urea (**4f**)

^1^H NMR (600 MHz, DMSO-*d*_6_) δ 11.06 (s, 1H), 8.29 (d, *J* = 2.2 Hz, 1H), 7.64 (d, *J* = 8.4 Hz, 1H), 7.60 (d, *J* = 8.5 Hz, 1H), 7.48 (s, 1H), 7.28–7.25 (m, 2H), 7.18–7.15 (m, 3H), 7.02–6.97 (m, 4H), 6.91 (d, *J* = 1.7 Hz, 1H), 6.82 (dd, *J* = 8.6, 2.0 Hz, 1H), 6.47 (d, *J* = 8.9 Hz, 1H), 5.58–5.56 (m, 1H), 5.05 (dd, *J* = 12.7, 5.5 Hz, 1H), 4.28–4.24 (m, 1H), 4.15 (d, *J* = 5.8 Hz, 2H), 3.60–3.57 (m, 1H), 3.53–3.49 (m, 2H), 3.43–3.39 (m, 2H), 3.21 (s, 4H), 3.17–3.15 (m, 1H), 2.91–2.85 (m, 1H), 2.67–2.64 (m, 1H), 2.59–2.52 (m, 5H), 2.42 (d, *J* = 7.0 Hz, 2H), 2.18–2.15 (m, 1H), 2.02–1.96 (m, 1H), 1.90 (d, *J* = 10.3 Hz, 2H), 1.81–1.76 (m, 3H), 1.31–1.29 (m, 2H), 1.13–1.07 (m, 2H). ^13^C NMR (151 MHz, DMSO) δ 173.29, 170.63, 168.20, 167.71, 159.72, 157.29, 153.58, 152.35, 150.61, 141.80, 138.87, 134.49, 131.98, 129.97, 128.64, 128.50, 127.16, 126.71, 125.45, 119.59, 115.96, 115.84, 115.73, 108.76, 105.93, 94.46, 61.37, 53.57, 53.39, 52.68, 49.15, 48.14, 47.69, 43.94, 35.99, 31.78, 31.46, 30.67, 29.74, 22.56. HRMS (ESI) for C_48_H_52_N_10_O_5_ [M + H]^+^, calcd: 849.4195, found: 849. 4191. HPLC purity: 98.83%.

#### 3-Benzyl-1-((1*r*,4*r*)-4-((5-cyanopyridin-2-yl)amino)cyclohexyl)-1-(4-(4-((1-(2-(2,6-dioxopiperidin-3-yl)-1,3-dioxoisoindolin-5-yl)piperidin-4-yl)methyl)piperazin-1-yl)phenyl)urea (**4g**)

^1^H NMR (600 MHz, DMSO-*d*_6_) δ 11.08 (s, 1H), 8.29 (d, *J* = 2.1 Hz, 1H), 7.65 (d, *J* = 8.5 Hz, 1H), 7.60 (d, *J* = 8.4 Hz, 1H), 7.47 (s, 1H), 7.31 (s, 1H), 7.28–7.25 (m, 2H), 7.23 (d, *J* = 8.6 Hz, 1H), 7.18–7.15 (m, 3H), 7.01–6.97 (m, 4H), 6.51–6.44 (m, 1H), 5.57 (t, *J* = 5.2 Hz, 1H), 5.06 (dd, *J* = 12.8, 5.4 Hz, 1H), 4.26 (t, *J* = 11.8 Hz, 1H), 4.15 (d, *J* = 5.7 Hz, 2H), 4.05 (d, *J* = 12.7 Hz, 2H), 3.48 (s, 1H), 3.19 (s, 4H), 2.97 (t, *J* = 12.0 Hz, 2H), 2.93–2.84 (m, 1H), 2.63–2.53 (m, 2H), 2.52–2.50 (m, 4H), 2.20 (d, *J* = 6.1 Hz, 2H), 2.04–1.99 (m, 1H), 1.90 (d, *J* = 10.3 Hz, 3H), 1.82 (d, *J* = 12.5 Hz, 2H), 1.76 (d, *J* = 10.6 Hz, 2H), 1.35–1.28 (m, 2H), 1.17–1.05 (m, 4H). ^13^C NMR (151 MHz, DMSO) δ 172.81, 170.12, 167.64, 166.97, 159.25, 156.80, 155.01, 153.10, 150.15, 141.33, 138.28, 134.05, 131.50, 128.15, 128.02, 126.69, 126.23, 125.01, 119.12, 117.58, 117.35, 115.34, 108.86, 107.74, 93.99, 63.71, 53.21, 52.91, 48.73, 48.61, 47.70, 47.27, 43.47, 32.49, 31.31, 30.98, 30.20, 29.64, 22.20. HRMS (ESI) for C_49_H_54_N_10_O_5_ [M + H]^+^, calcd: 863.4351, found: 863.4360. HPLC purity: 99.37%.

#### 3-Benzyl-1-((1*r*,4*r*)-4-((5-cyanopyridin-2-yl)amino)cyclohexyl)-1-(4-(3-((4-(2-(2,6-dioxopiperidin-3-yl)-1,3-dioxoisoindolin-5-yl)piperazin-1-yl)methyl)pyrrolidin-1-yl)phenyl)urea (**4i**)

^1^H NMR (600 MHz, DMSO-*d*_6_) δ 11.08 (s, 1H), 8.31–8.27 (m, 1H), 7.68 (d, *J* = 8.5 Hz, 1H), 7.63–7.57 (m, 1H), 7.47 (d, *J* = 7.5 Hz, 1H), 7.34 (d, *J* = 2.2 Hz, 1H), 7.30–7.23 (m, 3H), 7.20–7.13 (m, 3H), 6.98–6.93 (m, 2H), 6.55 (d, *J* = 8.6 Hz, 2H), 6.46 (d, *J* = 8.9 Hz, 1H), 5.44 (t, *J* = 6.2 Hz, 1H), 5.07 (dd, *J* = 12.8, 5.4 Hz, 1H), 4.29–4.24 (m, 1H), 4.15 (d, *J* = 6.1 Hz, 2H), 3.46–3.45 (m, 4H), 3.41–3.36 (m, 3H), 3.28–3.22 (m, 1H), 3.04–3.02 (m, 1H), 2.91–2.85 (m, 1H), 2.64–2.51 (m, 7H), 2.44–2.35 (m, 2H), 2.15–2.07 (m, 1H), 2.04–2.00 (m, 1H), 1.90 (d, *J* = 11.7 Hz, 2H), 1.77–1.71 (m, 3H), 1.33–1.27 (m, 2H), 1.13–1.07 (m, 2H). ^13^C NMR (151 MHz, DMSO) δ 172.83, 170.10, 167.58, 167.00, 159.26, 157.01, 155.25, 153.12, 146.98, 141.31, 138.36, 133.87, 131.60, 128.05, 126.73, 126.28, 124.91, 124.85, 119.14, 118.34, 117.81, 111.82, 109.03, 107.89, 94.00, 61.19, 52.82, 52.60, 51.72, 48.78, 48.69, 46.92, 46.68, 43.50, 35.51, 31.32, 30.99, 30.23, 29.45, 25.14, 22.20. HRMS (ESI) for C_48_H_53_N_10_O_5_ [M + H]^+^, calcd: 849.4195, found: 849.4195. HPLC purity: 99.97%.

#### 3-Benzyl-1-((1*r*,4*r*)-4-((5-cyanopyridin-2-yl)amino)cyclohexyl)-1-(4-(4-((4-(2-(2,6-dioxopiperidin-3-yl)-1,3-dioxoisoindolin-5-yl)piperazin-1-yl)methyl)piperidin-1-yl)phenyl)urea (**4j**).

^1^H NMR (600 MHz, DMSO-*d*_6_) δ 11.08 (s, 1H), 8.29 (d, *J* = 2.4, 1H), 7.68 (d, *J* = 8.5 Hz, 1H), 7.60 (d, *J* = 9.0 Hz, 1H), 7.47 (s, 1H), 7.34 (d, *J* = 2.2 Hz, 1H), 7.28–7.25 (m, 3H), 7.18–7.15 (m, 3H), 6.99–6.96 (m, 4H), 6.47 (d, *J* = 8.9 Hz, 1H), 5.55 (t, *J* = 6.0 Hz, 1H), 5.07 (dd, *J* = 12.8, 5.4 Hz, 1H), 4.28–4.23 (m, 1H), 4.15 (d, *J* = 6.1 Hz, 2H), 3.74 (d, *J* = 11.8 Hz, 2H), 3.44 (s, 4H), 3.42–3.36 (m, 4H), 2.90–2.85 (m, 1H), 2.72–2.68 (m, 2H), 2.61–2.54 (m, 2H), 2.23 (d, *J* = 7.2 Hz, 2H), 2.03–1.99 (m, 1H), 1.90 (d, *J* = 11.6 Hz, 2H), 1.84 (d, *J* = 12.1 Hz, 2H), 1.77–1.72 (m, 3H), 1.31–1.28 (m, 2H), 1.25–1.20 (m, 4H), 1.13–1.07 (m, 2H). ^13^C NMR (151 MHz, DMSO) δ 172.83, 170.10, 167.58, 167.00, 159.26, 156.85, 155.27, 153.11, 150.62, 141.33, 138.50, 133.87, 131.46, 128.04, 127.74, 126.70, 126.25, 124.92, 119.14, 118.32, 117.78, 115.80, 109.05, 107.89, 94.00, 63.74, 52.91, 52.76, 48.78, 48.61, 48.12, 46.96, 43.48, 32.44, 31.32, 30.99, 30.32, 30.21, 22.19. HRMS (ESI) for C_49_H_54_N_10_O_5_ [M + H]^+^, calcd: 863.4351, found: 863.4357. HPLC purity: 98.40%.

#### 3-Benzyl-1-((1*r*,4*r*)-4-((5-cyanopyridin-2-yl)amino)cyclohexyl)-1-(4-(4-(1-(2-(2,6-dioxopiperidin-3-yl)-1,3-dioxoisoindolin-5-yl)azetidin-3-yl)piperazin-1-yl)phenyl)urea (**4k**)

^1^H NMR (500 MHz, DMSO-*d*_6_) δ 11.09 (s, 1H), 8.29 (d, *J* = 2.3 Hz, 1H), 7.66 (d, *J* = 8.2 Hz, 1H), 7.60 (d, *J* = 8.9 Hz, 1H), 7.49 (s, 1H), 7.28–7.25 (m, 2H), 7.18–7.15 (m, 3H), 7.03–6.98 (m, 4H), 6.82 (s, 1H), 6.67 (d, *J* = 8.4 Hz, 1H), 6.46 (d, *J* = 8.9 Hz, 1H), 5.62–5.60 (m, 1H), 5.08–5.04 (m, 1H), 4.28–4.23 (m, 1H), 4.14 (d, *J* = 5.2 Hz, 4H), 3.92 (s, 2H), 3.39 (s, 1H), 3.23 (s, 4H), 2.91–2.84 (m, 1H), 2.64–2.51 (m, 6H), 2.02–2.01 (m, 1H), 1.90 (d, *J* = 11.6 Hz, 2H), 1.77–1.75 (m, 2H), 1.35–1.22 (m, 3H), 1.17–1.05 (m, 2H). ^13^C NMR (126 MHz, DMSO) δ 172.87, 170.17, 167.51, 167.20, 159.26, 156.83, 154.94, 153.14, 150.06, 141.37, 138.32, 133.86, 131.57, 128.32, 128.05, 126.72, 126.27, 124.91, 119.17, 116.92, 115.45, 114.27, 108.91, 104.56, 94.00, 55.06, 54.19, 52.91, 49.16, 48.74, 47.40, 43.49, 31.33, 31.01, 30.23, 29.10, 28.63, 22.23. HRMS (ESI) for C_46_H_48_N_10_O_5_ [M + H]^+^, calcd: 821.3882, found: 821.3884. HPLC purity: 96.54%.

#### 3-Benzyl-1-((1*r*,4*r*)-4-((5-cyanopyridin-2-yl)amino)cyclohexyl)-1-(4-(4-(1-(2-(2,6-dioxopiperidin-3-yl)-1,3-dioxoisoindolin-5-yl)pyrrolidin-3-yl)piperazin-1-yl)phenyl)urea (**4l**)

^1^H NMR (500 MHz, DMSO-*d*_6_) δ 11.08 (s, 1H), 8.29 (d, *J* = 1.8 Hz, 1H), 7.65 (d, *J* = 8.4 Hz, 1H), 7.60 (d, *J* = 8.6 Hz, 1H), 7.48 (d, *J* = 5.9 Hz, 1H), 7.28–7.25 (m, 2H), 7.19–7.15 (m, 3H), 7.01–6.98 (m, 5H), 6.86 (d, *J* = 8.6 Hz, 1H), 6.47 (d, *J* = 8.9 Hz, 1H), 5.59 (t, *J* = 5.7 Hz, 1H), 5.06 (dd, *J* = 12.8, 5.3 Hz, 1H), 4.29–4.23 (m, 1H), 4.15 (d, *J* = 5.7 Hz, 2H), 3.75–3.72 (m, 1H), 3.61–3.58 (m, 1H), 3.50–3.38 (m, 2H), 3.29–3.22 (m, 5H), 3.05–2.98 (m, 1H), 2.91–2.85 (m, 1H), 2.69–2.61 (m, 4H), 2.61–2.52 (m, 2H), 2.31–2.27 (m, 1H), 2.02–2.00 (m, 1H), 1.91–1.89 (m, 3H), 1.77 (d, *J* = 10.5 Hz, 2H), 1.33–1.27 (m, 2H), 1.13–1.06 (m, 2H). ^13^C NMR (126 MHz, DMSO) δ 172.89, 170.22, 167.75, 167.29, 159.27, 156.85, 153.15, 151.81, 150.13, 141.38, 138.44, 134.04, 131.56, 128.29, 128.07, 126.72, 126.27, 124.96, 119.18, 115.80, 115.48, 115.25, 109.11, 105.63, 94.01, 63.55, 52.92, 52.03, 51.35, 48.71, 48.65, 47.64, 47.05, 43.50, 31.34, 31.03, 30.24, 28.89, 22.29. HRMS (ESI) for C_47_H_50_N_10_O_5_ [M+H]^+^, calcd: 835.4038, found: 835.4036. HPLC purity: 97.04%.

#### 3-Benzyl-1-((1*r*,4*r*)-4-((5-cyanopyridin-2-yl)amino)cyclohexyl)-1-(4-(4-(1-(2-(2,6-dioxopiperidin-3-yl)-1,3-dioxoisoindolin-5-yl)piperidin-4-yl)piperazin-1-yl)phenyl)urea (**4m**)

^1^H NMR (600 MHz, DMSO-*d*_6_) δ 11.08 (s, 1H), 8.29 (d, *J* = 2.2 Hz, 1H), 7.66 (d, *J* = 8.5 Hz, 1H), 7.60 (d, *J* = 8.2 Hz, 1H), 7.47 (s, 1H), 7.33 (s, 1H), 7.27–7.25 (m, 3H), 7.18–7.15 (m, 3H), 7.00–6.95 (m, 4H), 6.46 (d, *J* = 8.9 Hz, 1H), 5.58–5.56 (m, 1H), 5.08–5.05 (dd, *J* = 12.8, 5.5 Hz, 1H), 4.28–4.23 (m, 1H), 4.14 (d, *J* = 5.8 Hz, 2H), 4.09 (d, *J* = 12.5 Hz, 2H), 3.48 (s, 1H), 3.17 (s, 4H), 3.01–2.97 (m, 2H), 2.91–2.85 (m, 1H), 2.63 (s, 4H), 2.60–2.50 (m, 4H), 2.03–2.00 (m, 1H), 1.90 (d, *J* = 10.3 Hz, 4H), 1.76 (d, *J* = 10.5 Hz, 2H), 1.51–1.46 (m, 2H), 1.33–1.27 (m, 2H), 1.12–1.06 (m, 2H). ^13^C NMR (151 MHz, DMSO) δ 172.81, 170.11, 167.61, 166.96, 159.24, 156.80, 154.75, 153.09, 150.14, 141.34, 138.41, 134.04, 131.49, 128.14, 128.01, 126.69, 126.22, 125.01, 119.12, 117.70, 117.63, 115.30, 109.07, 107.82, 93.98, 60.53, 52.90, 48.76, 48.74, 48.62, 48.00, 46.61, 43.47, 31.30, 30.98, 30.19, 27.21, 22.19. HRMS (ESI) for C_48_H_52_N_10_O_5_ [M + H]^+^, calcd: 849.4195, found: 849.4199. HPLC purity: 99.08%.

#### 3-Benzyl-1-((1*r*,4*r*)-4-((5-cyanopyridin-2-yl)amino)cyclohexyl)-1-(4-(2-(2-(2,6-dioxopiperidin-3-yl)-1,3-dioxoisoindolin-5-yl)-2,7-diazaspiro[3.5]nonan-7-yl)phenyl)urea (**4n**)

^1^H NMR (600 MHz, DMSO-*d*_6_) δ 11.07 (s, 1H), 8.30 (d, *J* = 2.2 Hz, 1H), 7.65 (d, *J* = 8.3 Hz, 1H), 7.60 (d, *J* = 8.1 Hz, 1H), 7.48 (s, 1H), 7.28–7.26 (m, 2H), 7.19–7.15 (m, 3H), 7.06–7.03–6.99 (m, 4H), 6.80 (d, *J* = 1.8 Hz, 1H), 6.67 (dd, *J* = 8.4, 2.0 Hz, 1H), 6.47 (d, *J* = 8.8 Hz, 1H), 5.56 (t, *J* = 5.6 Hz, 1H), 5.05 (dd, *J* = 12.8, 5.5 Hz, 1H), 4.29–4.23 (m, 1H), 4.15 (d, *J* = 5.8 Hz, 2H), 3.83 (m, 4H), 3.43–3.41 (m, 2H), 3.22 (m, 3H), 2.91–2.85 (m, 1H), 2.59–2.53 (m, 2H), 2.02–1.89 (m, 1H), 1.91–1.89 (m, 6H), 1.77 (d, *J* = 10.1 Hz, 2H), 1.33–1.27 (m, 2H), 1.13–1.07 (m, 2H). ^13^C NMR (151 MHz, DMSO) δ 172.83, 170.12, 167.53, 167.21, 159.26, 156.83, 155.20, 153.12, 150.21, 141.33, 138.35, 133.86, 131.53, 128.04, 126.71, 126.26, 124.87, 122.51, 119.14, 116.69, 115.95, 114.18, 108.92, 104.42, 94.00, 64.11, 60.78, 52.92, 48.73, 48.63, 45.32, 43.49, 34.65, 34.21, 31.32, 30.99, 30.21, 22.23. HRMS (ESI) for C_46_H_47_N_9_O_5_ [M + H]^+^, calcd: 806.3773, found: 806.3767. HPLC purity: 95.07%.

#### 3-Benzyl-1-((1*r*,4*r*)-4-((5-cyanopyridin-2-yl)amino)cyclohexyl)-1-(4-(2-(2-(2,6-dioxopiperidin-3-yl)-1,3-dioxoisoindolin-5-yl)-2,8-diazaspiro[4.5]decan-8-yl)phenyl)urea (**4o**)

^1^H NMR (600 MHz, DMSO-*d*_6_) δ 11.06 (s, 1H), 8.30 (d, *J* = 2.2 Hz, 1H), 7.65 (d, *J* = 8.4 Hz, 1H), 7.60 (d, *J* = 8.2 Hz, 1H), 7.48–7.45 (m, 1H), 7.28–7.26 (m, 2H), 7.19–7.15 (m, 3H), 7.02–6.99 (m, 4H), 6.96 (s, 1H), 6.83 (dd, *J* = 8.6, 1.6 Hz, 1H), 6.47 (d, *J* = 8.9 Hz, 1H), 5.52 (t, *J* = 5.6 Hz, 1H), 5.07–5.04 (m, 1H), 4.29–4.24 (m, 1H), 4.15 (d, *J* = 5.9 Hz, 2H), 3.53–3.50 (m, 2H), 3.37 (m, 4H), 3.24–3.21 (m, 1H), 2.91–2.85 (m, 1H), 2.61–2.52 (m, 4H), 2.02–1.98 (m, 1H), 1.97–1.95 (m, 2H), 1.91 (d, *J* = 10.7 Hz, 2H), 1.77 (d, *J* = 10.3 Hz, 2H), 1.73–1.66 (m, 4H), 1.33–1.27 (m, 2H), 1.13–1.07 (m, 2H). ^13^C NMR (151 MHz, DMSO) δ 172.83, 170.16, 167.74, 167.26, 159.25, 156.82, 153.12, 152.06, 150.42, 141.28, 138.33, 134.02, 131.48, 128.05, 127.83, 126.70, 126.27, 124.94, 119.13, 115.80, 115.54, 115.22, 108.89, 105.58, 94.00, 57.28, 52.90, 48.69, 48.62, 46.32, 45.44, 43.49, 35.02, 34.06, 31.31, 31.00, 30.22, 29.02, 22.26. HRMS (ESI) for C_47_H_49_N_9_O_5_ [M + H]^+^, calcd: 820.3929, found: 820.3933. HPLC purity: 97.24%.

#### 3-Benzyl-1-((1*r*,4*r*)-4-((5-cyanopyridin-2-yl)amino)cyclohexyl)-1-(4-(9-(2-(2,6-dioxopiperidin-3-yl)-1,3-dioxoisoindolin-5-yl)-3,9-diazaspiro[5.5]undecan-3-yl)phenyl)urea (**4p**)

^1^H NMR (600 MHz, DMSO-*d*_6_) δ 11.07 (s, 1H), 8.29 (d, *J* = 2.1 Hz, 1H), 7.66 (d, *J* = 8.5 Hz, 1H), 7.60 (d, *J* = 8.7 Hz, 1H), 7.48 (s, 1H), 7.32 (s, 1H), 7.28–7.23 (m, 3H), 7.18–7.15 (m, 3H), 6.99 (s, 4H), 6.50–6.43 (m, 1H), 5.54–5.52 (m, 1H), 5.06 (dd, *J* = 12.8, 5.5 Hz, 1H), 4.28–4.24 (m, 1H), 4.15 (d, *J* = 5.8 Hz, 2H), 3.51 (s, 6H), 3.22 (s, 4H), 2.91–2.85 (m, 1H), 2.60–2.57 (m, 1H), 2.03–1.99 (m, 1H), 1.91 (d, *J* = 10.6 Hz, 2H), 1.77 (d, *J* = 10.4 Hz, 2H), 1.63–1.59 (m, 8H), 1.33–1.28 (m, 2H), 1.14–1.07 (m, 2H). ^13^C NMR (151 MHz, DMSO) δ 172.83, 170.14, 167.67, 167.00, 159.25, 156.84, 154.97, 153.11, 150.48, 141.31, 138.24, 134.04, 131.46, 128.05, 127.66, 126.70, 126.26, 125.00, 119.13, 117.35, 117.29, 115.47, 108.99, 107.50, 94.00, 69.79, 52.90, 48.74, 48.63, 43.48, 42.89, 34.82, 34.12, 31.31, 30.99, 30.21, 29.14, 22.21. HRMS (ESI) for C_48_H_51_N_9_O_5_ [M + H]^+^, calcd: 834.4086, found: 834.4073. HPLC purity: 97.46%.

#### 3-Benzyl-1-((1*r*,4*r*)-4-((5-cyanopyridin-2-yl)amino)cyclohexyl)-1-(4-(4-((1-(2-(2,6-dioxopiperidin-3-yl)-6-fluoro-1,3-dioxoisoindolin-5-yl)piperidin-4-yl)methyl)piperazin-1-yl)phenyl)urea (**4g-F**)

^1^H NMR (600 MHz, DMSO-*d*_6_) δ 11.11 (s, 1H), 8.31–8.28 (m, 1H), 7.70 (d, *J* = 11.4 Hz, 1H), 7.60 (dd, *J* = 9.3, 2.3 Hz, 1H), 7.47 (d, *J* = 7.0 Hz, 1H), 7.44 (d, *J* = 7.4 Hz, 1H), 7.28–7.25 (m, 2H), 7.19–7.14 (m, 3H), 7.01–6.97 (m, 4H), 6.47 (d, *J* = 8.9 Hz, 1H), 5.57 (t, *J* = 6.3 Hz, 1H), 5.10 (dd, *J* = 12.9, 5.4 Hz, 1H), 4.30–4.24 (m, 1H), 4.15 (d, *J* = 6.0 Hz, 2H), 3.61 (d, *J* = 11.7 Hz, 2H), 3.55–3.41 (m, 1H), 3.24–3.14 (m, 4H), 2.94–2.84 (m, 3H), 2.63–2.50 (m, 6H), 2.24 (d, *J* = 7.0 Hz, 2H), 2.05–2.01 (m, 1H), 1.94–1.88 (m, 2H), 1.87–1.82 (m, 2H), 1.81–1.72 (m, 3H), 1.35–1.23 (m, 4H), 1.14–1.06 (m, 2H). ^13^C NMR (151 MHz, DMSO) δ 172.74, 169.90, 166.70, 166.20, 159.23, 157.24 (d, *J* = 253.4 Hz), 156.79, 153.07, 150.13, 145.87 (d, *J* = 8.9 Hz), 141.32, 138.29, 131.48, 128.78 (d, *J* = 2.0 Hz), 128.13, 128.00, 126.67, 126.21, 122.78 (d, *J* = 9.7 Hz), 119.10, 115.32, 113.66 (d, *J* = 4.7 Hz), 111.87 (d, *J* = 25.2 Hz), 108.94, 93.97, 63.77, 53.23, 52.90, 49.95, 49.02, 48.59, 47.68, 43.46, 32.24, 31.29, 30.94, 30.27, 30.18, 22.07. HRMS (ESI) for C_49_H_53_FN_10_O_5_ [M + H]^+^, calcd: 881.4257, found: 881.4257. HPLC purity: 98.10%.

#### 3-Benzyl-1-((1*r*,4*r*)-4-((5-cyanopyridin-2-yl)amino)cyclohexyl)-1-(4-(4-(1-(2-(2,6-dioxopiperidin-3-yl)-6-fluoro-1,3-dioxoisoindolin-5-yl)piperidin-4-yl)piperazin-1-yl)phenyl)urea (**ZLC491**)

^1^H NMR (600 MHz, DMSO-*d*_6_) δ 11.11 (s, 1H), 8.29 (d, *J* = 2.2 Hz, 1H), 7.71 (d, *J* = 11.4 Hz, 1H), 7.59 (d, *J* = 8.4 Hz, 1H), 7.45 (m, 2H), 7.28–7.25 (m, 2H), 7.18–7.15 (m, 3H), 7.01–6.96 (m, 4H), 6.46 (d, *J* = 8.9 Hz, 1H), 5.57 (m, 1H), 5.11 (dd, *J* = 12.9, 5.4 Hz, 1H), 4.28–4.26 (m, 1H), 4.15 (d, *J* = 5.7 Hz, 2H), 3.67 (d, *J* = 11.5 Hz, 2H), 3.49 (s, 1H), 3.19 (s, 4H), 2.93–2.86 (m, 3H), 2.66 (s, 4H), 2.61–2.58 (m, 1H), 2.54–2.52 (d, *J* = 13.2 Hz, 1H), 2.49–2.44 (d, *J* = 15.7 Hz, 1H), 2.04–2.02 (m, 1H), 1.93–1.90 (m, 4H), 1.76 (d, *J* = 10.4 Hz, 2H), 1.62–1.57 (m, 2H), 1.33–1.27 (m, 2H), 1.13–1.07 (d, 2H). ^19^F NMR (376 MHz, DMSO-*d*_6_) δ −111.98. ^13^C NMR (151 MHz, DMSO-*d*_6_) δ 172.76, 169.91, 166.69, 166.21, 159.25, 157.25 (d, *J* = 253.2 Hz), 156.81, 153.09, 150.15, 145.50 (d, *J* = 8.9 Hz), 141.34, 138.29, 131.50, 128.78 (d, *J* = 1.8 Hz), 128.14, 128.01, 126.69, 126.22, 123.00(d, *J* = 9.7 Hz), 119.11, 115.29, 113.74 (d, *J* = 4.4 Hz), 111.92 (d, *J* = 25.4 Hz), 108.98, 93.99, 60.39, 52.92, 49.33, 49.05, 48.77, 48.61, 48.02, 43.48, 31.31, 30.96, 30.20, 27.88, 22.09. HRMS (ESI) for C_48_H_51_FN_10_O_5_ [M+H]^+^, calcd: 867.4101, found: 867.4104. HPLC purity: 99.43%.

#### 3-Benzyl-1-((1*r*,4*r*)-4-((5-cyanopyridin-2-yl)amino)cyclohexyl)-1-(4-(4-(1-(6-fluoro-2-(1-methyl-2,6-dioxopiperidin-3-yl)-1,3-dioxoisoindolin-5-yl)piperidin-4-yl)piperazin-1-yl)phenyl)urea (**ZLC491N**)

^1^H NMR (600 MHz, DMSO-*d*_6_) δ 8.29 (dd, *J* = 2.4, 0.7 Hz, 1H), 7.71 (d, *J* = 11.4 Hz, 1H), 7.63–7.57 (m, 1H), 7.50–7.43 (m, 2H), 7.28–7.25 (m, 2H), 7.19–7.14 (m, 3H), 7.01–6.97 (m, 4H), 6.47 (d, *J* = 8.9 Hz, 1H), 5.58 (t, *J* = 6.1 Hz, 1H), 5.17 (dd, *J* = 13.1, 5.4 Hz, 1H), 4.29–4.23 (m, 1H), 4.15 (d, *J* = 6.1 Hz, 2H), 3.68 (d, *J* = 11.9 Hz, 2H), 3.49 (s, 1H), 3.20–3.18 (m, 4H), 3.01 (s, 3H), 2.98–2.88 (m, 3H), 2.78–2.74 (m, 1H), 2.67 (s, 4H), 2.58–2.51 (m, 1H), 2.49–2.42 (m, 1H), 2.07–2.03 (m, 1H), 1.94–1.89 (m, 4H), 1.81–1.73 (m, 2H), 1.66–1.55 (m, 2H), 1.33–1.27 (m, 2H), 1.16–1.05 (m, 2H). ^13^C NMR (151 MHz, DMSO) δ 171.74, 169.68, 166.68, 166.19, 159.24, 157.26 (d, *J* = 253.2 Hz), 156.80, 153.09, 150.15, 145.52 (d, *J* = 8.9 Hz), 141.34, 138.37, 131.50, 128.77 (d, *J* = 2.0 Hz), 128.15, 128.01, 126.69, 126.22, 122.98 (d, *J* = 9.7 Hz), 119.11, 115.30, 113.77 (d, *J* = 3.9 Hz), 111.94 (d, *J* = 25.2 Hz), 108.70, 93.98, 60.39, 52.91, 49.62, 49.33, 48.76, 48.61, 48.02, 43.47, 31.30, 31.11, 30.20, 27.87, 26.62, 21.27. HRMS (ESI) for C_49_H_53_FN_10_O_5_ [M + H]^+^, calcd: 881.4257, found: 881.4257. HPLC purity: 99.14%.

#### 3-Benzyl-1-((1*r*,4*r*)-4-((5-cyanopyridin-2-yl)amino)cyclohexyl)-1-(4-(3-((4-(2-(2,6-dioxopiperidin-3-yl)-1,3-dioxoisoindolin-5-yl)piperazin-1-yl)methyl)azetidin-1-yl)phenyl)urea (**4h**)

To a stirred solution of compound **14a** in methanol (2 mL) was added Pd/C (10% Pd, 10 mg). The resulting suspension was then backfilled with hydrogen (3 cycles) and stirred. Until completed as indicated by TLC, the reaction was filtrated and evaporated. The residue was used directly in the next step without further purification. To a solution of the residue in DMF were added DIPEA (3 eq) and compound **7a** (1 eq). The resulting solution was heated at 100 °C for 2 h under stirring. Upon cooling, the solvent was removed in vacuo. The residue was then purified by silica gel column chromatography to yield target compound **4h**. ^1^H NMR (600 MHz, DMSO-*d*_6_) δ 11.08 (s, 1H), 8.29 (d, *J* = 2.2 Hz, 1H), 7.68 (d, *J* = 8.5 Hz, 1H), 7.60 (d, *J* = 8.9 Hz, 1H), 7.47 (s, 1H), 7.36–7.34 (m, 1H), 7.28–7.25 (m, 3H), 7.18–7.14 (m, 3H), 6.95 (d, *J* = 8.5 Hz, 2H), 6.47–6.45 (m, 3H), 5.48–5.47 (m, 1H), 5.07 (dd, *J* = 12.8, 5.4 Hz, 1H), 4.27–4.23 (m, 1H), 4.14 (d, *J* = 5.9 Hz, 2H), 3.97 (m, 2H), 3.54 – 3.50 (m, 2H), 3.45 (s, 4H), 3.00–2.95 (m, 1H), 2.90–2.87 (m, 1H) 2.64 (d, *J* = 7.3 Hz, 2H), 2.61–2.57 (m, 2H), 2.54–2.52 (m, 5H), 2.03–1.99 (m, 1H), 1.90 (d, *J* = 10.4 Hz, 2H), 1.75 (d, *J* = 11.6 Hz, 2H), 1.31–1.27 (m, 2H), 1.11–1.05 (m, 2H). ^13^C NMR (151 MHz, DMSO) δ 172.82, 170.09, 167.57, 166.99, 159.25, 156.92, 155.24, 153.11, 150.90, 141.29, 138.34, 133.87, 131.44, 128.05, 126.70, 126.49, 126.26, 124.91, 119.13, 118.35, 117.85, 111.48, 109.02, 107.93, 94.00, 61.93, 56.11, 52.87, 52.34, 48.78, 48.62, 46.88, 43.48, 31.30, 30.98, 30.20, 29.03, 27.22, 22.18. HRMS (ESI) for C_47_H_50_N_10_O_5_ [M + H]^+^, calcd: 835.4038, found: 835.4045. HPLC purity: 98.54%.

### Cell Culture

TNBC cell lines MDA-MB-231, MDA-MB-436 and HCC38, human embryonic kidney cell line HEK 293 and human embryonic lung cell line MRC-5 were purchased from American Type Culture Collection (ATCC). Human mammary epithelial cell line MCF 10A was obtained from Chinese Academy of Sciences National Collection of Authenticated Cell Cultures. MDA-MB-231 was maintained in Leibovitz’ s L-15 medium (Gibco) supplemented with 10% fetal bovine serum (FBS; Excell Bio) and 1% penicillin/streptomycin (P/S; Gibco) at 37 °C in a humidified chamber of 100% air atmosphere. MDA-MB-436 and HCC38 were developed in Dulbecco’s modified Eagle medium (DMEM; Gibco) and RPMI-1640 medium (Gibco), respectively, both supplemented with 10% FBS (Excell Bio) and 1% P/S (Gibco), using a 37 °C constant temperature incubator with 5% CO_2_. HEK 293 and MRC-5 were cultured in the same condition with Eagle’s minimum essential medium (EMEM; Wisent). MCF 10A was grown in Mammary Epithelial Cell Growth Medium BulletKit (Lonza) at 37 °C in a humidified chamber with 5% CO_2_.

### Western Blotting

Generally, the tested cells were washed by ice-cold PBS buffer (Gibco) and lysed with an SDS lysis buffer (62.5 mM Tris, 2% w/v SDS, 10 glycerol, 50 mM DTT, 0.01% w/v bromophenol blue) on ice, after which the protein lysate samples were collected to 1.5 mL microcentrifuge tubes and treated with ultrasonic for 10 s, following with a 100 °C incubation for 10 min and a 4 °C centrifugation of 12000 rpm for 10 min. An equal amount of protein was loaded to 4–8% Bis-Tris gels and underwent an SDS-PAGE electrophoresis at 55V for 30 min and 95V for 90 min. Proteins on gels were then transferred to a polyvinylidene difluoride (PVDF) membrane (Bio-Rad) with a wet electro transfer system (Bio-Rad). The PVDF membrane was cut into bands and incubated in a blocking reagent (5% defatted milk powder dissolved in TBST) at room temperature for 1–2 h. Incubate membrane and primary antibody (at the appropriate dilution and diluent as recommended in the product webpage) at 4 °C for 12–14 h, followed with three times washes by TBST. The washed membranes were incubated in a secondary antibody solution (HRP-linked antirabbit IgG or antimouse IgG (CST), 1:2000 diluted into TBST) at room temperature for 2 h. Membranes were photographed in Amersham Imager 800 system using StarSignal plus regent (GenStar) or Omni-ECL reagent (EpiZyme). Compounds MG132, MLN4924, Bafilomycin A1 and pomalidomide were purchased from MedChemExpress (MCE) LLC.

### TMT-Labeled Quantitative Proteomics Assay

#### Sample Preparation

MDA-MB-231 cells were seeded at 8 × 10^5^ cells in 6 cm dishes 24 h before treating with 2 μM **ZLC491** or DMSO in quadruplicate or triplicate for 8 h. Cell lysates were prepared by washing cells twice with cold PBS and adding 180 μL lysis buffer (10 mM HEPES, pH 7.0, 1% (w/v) SDS, 2 mM MgCl·6H_2_O supplemented with 0.1% BeyoZonase (Beyotime)) per dish. Samples were quantified using a micro-BCA protein assay kit (Thermo Fisher Scientific) and 600 μg of each sample was processed and digested using the filter aided sample preparation (FASP) method followed by alkylation with iodoacetamide and digestion with trypsin. The samples were then desalted using Oasis HLB C18 SPE cartridge column (Waters, WAT094225). The peptides were lyophilized and reconstituted, then 25 μg peptides of each sample were labeled with TMT 11-plex Isobaric Label Reagent Set (Thermo Fisher Scientific) as the manufacturer’s instructions. After labeling, the peptides from the 11 samples were pooled together. The pooled TMT 11-plex sample was fractionated using high pH reverse-phase chromatography on an XBridge peptide BEH column (130 Å, 3.5 μm, 2.1 × 150 mm, Waters) on an Ultimate 3000 HPLC system (Thermo Scientific/Dionex). Buffer A (10 mM ammonium formate in water, pH 9.0) and B (10 mM ammonium formate in 90% acetonitrile, pH 9.0) were used over a linear gradient of 5% to 60% buffer B over 60 min at a flow rate of 200 μL/min. 48 fractions were collected before concatenation into 12 fractions based on the UV signal of each fraction. All the fractions were dried in a concentrator and were reconstituted in 0.2% formic acid for MS analysis.

#### LC–MS/MS Analysis

The fractions were analyzed sequentially on an Orbitrap Fusion Lumos Mass Spectrometer (Thermo Scientific) coupled to a NanoLC-1200 UHPLC system (Thermo Scientific) with C18 capillary column. LC separation was using gradient of 6 to 28% acetonitrile in 0.2% formic acid. Separation flow rate was setup as 250 nL/min. MS3-TMT mass spectrometry methods were modified from the methods reported by Steven Gygi group.^28^ The mass spectrometer was operated in data dependent mode and the scan sequence began with an orbitrap MS1 spectrum at resolution 120,000, AGC target 5E5, MaxIT 50 ms. The 20 most intense precursor ions were selected for MS2/MS3 analysis. MS2 analysis consisted of CID and ion trap analysis. Following acquisition of MS2 spectrum, the MS3 spectrum was captured with multinotches. MS3 precursor were fragmented by HCD and analyzed with Orbitrap at NCE 65, AGC 1E5, resolution 60000.

#### Peptide and Protein Identification

The raw MS data files for all 11 fractions were searched against the Uniprot-Human-Canonical database by Proteome Discoverer 2.0 for protein identification and TMT reporter ion quantitation. The searches parameters were set as following, enzyme used trypsin/P; maximum number of missed cleavages equal to two; precursor mass tolerance equal to 20 ppm; Ion trap fragment mass tolerance was set to 0.6 Da; variable modifications: oxidation (M), acetyl (N-term), fixed modifications: carbamidomethyl (C). The data was filtered by applying a 1% false discovery rate.

### cDNA Preparation and Real-Time Quantitative PCR

RNA utilized for RT-qPCR was extracted as outlined below. MDA-MB-231 cells were incubated in media containing **ZLC491** at the indicated concentrations or DMSO for 12 h. Total RNA from biological replicates (equivalent to 1 million cells per replicate) was subsequently isolated using Invitrogen TRIzol Reagent (Thermofisher Scientific) following the manufacturer’s instructions and resuspended in 30 μL of nuclease-free water (Takara). 2 μg of purified RNA was reverse transcribed using PrimeScript RT reagent Kit with gDNA Eraser (Takara) to cDNA according to the manufacturer’s protocol. RT-qPCR was carried out on the QuantStudio Real-Time PCR System (Applied Biosystems) using the corresponding primer pairs shown in Table S2 and PowerUp SYBR Green Master Mix (Applied Biosystems) according to the manufacturer’s protocol. All experiments were performed in biological duplicate. Each individual biological sample was qPCR-amplified in technical quadruplicate. Error bars are ±SD. Expression was normalized to GAPDH, and fold change in expression was calculated relative to the indicated conditions using ΔΔCt method.

### Cell Proliferation Assays

Tested cells were plated in 96-well or 384-well plates and incubated at 37 °C with or without 5% CO_2_. After overnight incubation, serial dilutions of compounds were added to the plate. Cell proliferation was measured 5 days after compound treatment using Cell Counting Kit-8 (Selleck) according to the manufacturer’s instructions. The absorbance signal at OD450 and 650 was detected by EnVision plate reader (PerkinElmer), and the IC_50_ values were determined by nonlinear regression and a four-parameter algorithm (GraphPad Prism).

### Drug Combination Analysis

Cisplatin and Olaparib used in the combination assay were purchased from Bide Pharmatech Co., Ltd. To assess combination index (CI), different dose combinations of the initial treatment concentration of each drug (cisplatin and olaparib equal to 25 μM, **ZLC491** either to 0.3 μM, 1.5 μM or 7.5 μM) was used to generate 10-point 1:3 dilution concentration–response curves. Loewe additivity is a dose–effect model, which states that additivity occurs in a two-drug combination if the sum of the ratios of the dose vs the median-effect for each individual drug is 1. In this model, CI scores estimate the interaction between the two drugs. If CI < 1, the drugs have a synergistic effect and if CI > 1, the drugs have an antagonistic effect. CI = 1 means the drugs have an additive effect. Chou and Talalay^29^ showed that Loewe equations are valid for enzyme inhibitors with similar mechanisms of action – either competitive or noncompetitive toward the substrate. The Chou–Talalay combination Index coefficients were analysis and computed based on the Chou–Talalay Median Effect model as implemented in CompuSyn v1.0 (http://www.combosyn.com).

### *In Vivo* Studies

The pharmacokinetic investigation was taken by Shanghai Medicilon Inc. (Project Code: 26048-23001-NG). Sprague–Dawley male rats (Specific pathogen Free (SPF), provided by Zhejiang Vital River Laboratory Animal Technology Co. Ltd.) were dosed with **ZLC491** solution formulation (5% DMSO, 10% Solutol, 85% normal saline, 2.5 mg/kg for intravenous dose, 10 mg/kg for oral dose) or compound **4m** solution formulation (5% DMSO, 10% Solutol, 85% (20%HP-β-CD), 2.5 mg/kg for intravenous dose, 10 mg/kg for oral dose). Blood samples were collected at 0.083, 0.25, 0.5, 1, 2, 4, 8, and 24 h in intravenous administration, 0.25, 0.5, 1, 2, 4, 6, 8, and 24 h in oral administration. The blood samples were collected from sets of three rats at each time point in labeled microcentrifuge tubes containing heparin sodium as an anticoagulant. Plasma samples were separated by centrifugation (2–8 °C, 6800*g* for 6 min) within 1 h and stored below −80 °C until bioanalysis. All samples were processed for analysis by precipitation using acetonitrile and analyzed with a partially validated LC/MS/MS method. Pharmacokinetic parameters were calculated using the noncompartmental analysis tool of WinNonlin Enterprise software.

The pharmacodynamics experiments were performed under an approved animal protocol by the Institutional Animal Care & Use Committee of the University of Michigan. Six- to eight-week-old CB17SCID female (Charles River Laboratory) mice were in a regular SPF housing room prior to cell injection. Briefly, 5 × 10^6^ cells of MDA-MB-231 were injected orthotopically into the mammary fat pad of CB17SCID mice. After tumor size reached approximately 100–200 mm^3^, animals were subjected to drug treatment by oral gavage. Vehicle consisted of 20% PEG400, 6% Cremophor EL, and 74% PBS solution. Tumors were collected at the end of the experiment for Western blot analysis.

### Histology Analysis

H&E staining of formalin-fixed paraffin embedded (FFPE) tissue sections was performed using Leica Autostainer XL as per manufacturer’s protocol. Apoptosis was evaluated by Terminal dUTP Nick End Labeling (TUNEL) assay performed on Ventana Benchmark Ultra staining platform using an In Situ Cell Death Detection Kit (POD # 11684817910, Roche Applied Sciences) as per manufacturer’s protocol. Briefly, FFPE sections were deparaffinized and rehydrated in graded ethanol followed by permeabilization with Proteinase K working solution at 37 °C. Then, the sections were incubated with TUNEL reaction mixture. Following stringent washing, signal was developed using converter-POD solution and DAB working solution. Cells which were intensely stained for DAB pigment were considered to be apoptotic and they were quantified as numbers per 10 high power field (hpf, 400× magnification).

## Supporting Information Available

The Western blot results for preliminary screening; transcription level of CDK12/13 genes after treatment with **ZLC491**; immunoblotting results of CCNK in MDA-MB-231 cells treated with **ZLC491**; cell antiproliferative activity of **ZLC491N** in multiple TNBC cells and noncancerous cells; results of replications in the DC_50_ determination of **ZLC491** in MDA-MB-231 cells; primer sequences for RT-qPCR; synthesis of intermediates **13** and **7c**; ^1^H NMR, ^13^C NMR, HRMS, and HPLC traces for all the synthesized degraders; ^19^F NMR of compound **ZLC491** (PDF)
molecular formula strings (CSV)
**ZLC491** proteomics (XLSX)

## Abbreviations

TNBC: triple-negative breast cancer
DDR: DNA damage response
CCNK: cyclin K
CDK12/13: CDK12 and CDK13
PK: pharmacokinetics
HATU: 2-(7-azabenzotriazol-1-yl)-*N*′,*N*′,*N*′-tetramethyluronium hexafluorophosphate
DIPEA: *N*,*N*-diisopropylethylamine
DMF: *N*,*N*-dimethylformamide
TFA: trifluoroacetic acid
CH_2_Cl_2_: dichloromethane
K_2_CO_3_: potassium carbonate
Pd_2_(dba)_3_: tris(dibenzylideneacetone) dipalladium
Xantphos: 4,5-bis(diphenylphosphino)-9,9-dimethylxanthene
*tert*-BuONa: sodium *tert*-butoxide
Cs_2_CO_3_: cesium carbonate
NaBH(OAc)_3_: sodium triacetoxyborohydride
Pd(pph_3_)_2_Cl_2_: bis(triphenylphosphine)palladium chloride
DC_50_: half degradation concentration
TMT: tandem mass tag
NAE: NEDD8-activating enzyme
RT-qPCR: real-time quantitative polymerase chain reaction
PARP: poly ADP-ribose polymerase
CI: combination index
T_1/2_: terminal half-life
C_max_: maximum drug plasma concentration
AUC: area under the drug concentration–time curve
CL: plasma clearance rate
F: oral bioavailability
i.v.: intravenous administration
p.o.: oral administration
TLC: thin-layer chromatography
TUNEL: terminal dUTP nick end labeling
IHC: immunohistochemistry
