# Supplementary material for: Discovery of ZLC491 as a Potent, Selective, and Orally Bioavailable CDK12/13 PROTAC Degrader
Source: J Med Chem. 2024 Oct 10;67(20):18247–64. doi: 10.1021/acs.jmedchem.4c01596 (PMC11513923; doi:10.1021/acs.jmedchem.4c01596)

Supporting Information

Discovery of ZLC491 as a Potent, Selective and Orally Bioavailable CDK12/13 PROTAC Degrader

Licheng Zhou[α, β, #], Kaijie Zhou[β, δ, #], Yu Chang [Φ, ξ, #], Jianzhang Yang[α], Bohai Fan[β], Yuhan Su[β], Zilu Li[β], Rahul Mannan[Φ, ξ], Somnath Mahapatra[Φ, ξ], Ming Ding[Ψ], Fengtao Zhou[α], Weixue Huang[β], Xiaomei Ren[β], Jian Xu[σ], George Xiaoju Wang[Φ, g, ξ], Jinwei Zhang[β], Zhen Wang[β,*], Arul M. Chinnaiyan[Φ, χ, ξ, g, ρ,*] and Ke Ding[α, β,*]

[α] International Cooperative Laboratory of Traditional Chinese Medicine Modernization and Innovative Drug Discovery of Chinese Ministry of Education (MOE), Guangzhou City Key Laboratory of Precision Chemical Drug Development, College of Pharmacy, Jinan University, 855 Xingye Avenue East, Guangzhou 511400, China

[β] State Key Laboratory of Chemical Biology, Shanghai Institute of Organic Chemistry, Chinese Academy of Sciences, #345 Lingling Rd., Shanghai 200032, China

[δ] University of Chinese Academy of Sciences, No. 1 Yanxihu Road, Huairou District, Beijing 101408, China

[Ψ]School of Life Science and Technology, China Pharmaceutical University, 639 Longmian Avenue, Nanjing 211198, China

[Φ] Michigan Center for Translational Pathology, University of Michigan, Ann Arbor, Michigan 48109, USA

[χ] Howard Hughes Medical Institute, University of Michigan, Ann Arbor, MI 48109, USA

[ξ] Department of Pathology, University of Michigan, Ann Arbor, MI 48109, USA

[g] Rogel Cancer Center, University of Michigan, Ann Arbor, MI 48109, USA

[ρ] Department of Urology, University of Michigan, Ann Arbor, MI 48109, USA

[σ] Livzon Research Institute, Livzon Pharmaceutical Group Inc., No. 38, Chuangye North Road, Jinwan District, Zhuhai 519000, China

[#]L. Z., K. Z. and Y. C. contributed equally to this work.

[*]Email: wangz@sioc.ac.cn (Z.W.); arul@med.umich.edu (A.M.C.);

dingk@sioc.ac.cn, Tel: +86-21-5492 5100 (K.D.)

Table of Contents

1. Immunoblotting results of CDK12/13 in MDA-MB-231 cells treated with all degraders……………………..…………………...……………......................……S4
2. Transcription level of CDK12/13 genes in MDA-MB-231 cells upon the treatment with vehicle or **ZLC491**…………………………………………………………S5
3. Immunoblotting results of CCNK in MDA-MB-231 cells treated with **ZLC491**……………………………………………………………………...…..S6
4. Cell antiproliferative activity of **ZLC491N** in multiple TNBC cells and noncancerous cells…………………………………...………………..………S6
5. Results of replications in the DC_{50} determination of **ZLC491** in MDA-MB-231 cells…………………………..………………………….……………………..S7
6. Primers used in RT-qPCR…………………………………………….……….…S8
7. Synthesis of compounds **13** and **7c**………………………..….…….……...S9-S10
8. ^{1}H NMR, ^{13}C NMR, HRMS and HPLC spectra of all the final compounds………………………………………………………………....S11-S48
9. ^{19}F NMR of **ZLC491**…………………………….…………………………S49

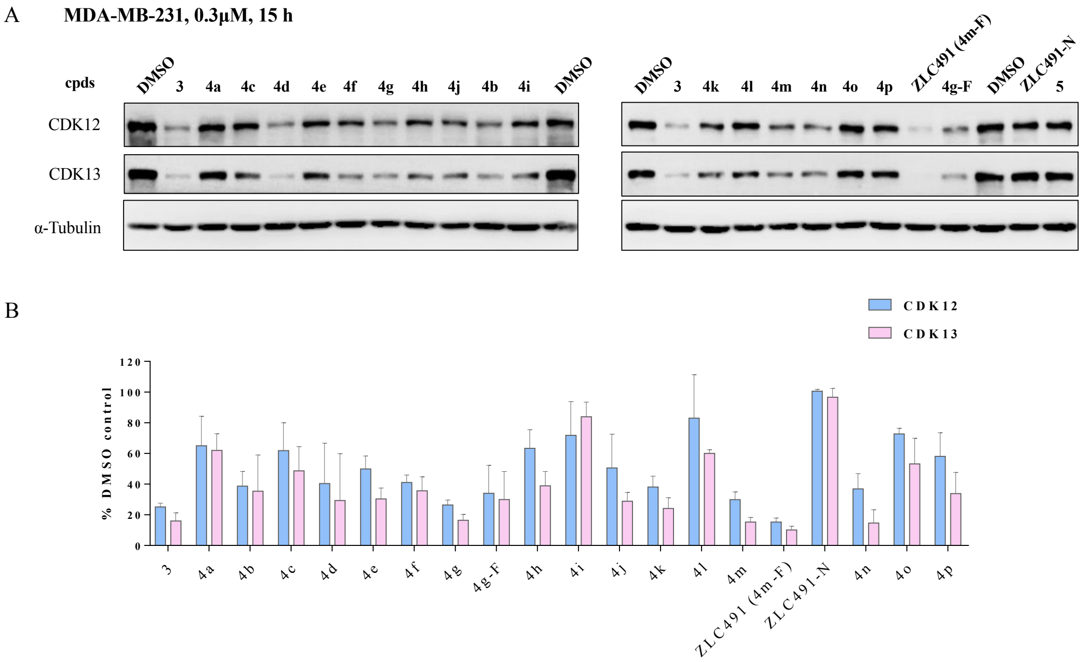

Figure S1. (A) Immunoblotting results in the preliminary detection of CDK12/13 degradation activities of synthesized degraders at 0.3 μM for 15 h in MDA-MB-231 cells. Experiments were performed in biological triplicate. (B) Protein levels in immunoblotting assay results were quantified using Image J and normalized to corresponding α-Tubulin, then plotted using GraphPad Prism 8.0. Data were plotted as mean ± SD.

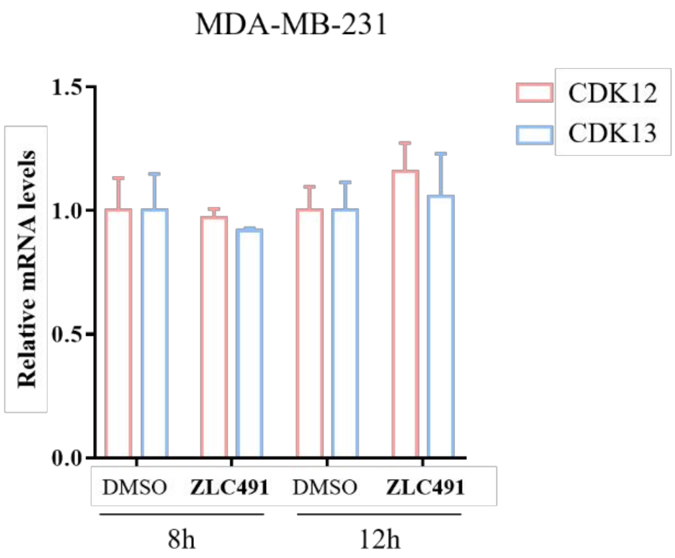

Figure S2. Transcription level of CDK12/13 genes in MDA-MB-231 cells by RT-qPCR upon the treatment with vehicle or 60 nM **ZLC491** for 8 or 12 h. (Four replicates per treatment, normalized to GAPDH and DMSO).

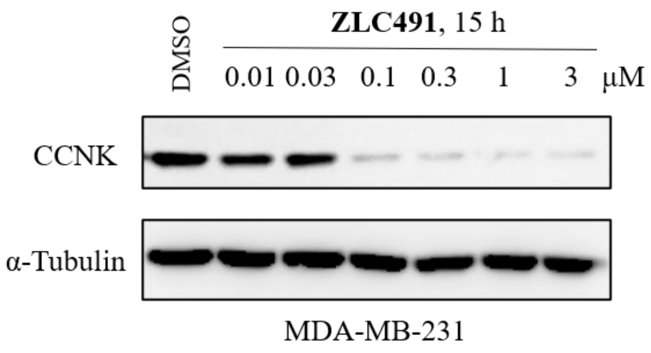

Figure S3. Immunoblotting of CCNK in MDA-MB-231 cells treated with increasing concentrations of **ZLC491** for 15 h. The result showed that **ZLC491** dose-dependently degraded CCNK in cells.

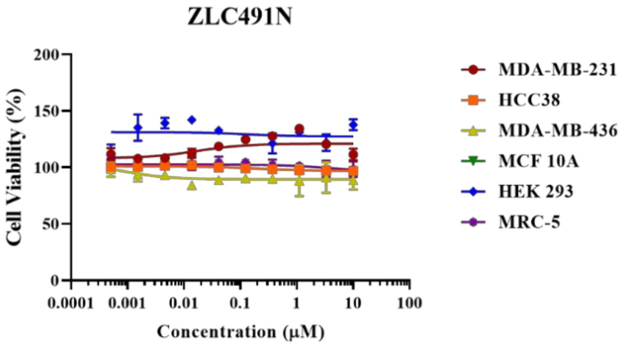

Figure S4. Cell antiproliferative activity of **ZLC491N** in multiple TNBC cells as well as noncancerous cells. Cells were treated with the compound for 5 days.

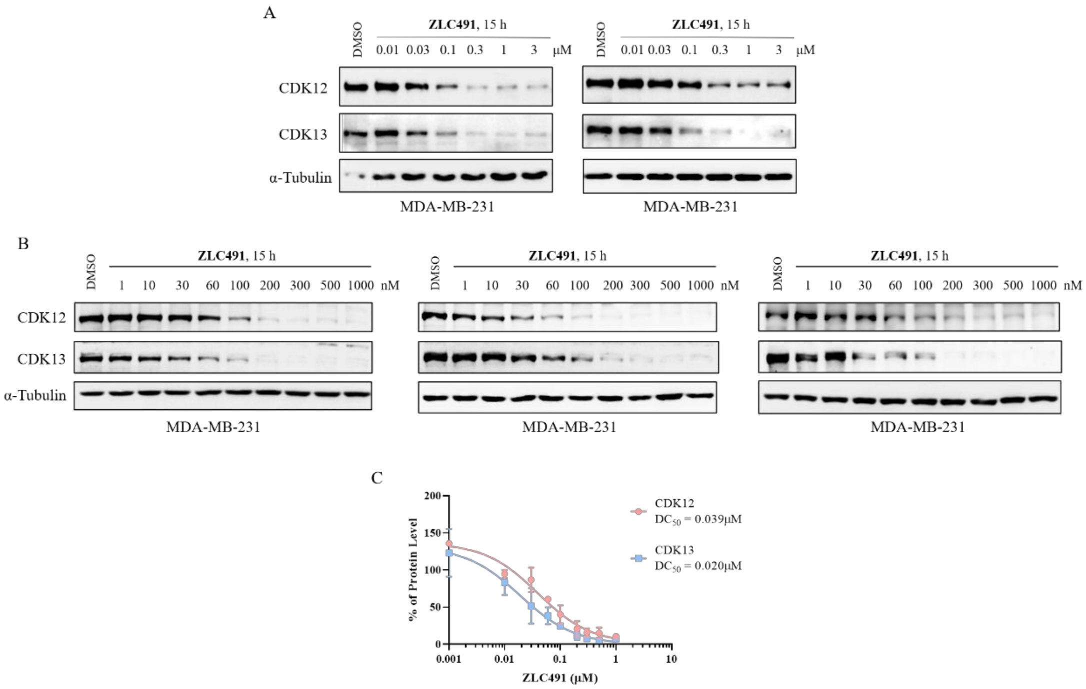

Figure S5. (A) Replications in the DC_{50} determination of **ZLC491** in MDA-MB-231 cells by immunoblotting of CDK12 and CDK13. Cells were treated with increasing concentrations of **ZLC491** for 15 h. (B) Immunoblotting of CDK12 and CDK13 in MDA-MB-231 cells treated with **ZLC491** within nanomolar concentrations for 15 h. The degradation detection was performed in triplicates. α-Tubulin was used as a loading control. (C) Protein levels in Figure S5B were quantified using ImageJ and normalized to corresponding α-Tubulin, then plotted using GraphPad Prism 8.0. The data were representative of three independent experiments.

Table S1 Primers used in RT-qPCR.

Primers	Sequences (5' to 3')
ATM-F	GCTGACAATCATCACCAAGT
ATM-R	GGTTCTCAGCACTATGGGACA
ATR-F	CGCTGAACTGTACGTGGAAA
ATR-R	CAATTAGTGCCTGGTGAACATC
BRCA1-F	CTGCTCAGGGCTATCCTCTCA
BRCA1-R	GCTTCTAGTTCAGCCATTTCCTG
BRCA2-F	AATGTCAGACAAGCTCAAAG
BRCA2-R	TCATGTATTTTTCAGGTGGC
FANCD2-F	CCCAGAACTGATCAACTCTCCT
FANCD2-R	CCATCATCACACGGAAGAAA
SMARACC-F	GAGGGATAAGCAGGTTCTTCTG
SMRAACC-R	GGGATCCACGTGTCGTAACT
GAPDH-F	TCACCAGGGCTGCTTTTAAC
GAPDH-R	ATCTCGCTCCTGGAAGATGG
ROR1-F	CCCAGTATCCCCACACACAC
ROR1-R	CGCTGGGATGTCACACAGAT

Scheme S1. Synthesis of Compound 13

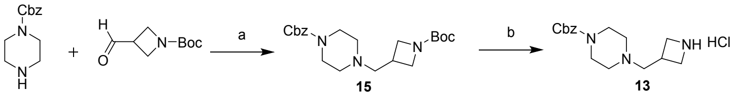

[a]**Reagents and conditions:** (a) $NaBH(OAc)_3$, CH_3COOH, CH_2Cl_2, 12 h, rt; (b) 4 M HCl in 1,4-dioxane, 12 h, rt.

Step 1. To a solution of benzyl 1-piperazinecarboxylate (1.13 g, 5.15 mmol) in anhydrous CH_2Cl_2 (10 mL) were added *tert*-butyl 3-formylazetidine-1-carboxylate (1 g, 5.4 mmol), CH_3COOH (0.3 mL) and $NaBH(OAc)_3$ (2.2 g, 10.3 mmol). The resulting suspension was then evacuated and backfilled with argon (3 cycles) and stirred for 12 h. After completion of the reaction, the solution was concentrated under reduced pressure, which was added to saturated Na_2CO_3 solution and extracted with EA. The organic layer was washed with brine, dried over anhydrous Na_2SO_4, and purified using column chromatography to afford target compound **15** (1.4 g, 70 %). ^1H NMR (500 MHz, Chloroform-*d*) δ 7.38 – 7.28 (m, 5H), 5.12 (d, J = 3.3 Hz, 2H), 4.01 – 3.97 (m, 2H), 3.60 – 3.54 (m, 2H), 3.48 (m, 4H), 2.70 (s, 1H), 2.57 (dd, J = 7.7, 3.3 Hz, 2H), 2.36 (s, 4H), 1.42 (s, 9H).

Step 2. To a solution of compound **15** (233 mg, 0.6 mmol) in anhydrous CH_2Cl_2 (2 mL) was added 4 M HCl 1,4-dioxane solution (2 mL). The resulting suspension was stirred for 12 h. After completion, the reaction solution was concentrated under reduced pressure to provide white solid (140 mg), which was used directly in next step without further purification (for synthesis **14a**).

Scheme S2. Synthesis of Compound 7c

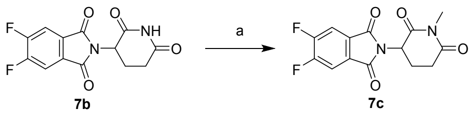

*a***Reagents and conditions:** (a) iodomethane, K_2CO_3, DMF, 60 °C, 2 h.

To a solution of compound **7b** (294 mg, 1 mmol) in anhydrous DMF (2 mL) were added K_2CO_3 (166 mg, 1.2 mmol) and iodomethane (68 μL). The resulting suspension was then heated at 60 °C for 2 h. After cooling, the reaction solution was added to ammonium chloride solution. The solvent was extracted with EA, dried over anhydrous Na_2SO_4, and evaporated under reduced pressure to afford target compound **7c** (180 mg, 78 %). ^{1}H NMR (500 MHz, DMSO-d_6) δ 8.17 – 8.14 (m, 2H), 5.27 – 5.21 (m, 1H), 3.02 (d, J = 1.6 Hz, 3H), 3.00 – 2.91 (m, 1H), 2.80 – 2.75 (m, 1H), 2.59 – 2.52 (m, 1H), 2.11 – 2.05 (m, 1H).

The ^{1}H NMR, ^{13}C NMR, HRMS and HPLC data of final compounds

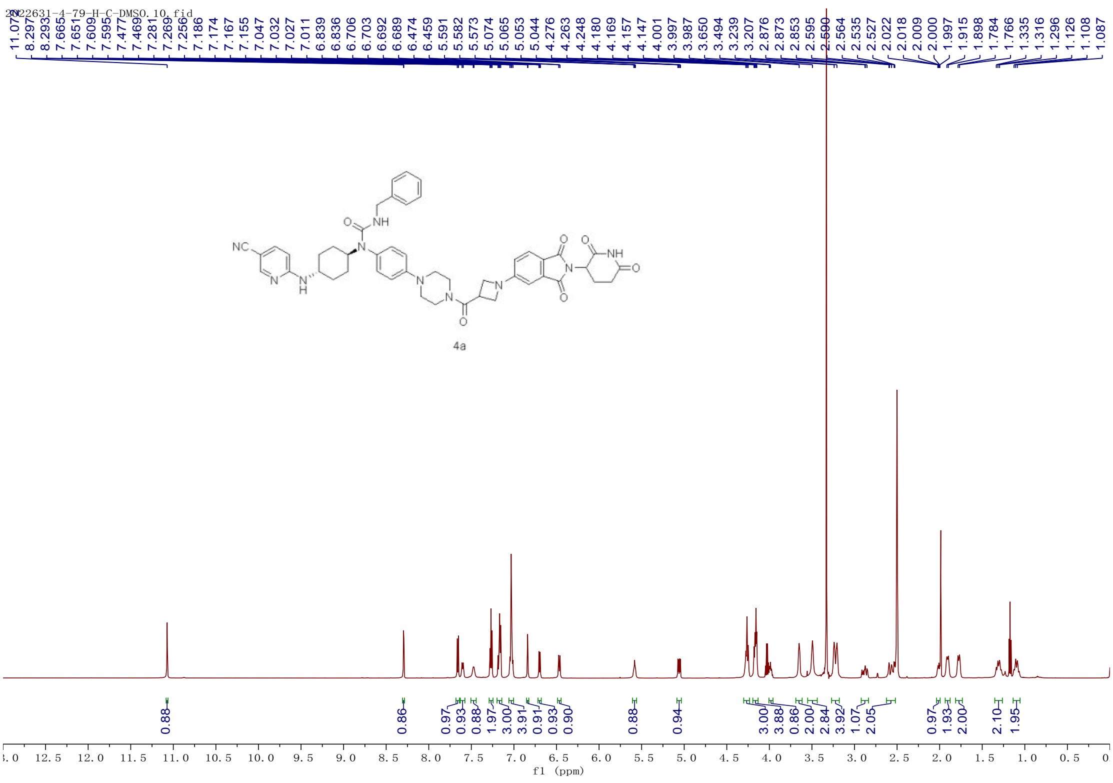

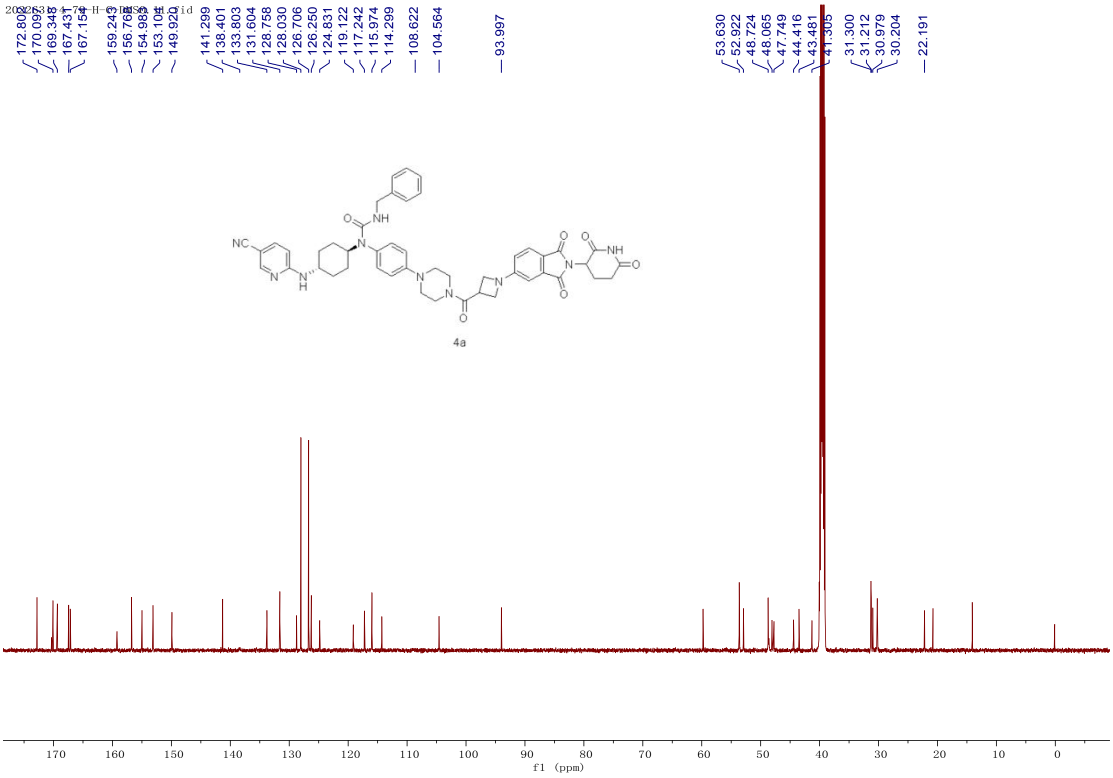

Mass Spectrum SmartFormula Report

**Analysis Info**		Acquisition Date	2/22/2023 4:35:30 PM
Analysis Name	D:\Data\SHUJVFENXI\DINGKE-GROUP\2022631-ZLC-4-79_RC6_01_34547.d		
Method	20150915.m	Operator	BDAL@DE
Sample Name	2022631-ZLC-4-79	Instrument / Ser#	maXis 4G 21240
Comment			

Acquisition Parameter

Source Type	ESI	Ion Polarity	Positive	Set Nebulizer	1.0 Bar
Focus	Not active	Set Capillary	3000 V	Set Dry Heater	220 °C
Scan Begin	50 m/z	Set End Plate Offset	-500 V	Set Dry Gas	6.0 l/min
Scan End	1500 m/z	Set Collision Cell RF	600.0 Vpp	Set Divert Valve	Waste

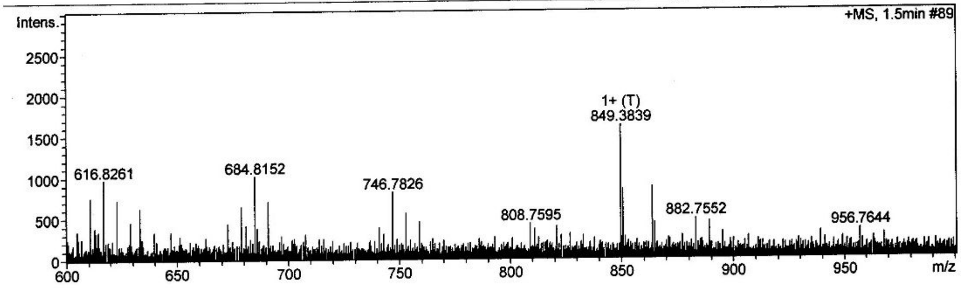

Meas. m/z	#	Formula	Score	m/z	err [ppm]	Mean err [ppm]	mSigma	rdb	e⁻ Conf	N-Rule
849.3839	1	C 47 H 49 N 10 O 6	100.00	849.3831	-0.9	-0.9	20.5	28.5	even	ok

**Data file:**	zlc-4-79.dx		
**Sequence Name:**	zlc-4-79	**Project Name:**	1260
**Sample name:**	zlc-4-79	**Operator:**	SYSTEM
**Instrument:**	1260	**Injection date:**	2023-02-16 12:36:03+08:00
**Inj. volume:**	10.000 µL	**Location:**	P1-C1
**Acq. method:**	normal.amx	**Type:**	Sample
**Processing method:**	GC_LC Area Percent_DefaultMethod.pmx	**Sample amount:**	0.00
**Manually modified:**	Manual Integration		

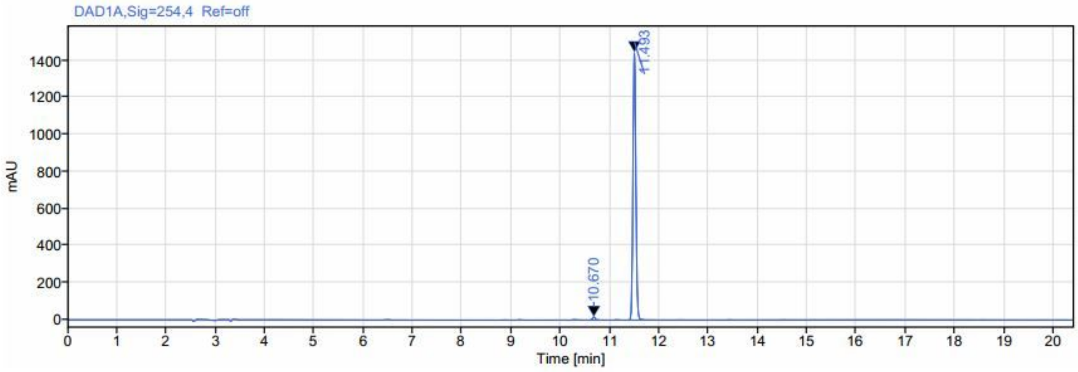

Signal: DAD1A,Sig=254,4 Ref=off

RT [min]	Type	Width [min]	Area	Height	Area%	Name
10.670	BB	0.45	69.97	16.83	1.16	
11.493	BV	0.41	5937.35	1445.24	98.84	
		**Sum**	6007.31		100.00	

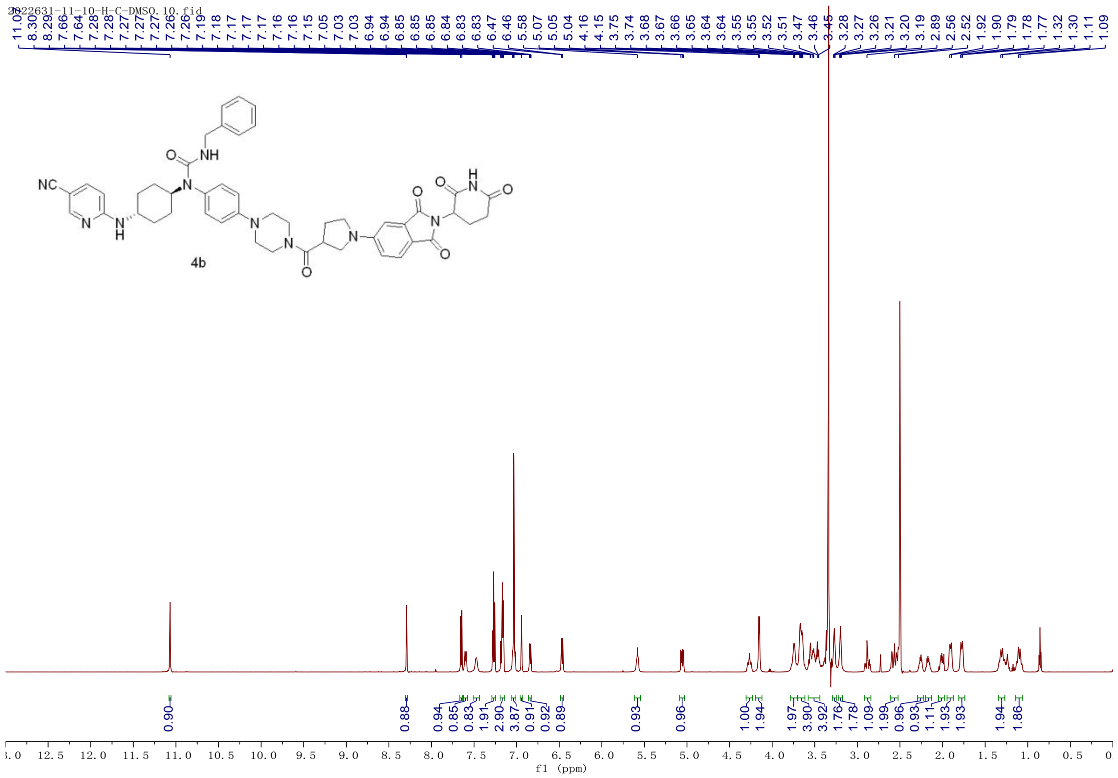
2022631-11-10-H-C-DMSO.10.fid
11.09 8.30 8.29 7.66 7.64 7.28 7.27 7.27 7.27 7.26 7.26 7.19 7.18 7.17 7.17 7.17 7.16 7.15 7.05 7.03 6.94 6.94 6.85 6.85 6.84 6.83 6.83 6.47 6.46 5.58 5.07 5.05 5.04 4.16 4.15 3.75 3.74 3.68 3.67 3.66 3.65 3.64 3.64 3.55 3.55 3.52 3.51 3.47 3.46 3.45 3.28 3.27 3.26 3.21 3.20 3.19 2.89 2.56 2.52 1.92 1.90 1.79 1.78 1.77 1.32 1.30 1.11 1.09
NC
NH
O
N
H
O
4b
0.90 0.88 0.94 0.85 0.83 1.91 2.90 3.87 0.91 0.92 0.89 0.93 0.96 1.00 1.94 1.97 3.90 3.92 1.76 1.78 1.09 1.99 0.96 0.93 1.11 1.93 1.93 1.94 1.86
f1 (ppm)

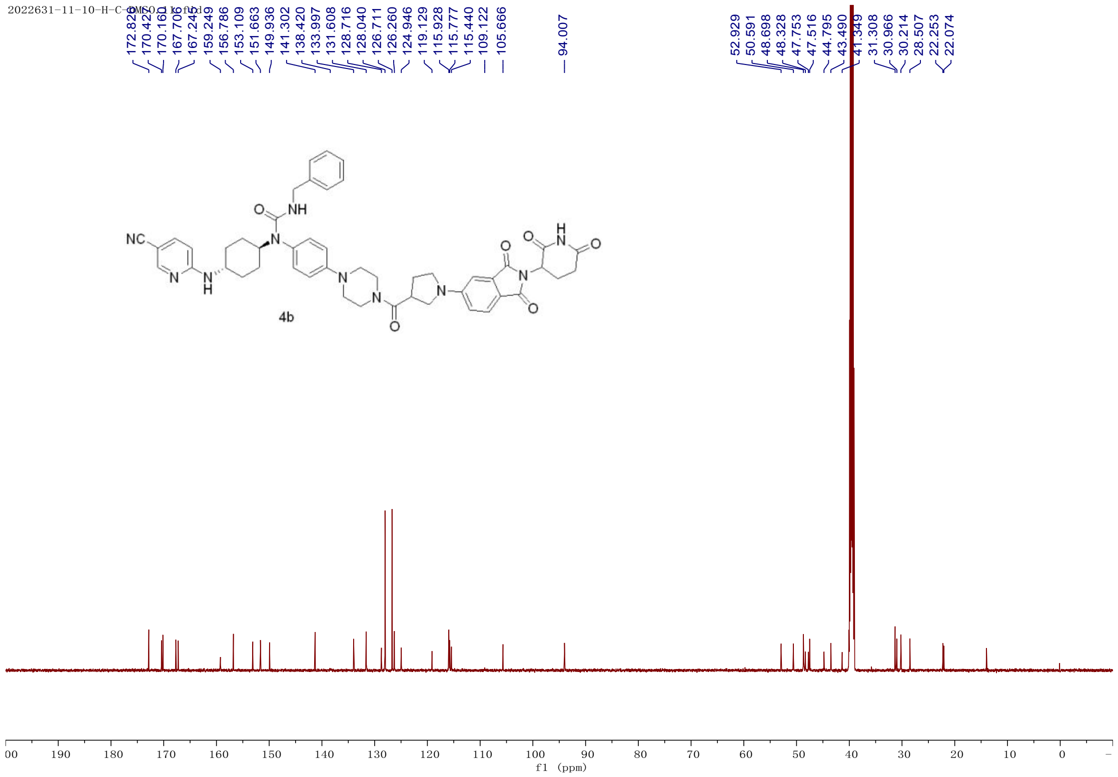
2022631-11-10-H-C-DMSO.11.fid
172.826 170.422 170.161 167.706 167.245 159.249 156.786 153.109 151.663 149.936 141.302 138.420 133.997 131.608 128.716 128.040 126.711 126.260 124.946 119.129 115.928 115.777 115.440 109.122 105.666 94.007 52.929 50.591 48.698 48.328 47.753 47.516 44.795 43.490 41.349 31.308 30.966 30.214 28.507 22.253 22.074
NC
NH
O
N
H
O
4b
f1 (ppm)

Mass Spectrum SmartFormula Report

Analysis Info

Analysis Name	D:\Data\SHUJVFENXI\DINGKE-GROUP\2022631-ZLC-11-10_RB7_01_45158.d	Acquisition Date	6/19/2024 11:55:58 AM
Method	6min.m	Operator	BDAL@DE
Sample Name	2022631-ZLC-11-10	Instrument / Ser#	maXis 4G 21240
Comment			

Acquisition Parameter

Source Type	ESI	Ion Polarity	Positive	Set Nebulizer	1.0 Bar
Focus	Not active	Set Capillary	3000 V	Set Dry Heater	220 °C
Scan Begin	50 m/z	Set End Plate Offset	-500 V	Set Dry Gas	6.0 l/min
Scan End	1500 m/z	Set Collision Cell RF	600.0 Vpp	Set Divert Valve	Waste

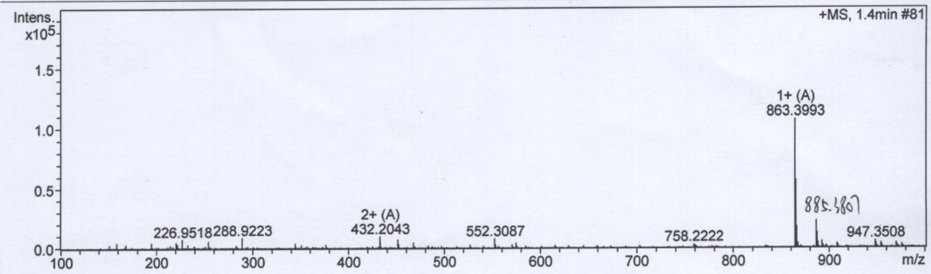

Meas. m/z	#	Formula	Score	m/z	err [ppm]	Mean err [ppm]	mSigma	rdb	e⁻ Conf	N-Rule
863.3993	1	C 48 H 51 N 10 O 6	100.00	863.3988	-0.6	-0.2	16.7	28.5	even	ok
885.3807	1	C 48 H 50 N 10 Na O 6	100.00	885.3807	0.1	0.2	10.2	28.5	even	ok

**Data file:**	zlc-11-10-1--.dx		
**Sequence Name:**	SingleSample	**Project Name:**	1260
**Sample name:**	zlc-11-10-1--	**Operator:**	SYSTEM
**Instrument:**	1260	**Injection date:**	2024-06-18 16:09:04+08:00
**Inj. volume:**	10.000 µL	**Location:**	P1-B2
**Acq. method:**	normal.amx	**Type:**	Sample
**Processing method:**	INM.pmx	**Sample amount:**	0.00
**Manually modified:**	Manual Integration		

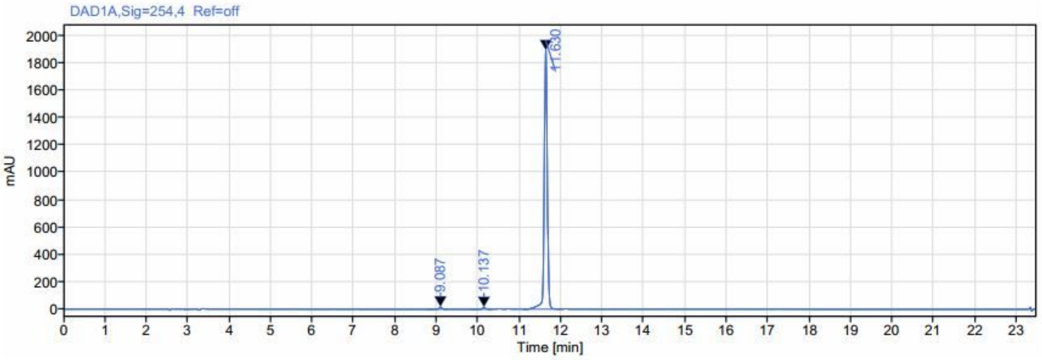

Signal: DAD1A,Sig=254,4 Ref=off

RT [min]	Type	Width [min]	Area	Height	Area%	Name
9.087	VV	0.50	79.20	16.10	0.80	
10.137	BV	0.49	64.61	13.19	0.66	
11.630	BV	0.93	9714.16	1895.84	98.54	
		**Sum**	9857.97		100.00	

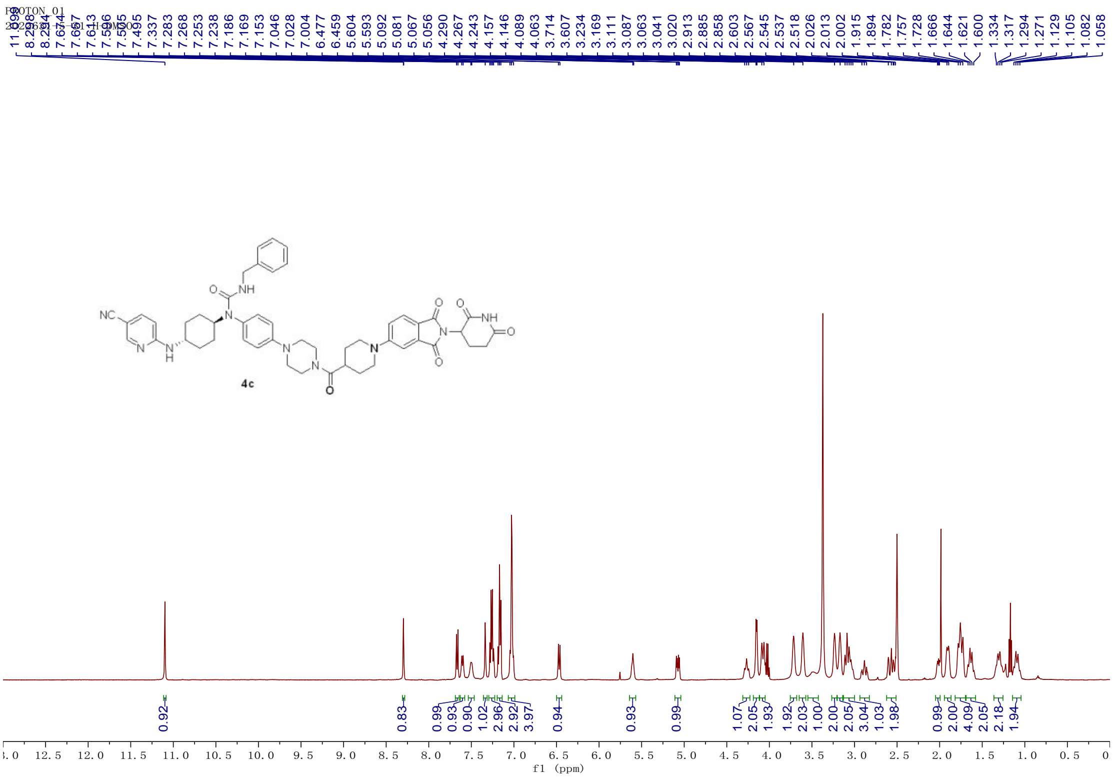

PROTON_01
8.298 8.294 7.674 7.667 7.613 7.596 7.505 7.495 7.337 7.283 7.268 7.253 7.238 7.186 7.169 7.153 7.046 7.028 7.004 6.477 6.459 5.604 5.593 5.092 5.081 5.067 5.056 4.290 4.267 4.243 4.157 4.146 4.089 4.063 3.714 3.607 3.234 3.169 3.111 3.087 3.063 3.041 3.020 2.913 2.885 2.858 2.603 2.567 2.545 2.537 2.518 2.026 2.013 2.002 1.915 1.894 1.782 1.757 1.728 1.666 1.644 1.621 1.600 1.334 1.317 1.294 1.271 1.129 1.105 1.082 1.058
NC
N
H
O
NH
4c
0.92 0.83 0.99 0.93 0.90 1.02 2.96 2.92 3.97 0.94 0.93 0.99 1.07 2.05 1.93 1.92 2.03 1.00 2.00 2.05 3.04 1.03 1.98 0.99 2.00 4.09 2.05 2.18 1.94
12.5 12.0 11.5 11.0 10.5 10.0 9.5 9.0 8.5 8.0 7.5 7.0 6.5 6.0 5.5 5.0 4.5 4.0 3.5 3.0 2.5 2.0 1.5 1.0 0.5 0
f1 (ppm)

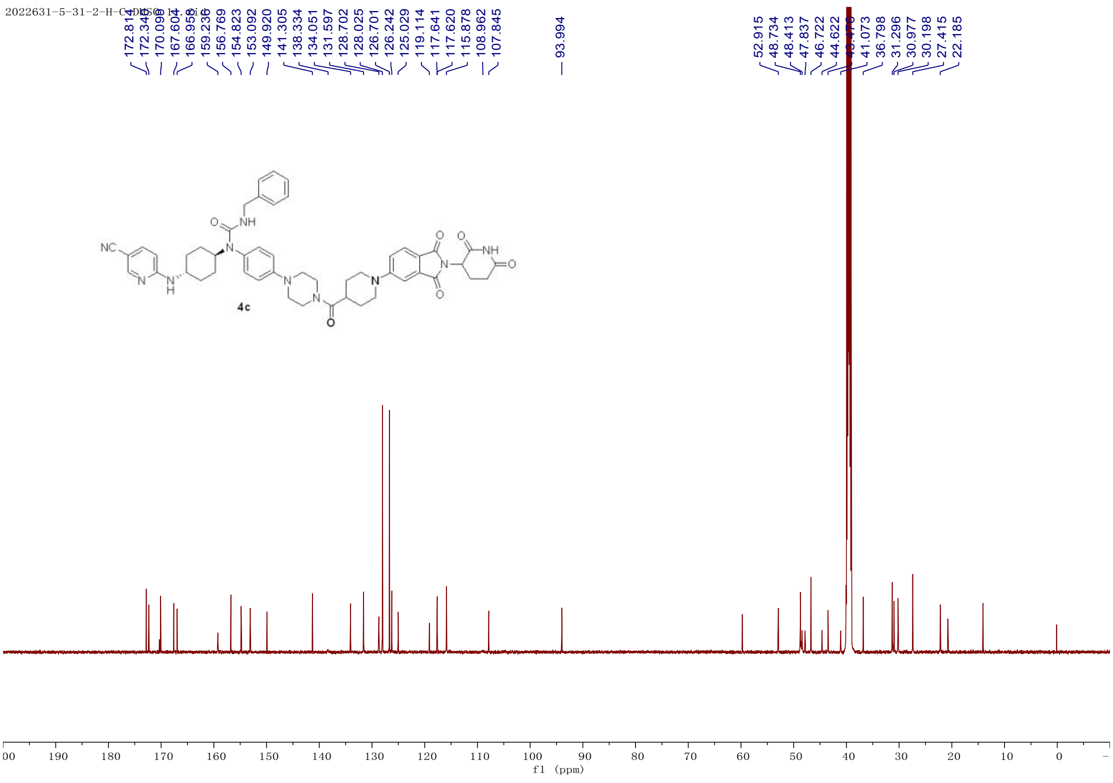

172.814 172.340 170.099 167.604 166.958 159.236 156.769 154.823 153.092 149.920 141.305 138.334 134.051 131.597 128.702 128.025 126.701 126.242 125.029 119.114 117.641 117.620 115.878 108.962 107.845 93.994 52.915 48.734 48.413 47.837 46.722 44.622 41.073 36.798 31.296 30.977 30.198 27.415 22.185
NC
N
H
O
NH
4c
190 180 170 160 150 140 130 120 110 100 90 80 70 60 50 40 30 20 10 0
f1 (ppm)

Mass Spectrum SmartFormula Report

Analysis Info			
Analysis Name	D:\Data\SHUJVFENXI\DINGKE-GROUP\2022631-ZLC-5-31_RB5_01_37285.d	Acquisition Date	6/1/2023 4:33:26 PM
Method	20150915.m	Operator	BDAL@DE
Sample Name	2022631-ZLC-5-31	Instrument / Ser#	maXis 4G 21240
Comment			

Acquisition Parameter

Source Type	ESI	Ion Polarity	Positive	Set Nebulizer	1.0 Bar
Focus	Not active	Set Capillary	3000 V	Set Dry Heater	220 °C
Scan Begin	50 m/z	Set End Plate Offset	-500 V	Set Dry Gas	6.0 l/min
Scan End	1500 m/z	Set Collision Cell RF	600.0 Vpp	Set Divert Valve	Waste

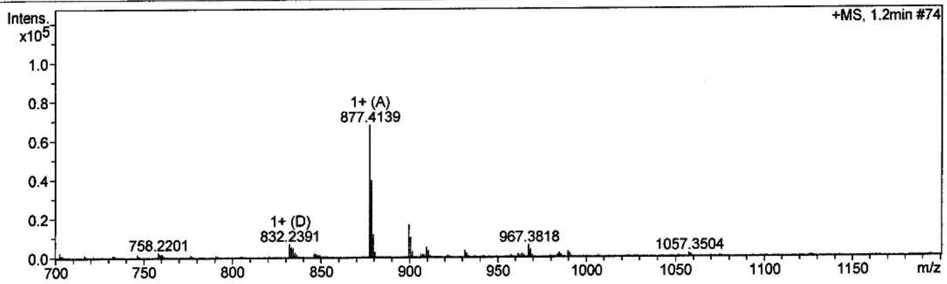

Meas. m/z	#	Formula	Score	m/z	err [ppm]	Mean err [ppm]	mSigma	rdb	e⁻ Conf	N-Rule
877.4139	1	C 49 H 53 N 10 O 6	100.00	877.4144	0.6	0.7	4.9	28.5	even	ok

**Data file:**	zlc-5-31-1.dx		
**Sequence Name:**	SingleSample	**Project Name:**	1260
**Sample name:**	zlc-5-31-1	**Operator:**	SYSTEM
**Instrument:**	1260	**Injection date:**	2023-03-23 16:42:57+08:00
**Inj. volume:**	10.000 µL	**Location:**	P1-A3
**Acq. method:**	normal.amx	**Type:**	Sample
**Processing method:**	GC_LC 面积百分比_DefaultMethod.pmx	**Sample amount:**	0.00
**Manually modified:**	Manual Integration		

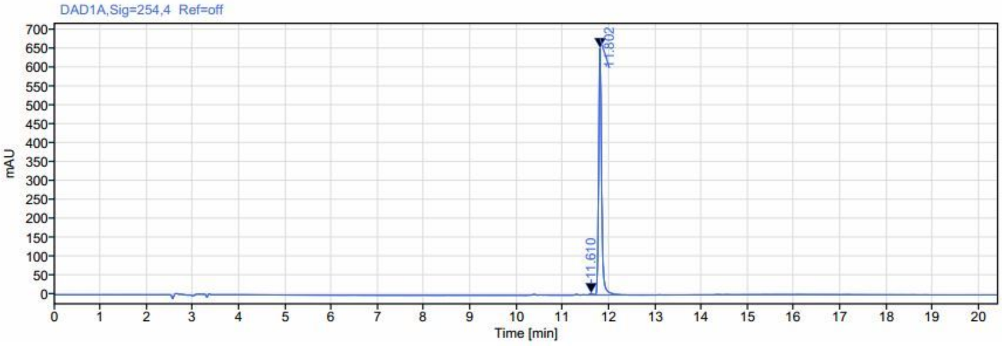

Signal: DAD1A,Sig=254,4 Ref=off

RT [min]	Type	Width [min]	Area	Height	Area%	Name
11.610	VV	0.18	22.85	4.36	0.76	
11.802	VV	0.62	2997.40	653.39	99.24	
		**Sum**	3020.25		100.00	

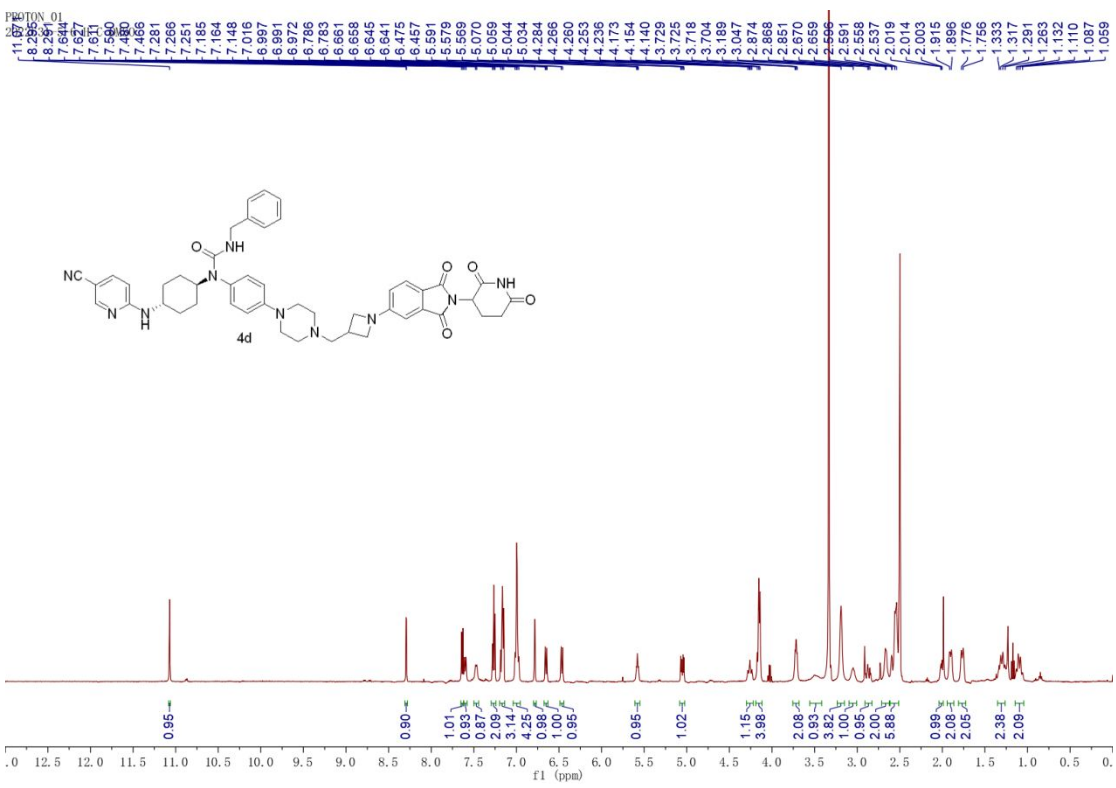
8.291
7.644
7.627
7.611
7.466
7.281
7.266
7.251
7.185
7.164
7.148
7.016
6.997
6.991
6.972
6.786
6.783
6.661
6.658
6.645
6.641
6.475
6.457
5.591
5.579
5.569
5.070
5.059
5.044
5.034
4.284
4.266
4.260
4.253
4.236
4.173
4.154
4.140
3.729
3.725
3.718
3.704
3.189
3.047
2.874
2.868
2.851
2.670
2.659
2.591
2.558
2.537
2.019
2.014
2.003
1.915
1.896
1.776
1.756
1.333
1.317
1.291
1.263
1.132
1.110
1.087
1.059
NC
N
H
NH
O
N
4d
0.95
0.90
1.01
0.93
0.87
2.09
3.14
4.25
0.98
1.00
0.95
0.95
1.02
1.15
3.98
2.08
0.93
3.82
1.00
0.95
2.00
5.88
0.99
2.08
2.05
2.38
2.09
f1 (ppm)

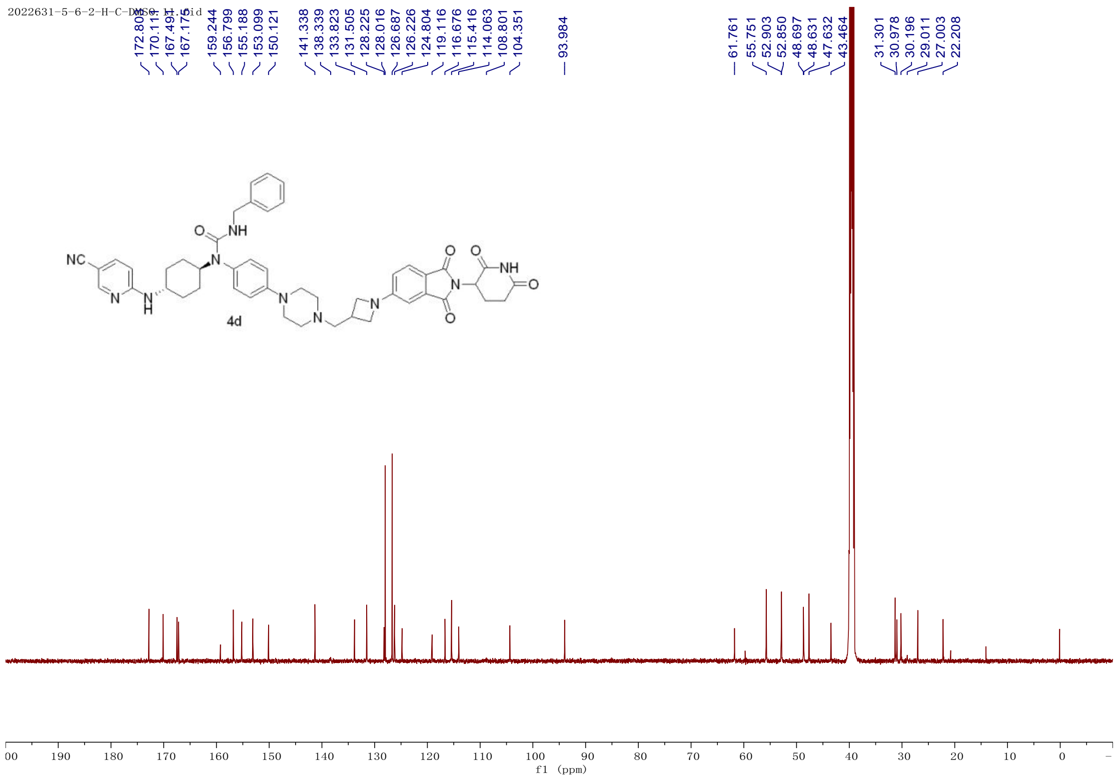
172.808
167.491
167.175
159.244
156.799
155.188
153.099
150.121
141.338
138.339
133.823
131.505
128.225
128.016
126.687
126.226
124.804
119.116
116.676
115.416
114.063
108.801
104.351
93.984
61.761
55.751
52.903
52.850
48.697
48.631
47.632
43.464
31.301
30.978
30.196
29.011
27.003
22.208
4d
f1 (ppm)

Mass Spectrum SmartFormula Report

Analysis Info			
Analysis Name	D:\Data\SHUJVFENXI\DINGKE-GROUP\2022631-ZLC-5-6_BB1_01_35731.d	Acquisition Date	3/21/2023 10:05:40 PM
Method	20150915.m	Operator	BDAL@DE
Sample Name	2022631-ZLC-5-6	Instrument / Ser#	maXis 4G 21240
Comment			

Acquisition Parameter

Source Type	ESI	Ion Polarity	Positive	Set Nebulizer	1.0 Bar
Focus	Not active	Set Capillary	3000 V	Set Dry Heater	220 °C
Scan Begin	50 m/z	Set End Plate Offset	-500 V	Set Dry Gas	6.0 l/min
Scan End	1500 m/z	Set Collision Cell RF	600.0 Vpp	Set Divert Valve	Waste

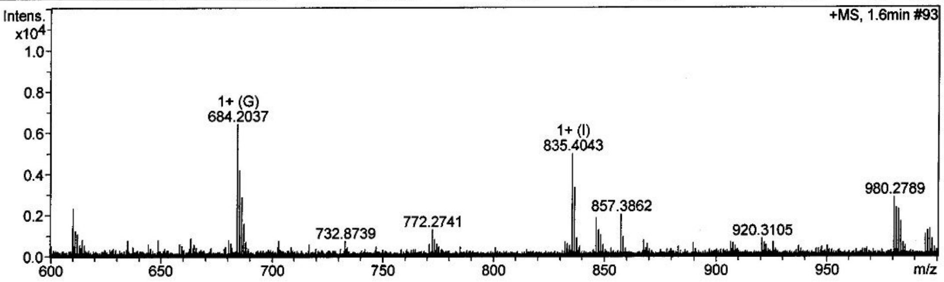

Meas. m/z	#	Formula	Score	m/z	err [ppm]	Mean err [ppm]	mSigma	rdb	e⁻ Conf	N-Rule
835.4043	1	C 47 H 51 N 10 O 5	100.00	835.4038	-0.6	0.1	65.9	27.5	even	ok

**Data file:**	zlc-5-6-s2.dx		
**Sequence Name:**	zlc-5-6-s2	**Project Name:**	1260
**Sample name:**	zlc-5-6-s2	**Operator:**	SYSTEM
**Instrument:**	1260	**Injection date:**	2023-03-08 10:57:56+08:00
**Inj. volume:**	10.000 µL	**Location:**	P1-A3
**Acq. method:**	normal.amx	**Type:**	Sample
**Processing method:**	GC_LC Area Percent_DefaultMethod.pmx	**Sample amount:**	0.00
**Manually modified:**	Manual Integration		

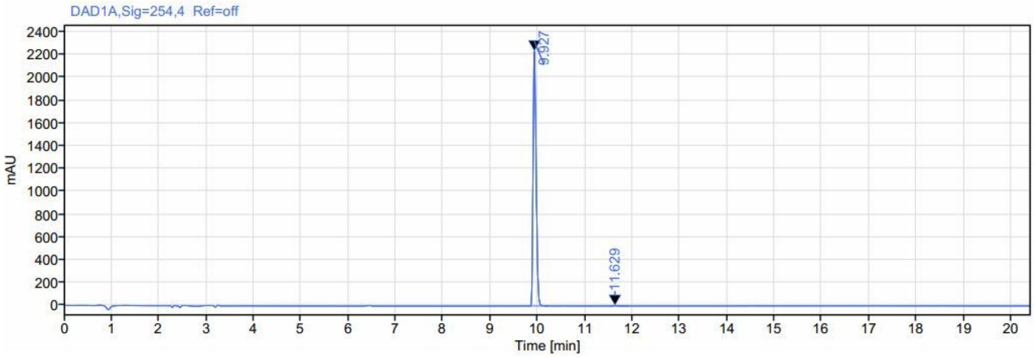

Signal: DAD1A,Sig=254,4 Ref=off

RT [min]	Type	Width [min]	Area	Height	Area%	Name
9.927	VV	0.38	10134.38	2236.57	99.75	
11.629	BV	0.39	25.14	3.69	0.25	
		**Sum**	10159.52		100.00	

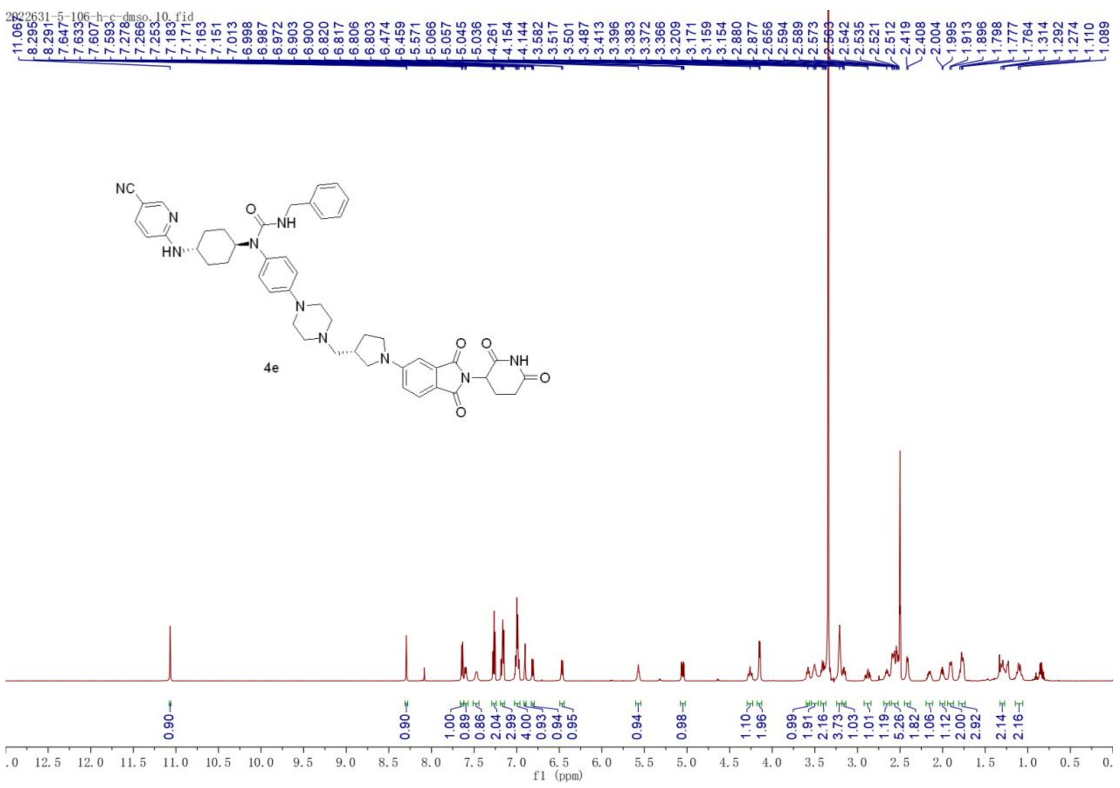
2022631-5-106-h-c-dmso.10.fid
NC
HN
NH
4e
f1 (ppm)

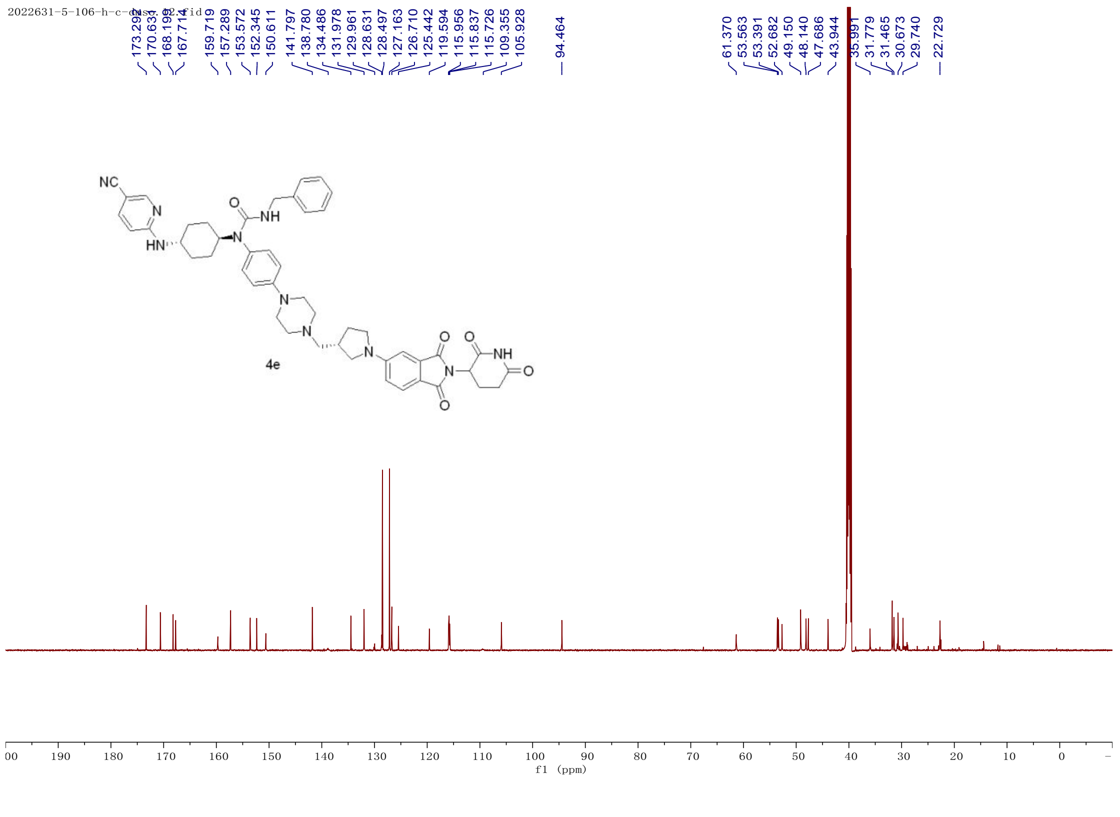
NC
HN
NH
4e
f1 (ppm)

Mass Spectrum SmartFormula Report

Analysis Info

Analysis Name	D:\Data\SHUJVFENXI\DINGKE-GROUP\2022631-zlc-5-106_RA5_01_37049.d	Acquisition Date	5/16/2023 5:48:37 PM
Method	20150915.m	Operator	BDAL@DE
Sample Name	2022631-zlc-5-106	Instrument / Ser#	maXis 4G 21240
Comment			

Acquisition Parameter

Source Type	ESI	Ion Polarity	Positive	Set Nebulizer	1.0 Bar
Focus	Not active	Set Capillary	3000 V	Set Dry Heater	220 °C
Scan Begin	50 m/z	Set End Plate Offset	-500 V	Set Dry Gas	6.0 l/min
Scan End	1500 m/z	Set Collision Cell RF	600.0 Vpp	Set Divert Valve	Waste

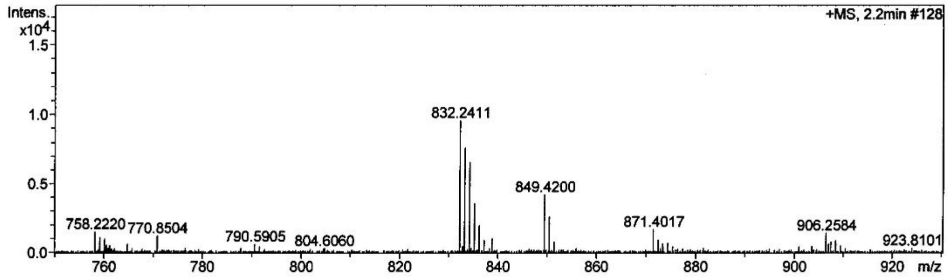

Meas. m/z	#	Formula	Score	m/z	err [ppm]	Mean err [ppm]	mSigma	rdb	e⁻ Conf	N-Rule
849.4200	1	C 48 H 53 N 10 O 5	100.00	849.4195	-0.6	-0.4	30.8	27.5	even	ok
871.4017	1	C 48 H 52 N 10 Na O 5	100.00	871.4014	-0.3	-0.1	36.3	27.5	even	ok

**Data file:**	zlc-5-106.dx		
**Sequence Name:**	zlc-5-103-zlc-5-106	**Project Name:**	1260
**Sample name:**	zlc-5-106	**Operator:**	SYSTEM
**Instrument:**	1260	**Injection date:**	2023-05-13 22:49:48+08:00
**Inj. volume:**	10.000 µL	**Location:**	P1-D2
**Acq. method:**	normal.amx	**Type:**	Sample
**Processing method:**	GC_LC Area Percent_DefaultMethod.pmx	**Sample amount:**	0.00
**Manually modified:**	Manual Integration		

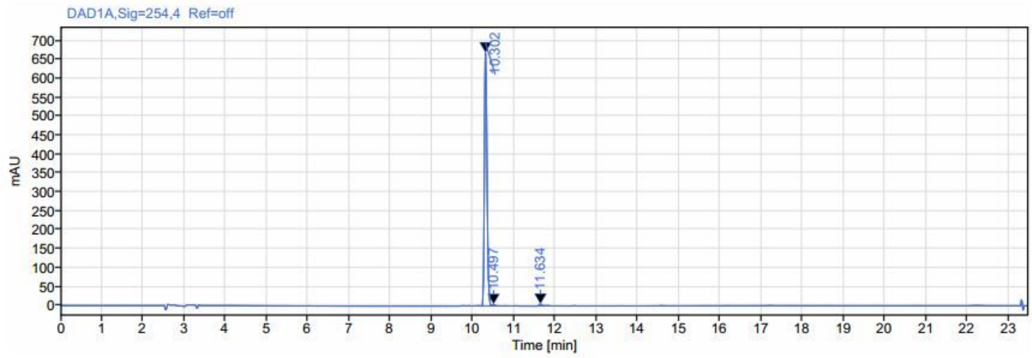

Signal: DAD1A,Sig=254,4 Ref=off

RT [min]	Type	Width [min]	Area	Height	Area%	Name
10.302	BV	0.25	2981.19	669.38	98.55	
10.497	VV	0.13	17.78	4.07	0.59	
11.634	BV	0.28	26.10	4.80	0.86	
		**Sum**	3025.07		100.00	

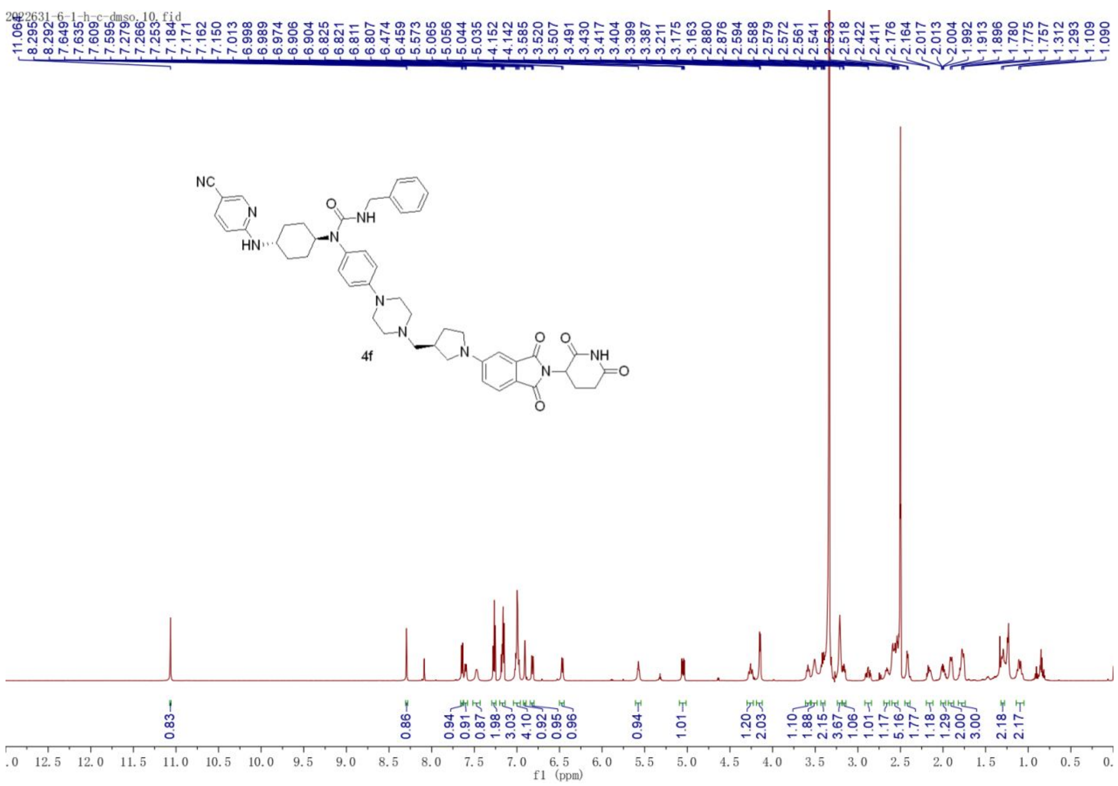
NC
N
HN
O
NH
4f
O
NH
O
f1 (ppm)

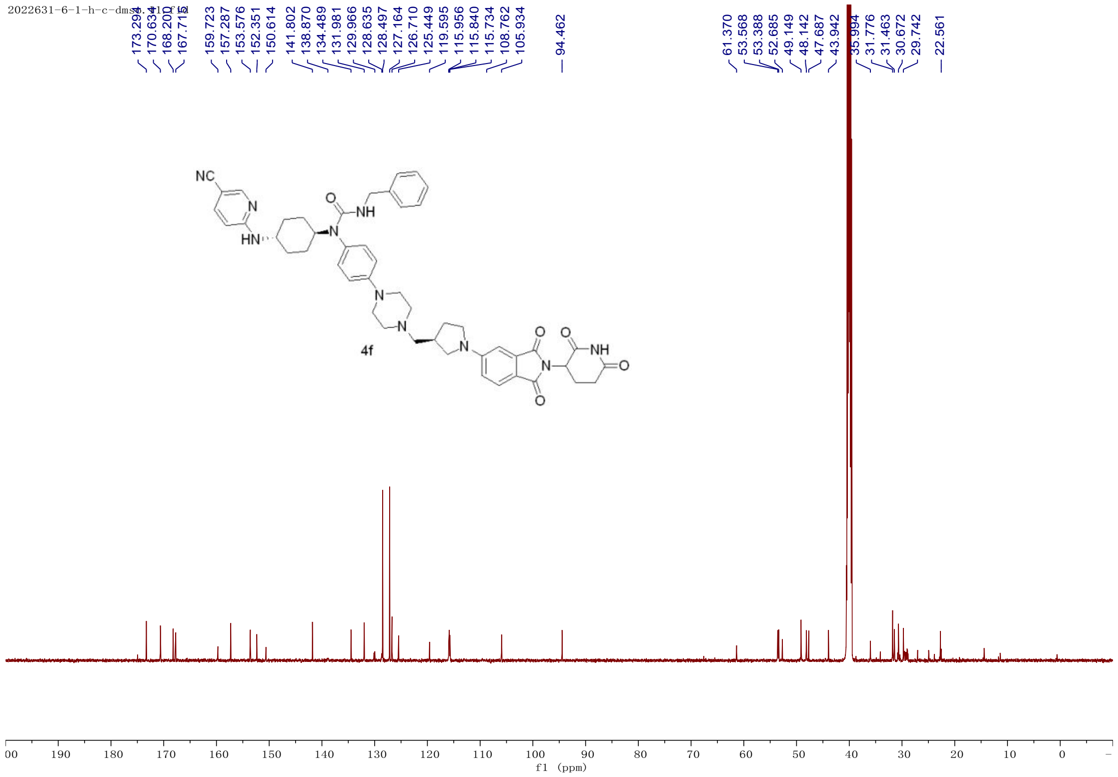
NC
N
HN
O
NH
4f
O
NH
O
f1 (ppm)

Mass Spectrum SmartFormula Report

**Analysis Info**		Acquisition Date	5/16/2023 11:38:31 AM
Analysis Name	D:\Data\SHUJVFENXI\DINGKE-GROUP\2022631-zlc-6-1_RA4_01_36993.d		
Method	20150915.m	Operator	BDAL@DE
Sample Name	2022631-zlc-6-1	Instrument / Ser#	maXis 4G 21240
Comment			

Acquisition Parameter

Source Type	ESI	Ion Polarity	Positive	Set Nebulizer	1.0 Bar
Focus	Not active	Set Capillary	3000 V	Set Dry Heater	220 °C
Scan Begin	50 m/z	Set End Plate Offset	-500 V	Set Dry Gas	6.0 l/min
Scan End	1500 m/z	Set Collision Cell RF	600.0 Vpp	Set Divert Valve	Waste

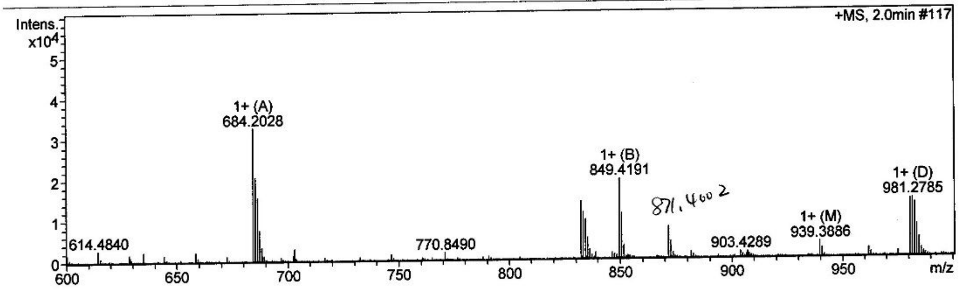

Meas. m/z	#	Formula	Score	m/z	err [ppm]	Mean err [ppm]	mSigma	rdb	e⁻ Conf	N-Rule
849.4191	1	C 48 H 53 N 10 O 5	100.00	849.4195	0.4	0.6	8.2	27.5	even	ok
871.4002	1	C 48 H 52 N 10 Na O 5	100.00	871.4014	1.4	1.7	11.2	27.5	even	ok

**Data file:**	zlc-6-1.dx		
**Sequence Name:**	zlc-6-1	**Project Name:**	1260
**Sample name:**	zlc-6-1	**Operator:**	SYSTEM
**Instrument:**	1260	**Injection date:**	2023-05-13 00:42:43+08:00
**Inj. volume:**	10.000 µL	**Location:**	P1-D3
**Acq. method:**	normal.amx	**Type:**	Sample
**Processing method:**	GC_LC Area Percent_DefaultMethod.pmx	**Sample amount:**	0.00
**Manually modified:**	Manual Integration		

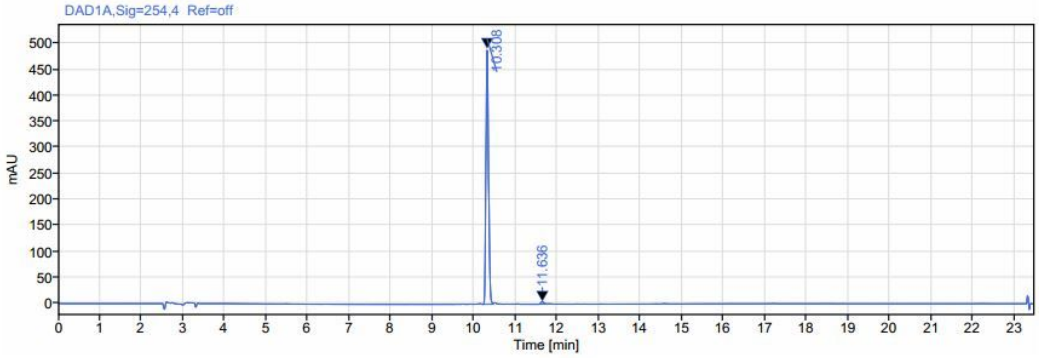

Signal: DAD1A,Sig=254,4 Ref=off

RT [min]	Type	Width [min]	Area	Height	Area%	Name
10.308	BV	0.24	2142.23	492.24	98.83	
11.636	BV	0.24	25.43	4.85	1.17	
		Sum	2167.66		100.00	

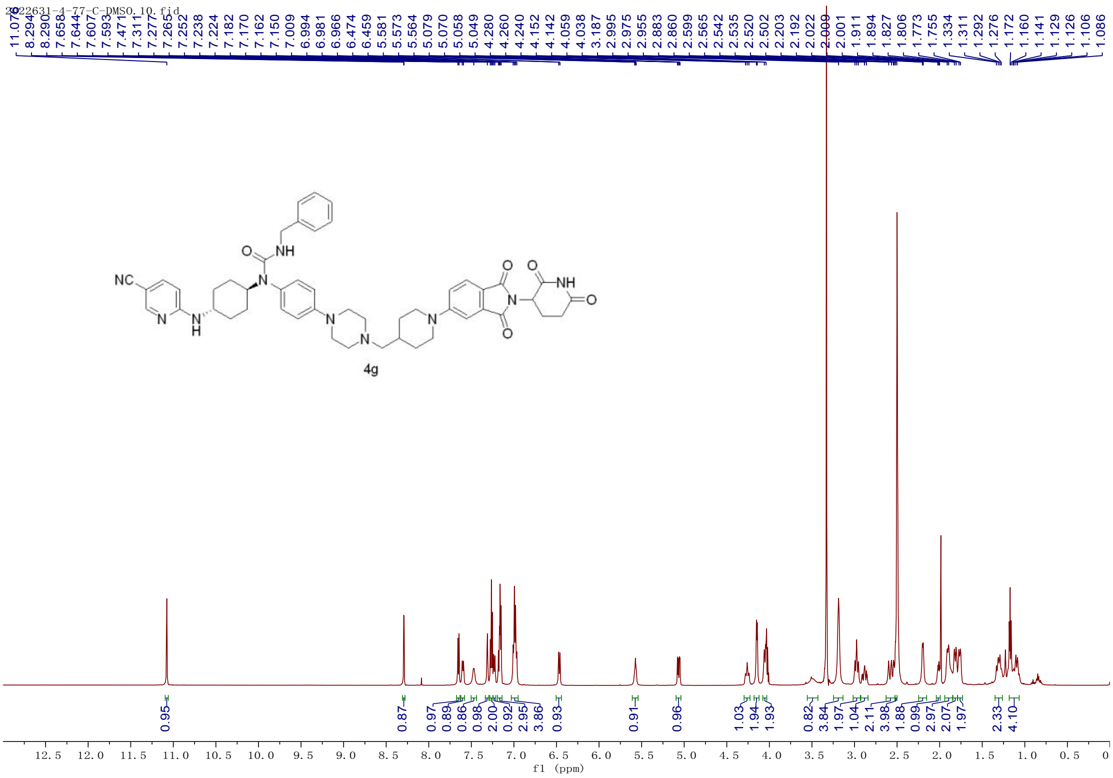
2022631-4-77-C-DMSO.10.fid
11.078 8.294 8.290 7.658 7.644 7.607 7.593 7.471 7.311 7.277 7.265 7.252 7.238 7.224 7.182 7.170 7.162 7.150 7.009 6.994 6.981 6.966 6.474 6.459 5.581 5.573 5.564 5.079 5.070 5.058 5.049 4.280 4.260 4.240 4.152 4.142 4.059 4.038 3.187 2.995 2.975 2.955 2.883 2.860 2.599 2.565 2.542 2.535 2.520 2.502 2.203 2.192 2.022 2.009 2.001 1.911 1.894 1.827 1.806 1.773 1.755 1.334 1.311 1.292 1.276 1.172 1.160 1.141 1.129 1.126 1.106 1.086
NC
N
H
O
NH
N
N
N
N
O
O
N
O
NH
O
4g
0.95 0.87 0.97 0.89 0.86 0.98 2.00 0.92 2.95 3.86 0.93 0.91 0.96 1.03 1.94 1.93 0.82 3.84 1.97 1.04 2.11 3.98 1.88 0.99 2.97 2.07 1.97 2.33 4.10
12.5 12.0 11.5 11.0 10.5 10.0 9.5 9.0 8.5 8.0 7.5 7.0 6.5 6.0 5.5 5.0 4.5 4.0 3.5 3.0 2.5 2.0 1.5 1.0 0.5 0
f1 (ppm)

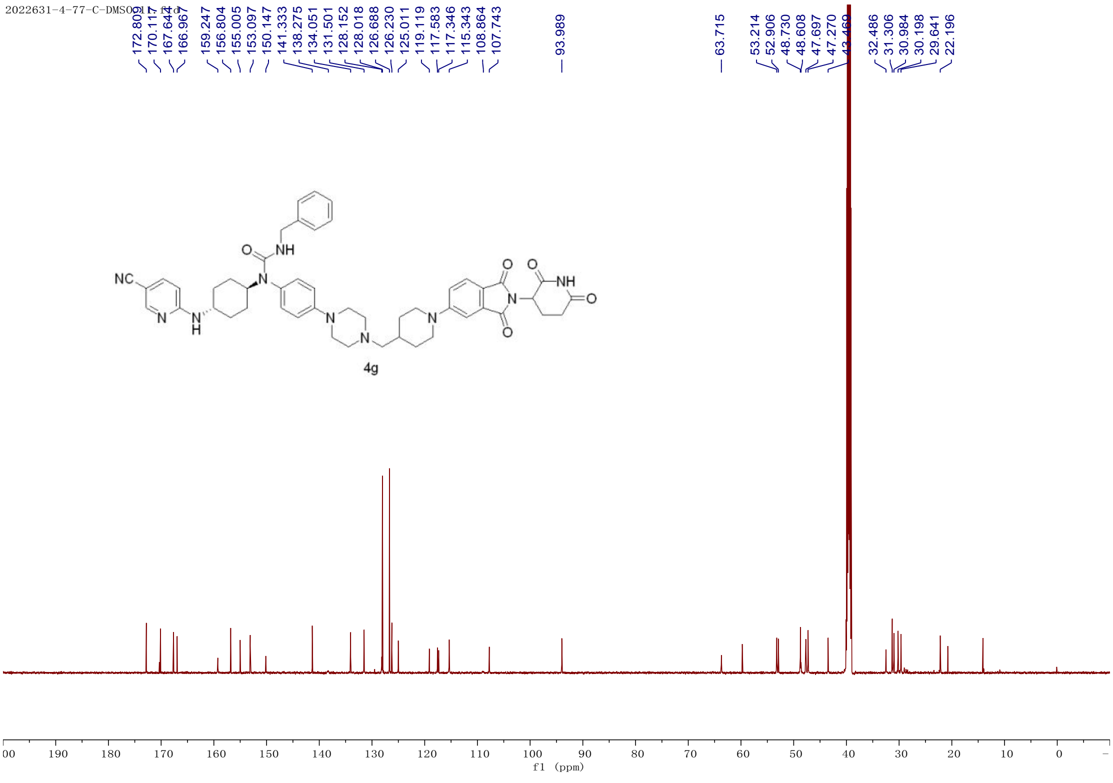
2022631-4-77-C-DMSO
172.809 170.117 167.644 166.967 159.247 156.804 155.005 153.097 150.147 141.333 138.275 134.051 131.501 128.152 128.018 126.688 126.230 125.011 119.119 117.583 117.346 115.343 108.864 107.743 93.989 63.715 53.214 52.906 48.730 48.608 47.697 47.270 32.486 31.306 30.984 30.198 29.641 22.196
NC
N
H
O
NH
N
N
N
N
O
O
N
O
NH
O
4g
190 180 170 160 150 140 130 120 110 100 90 80 70 60 50 40 30 20 10 0
f1 (ppm)

Mass Spectrum SmartFormula Report

Analysis Info

Analysis Name	D:\Data\SHUJVFENXI\DINGKE-GROUP\2022631-ZLC-4-77_RC5_01_34546.d
Method	20150915.m
Sample Name	2022631-ZLC-4-77
Comment	
Acquisition Date	2/22/2023 4:31:18 PM
Operator	BDAL@DE
Instrument / Ser#	maXis 4G 21240

Acquisition Parameter

Source Type	ESI	Ion Polarity	Positive	Set Nebulizer	1.0 Bar
Focus	Not active	Set Capillary	3000 V	Set Dry Heater	220 °C
Scan Begin	50 m/z	Set End Plate Offset	-500 V	Set Dry Gas	6.0 l/min
Scan End	1500 m/z	Set Collision Cell RF	600.0 Vpp	Set Divert Valve	Waste

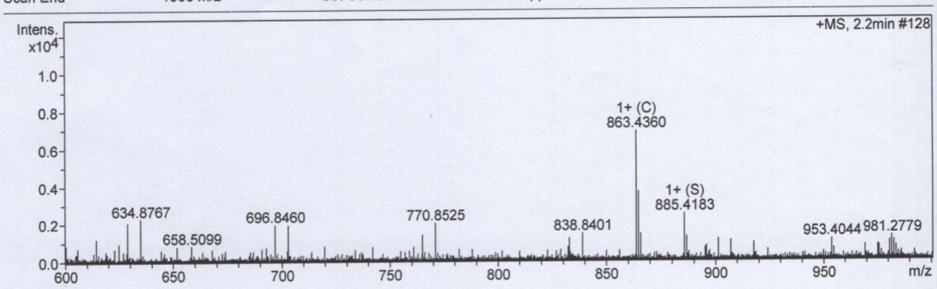

Meas. m/z	#	Formula	Score	m/z	err [ppm]	Mean err [ppm]	mSigma	rdb	e⁻ Conf	N-Rule
863.4360	1	C 49 H 55 N 10 O 5	100.00	863.4351	-0.9	-0.8	26.7	27.5	even	ok

**Data file:**	zlc-4-77.dx		
**Sequence Name:**	zlc-4-77	**Project Name:**	1260
**Sample name:**	zlc-4-77	**Operator:**	SYSTEM
**Instrument:**	1260	**Injection date:**	2023-02-14 15:05:58+08:00
**Inj. volume:**	10.000 µL	**Location:**	P1-C1
**Acq. method:**	normal.amx	**Type:**	Sample
**Processing method:**	GC_LC Area Percent_DefaultMethod.pmx	**Sample amount:**	0.00
**Manually modified:**	Manual Integration		

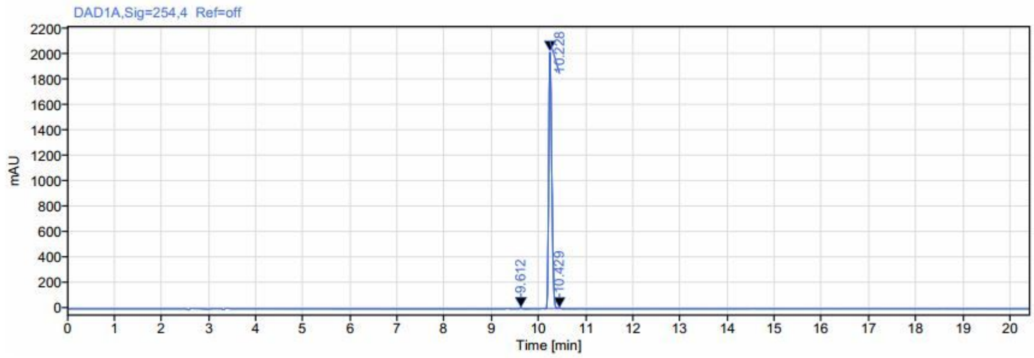

Signal: DAD1A,Sig=254,4 Ref=off

RT [min]	Type	Width [min]	Area	Height	Area%	Name
9.612	VB	0.25	31.16	8.05	0.34	
10.228	BV	0.26	9109.86	2017.10	99.37	
10.429	VB	0.14	26.81	5.68	0.29	
		**Sum**	9167.83		100.00	

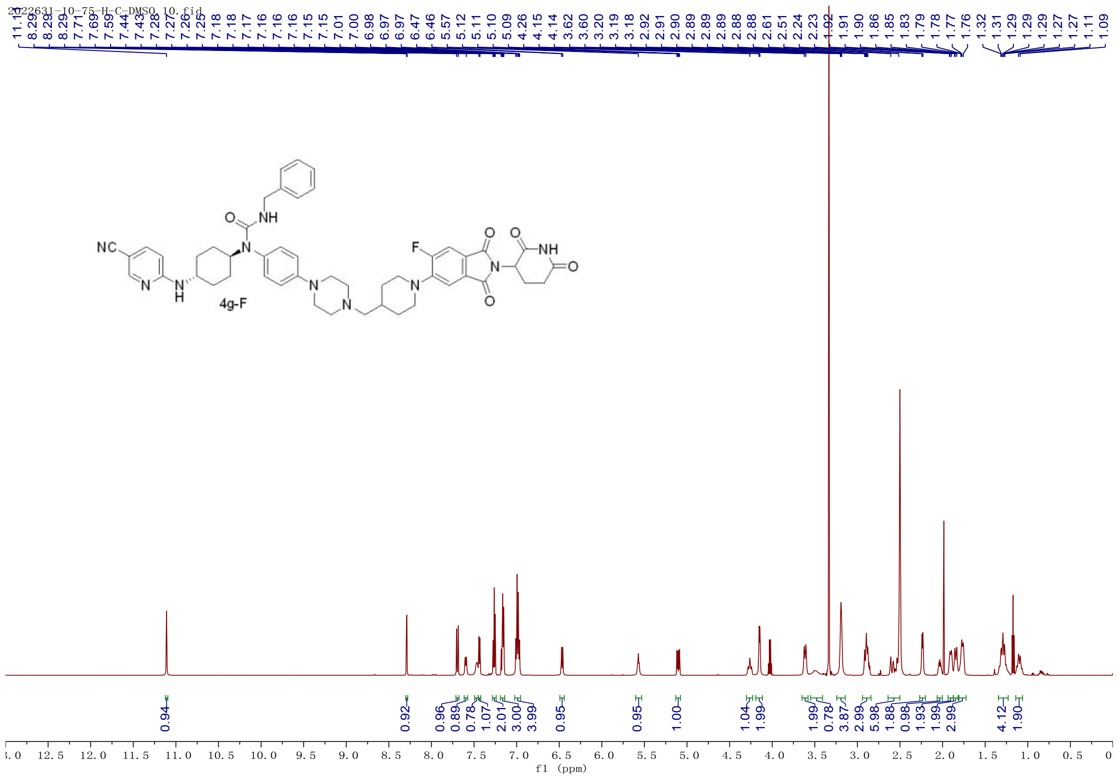
2022631-10-75-H-C-DMSO.10.fid
4g-F
f1 (ppm)

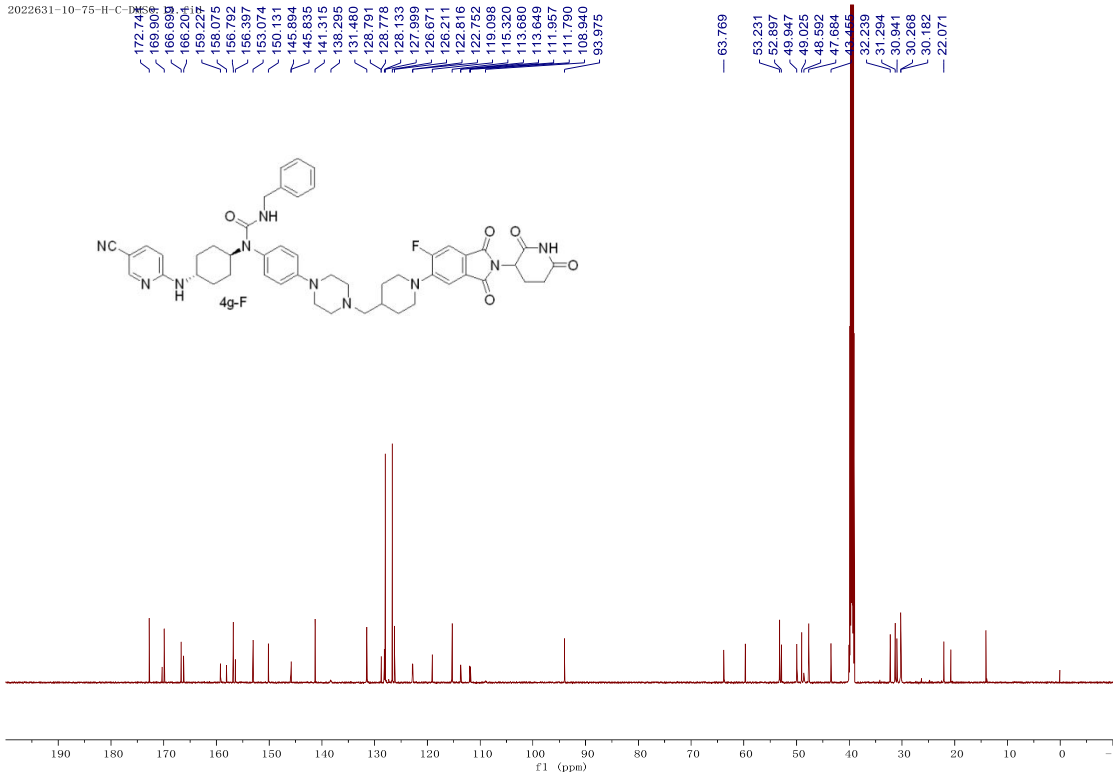
4g-F
f1 (ppm)

Mass Spectrum SmartFormula Report

Analysis Info

Analysis Name	D:\Data\SHUJVFENXI\DINGKE-GROUP\2022631-zlc-10-75_RA6_01_43816.d	Acquisition Date	4/24/2024 12:03:23 PM
Method	6min.m	Operator	BDAL@DE
Sample Name	2022631-zlc-10-75	Instrument / Ser#	maXis 4G 21240
Comment			

Acquisition Parameter

Source Type	ESI	Ion Polarity	Positive	Set Nebulizer	1.0 Bar
Focus	Not active	Set Capillary	3000 V	Set Dry Heater	220 °C
Scan Begin	50 m/z	Set End Plate Offset	-500 V	Set Dry Gas	6.0 l/min
Scan End	1500 m/z	Set Collision Cell RF	600.0 Vpp	Set Divert Valve	Waste

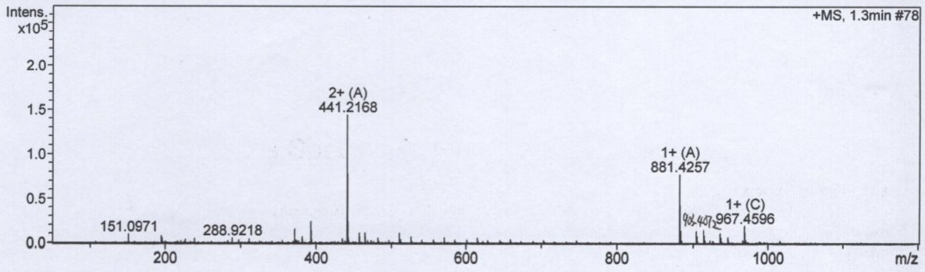

Meas. m/z	#	Formula	Score	m/z	err [ppm]	Mean err [ppm]	mSigma	rdb	e⁻ Conf	N-Rule
881.4257	1	C 49 H 54 F N 10 O 5	100.00	881.4257	0.0	0.5	10.7	27.5	even	ok
903.4072	1	C 49 H 53 F N 10 Na O 5	100.00	903.4077	0.5	0.7	9.6	27.5	even	ok

**Data file:**	zlc-10-75-10u.dx		
**Sequence Name:**	zlc-10-75-10u	**Project Name:**	1260
**Sample name:**	zlc-10-75-10u	**Operator:**	SYSTEM
**Instrument:**	1260	**Injection date:**	2024-07-01 16:34:50+08:00
**Inj. volume:**	10.000 µL	**Location:**	P1-F1
**Acq. method:**	normal.amx	**Type:**	Sample
**Processing method:**	GC_LC Area Percent_DefaultMethod.pmx	**Sample amount:**	0.00
**Manually modified:**	Manual Integration		

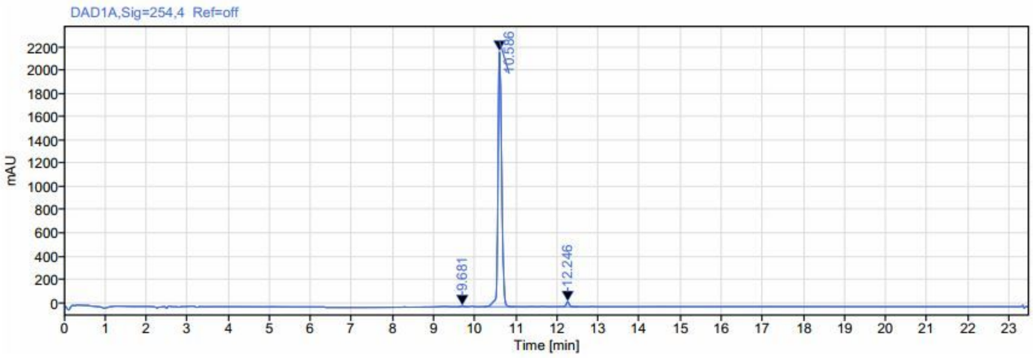

Signal: DAD1A,Sig=254,4 Ref=off

RT [min]	Type	Width [min]	Area	Height	Area%	Name
9.681	VB	0.26	54.25	10.00	0.39	
10.586	VV	0.75	13685.93	2198.04	98.10	
12.246	VB	0.37	211.05	43.70	1.51	
		**Sum**	13951.23		100.00	

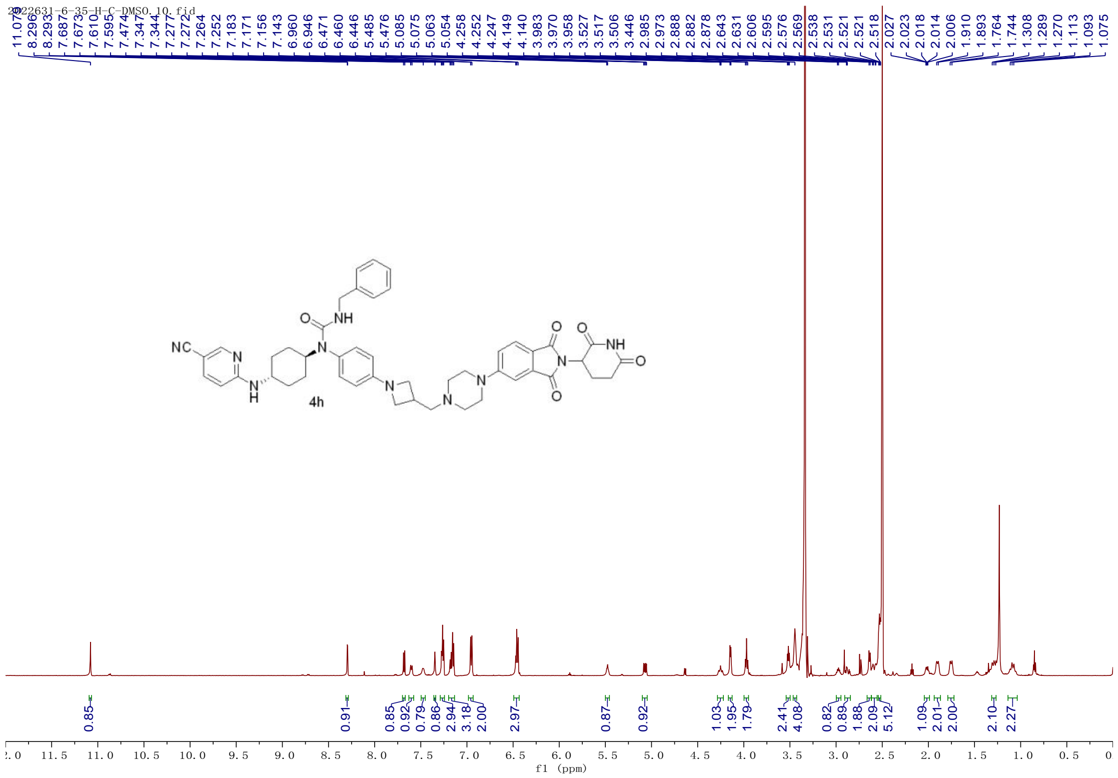
2022631-6-35-H-C-DMSO.10.fid
11.079
8.296
8.293
7.687
7.673
7.610
7.595
7.474
7.347
7.344
7.277
7.272
7.264
7.252
7.183
7.171
7.156
7.143
6.960
6.946
6.471
6.460
6.446
5.485
5.476
5.085
5.075
5.063
5.054
4.258
4.252
4.247
4.149
4.140
3.983
3.970
3.958
3.527
3.517
3.506
3.446
2.985
2.973
2.888
2.882
2.878
2.643
2.631
2.606
2.595
2.576
2.569
2.538
2.531
2.521
2.521
2.518
2.027
2.023
2.018
2.014
2.006
1.910
1.893
1.764
1.744
1.308
1.289
1.270
1.113
1.093
1.075
NC
N
NH
O
N
N
N
O
O
O
NH
O
N
H
4h
0.85
0.91
0.85
0.92
0.79
0.86
2.94
3.18
2.00
2.97
0.87
0.92
1.03
1.95
1.79
2.41
4.08
0.82
0.89
1.88
2.09
5.12
1.09
2.01
2.00
2.10
2.27
12.0 11.5 11.0 10.5 10.0 9.5 9.0 8.5 8.0 7.5 7.0 6.5 6.0 5.5 5.0 4.5 4.0 3.5 3.0 2.5 2.0 1.5 1.0 0.5 0
f1 (ppm)

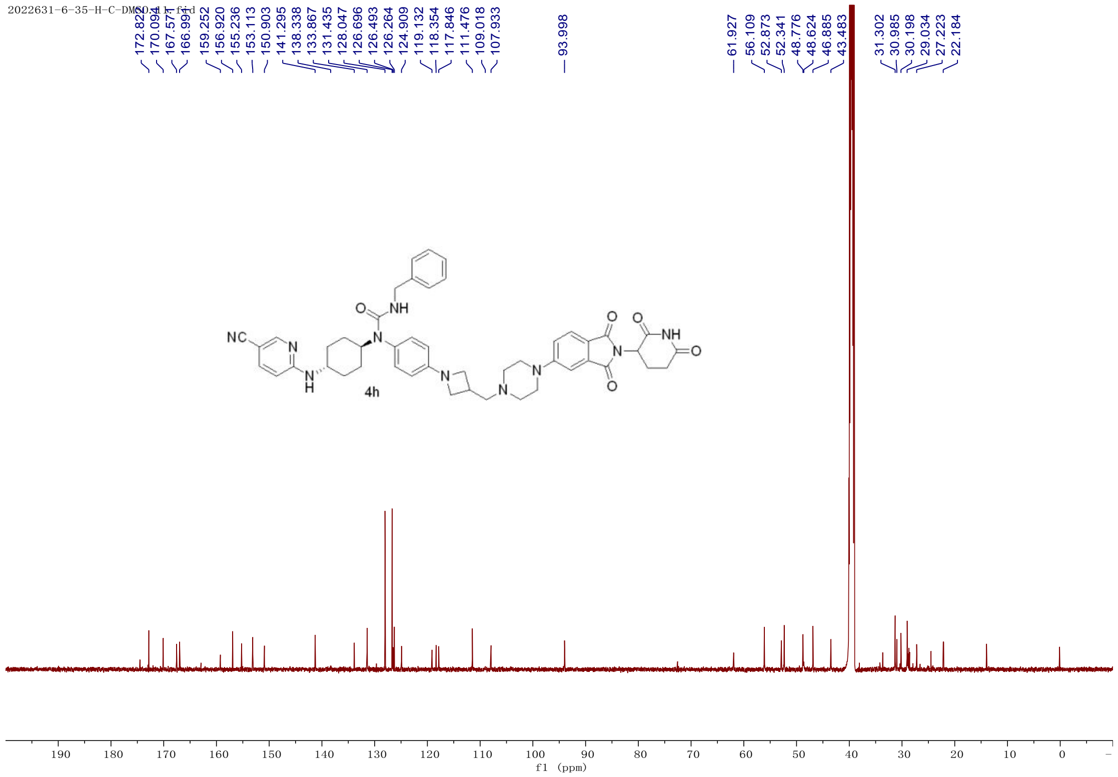
2022631-6-35-H-C-DMSO.11.fid
172.822
170.094
167.571
166.991
159.252
156.920
155.236
153.113
150.903
141.295
138.338
133.867
131.435
128.047
126.696
126.493
126.264
124.909
119.132
118.354
117.846
111.476
109.018
107.933
93.998
61.927
56.109
52.873
52.341
48.776
48.624
46.885
43.483
31.302
30.985
30.198
29.034
27.223
22.184
NC
N
NH
O
N
N
N
O
O
O
NH
O
N
H
4h
190 180 170 160 150 140 130 120 110 100 90 80 70 60 50 40 30 20 10 0
f1 (ppm)

Mass Spectrum SmartFormula Report

Analysis Info

Analysis Name	D:\Data\SHUJVFENXI\DINGKE-GROUP\2022631-ZLC-6-35_BA8_01_37545.d	Acquisition Date	6/12/2023 5:37:38 PM
Method	20150915.m	Operator	BDAL@DE
Sample Name	2022631-ZLC-6-35	Instrument / Ser#	maXis 4G 21240
Comment			

Acquisition Parameter

Source Type	ESI	Ion Polarity	Positive	Set Nebulizer	1.0 Bar
Focus	Not active	Set Capillary	3000 V	Set Dry Heater	220 °C
Scan Begin	50 m/z	Set End Plate Offset	-500 V	Set Dry Gas	6.0 l/min
Scan End	1500 m/z	Set Collision Cell RF	600.0 Vpp	Set Divert Valve	Waste

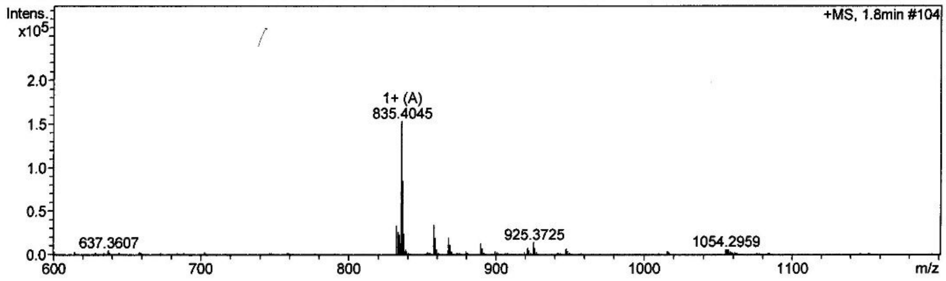

Meas. m/z	#	Formula	Score	m/z	err [ppm]	Mean err [ppm]	mSigma	rdb	e⁻ Conf	N-Rule
835.4045	1	C 47 H 51 N 10 O 5	100.00	835.4038	-0.8	-0.5	2.6	27.5	even	ok

**Data file:**	zlc-6-35.dx		
**Sequence Name:**	zlc-6-35	**Project Name:**	1260
**Sample name:**	zlc-6-35	**Operator:**	SYSTEM
**Instrument:**	1260	**Injection date:**	2023-06-05 18:20:14+08:00
**Inj. volume:**	10.000 µL	**Location:**	P1-F1
**Acq. method:**	normal.amx	**Type:**	Sample
**Processing method:**	GC_LC Area Percent_DefaultMethod.pmx	**Sample amount:**	0.00
**Manually modified:**	Manual Integration		

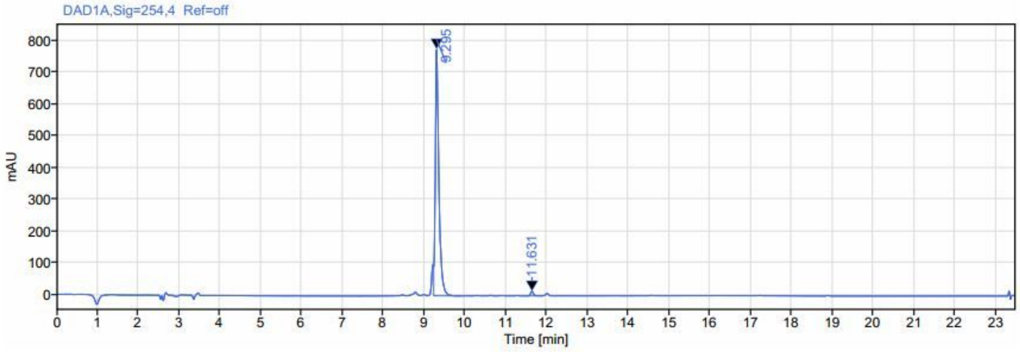

Signal: DAD1A,Sig=254,4 Ref=off

RT [min]	Type	Width [min]	Area	Height	Area%	Name
9.295	VB	0.64	4693.18	777.64	98.54	
11.631	BV	0.36	69.66	14.58	1.46	
		**Sum**	4762.84		100.00	

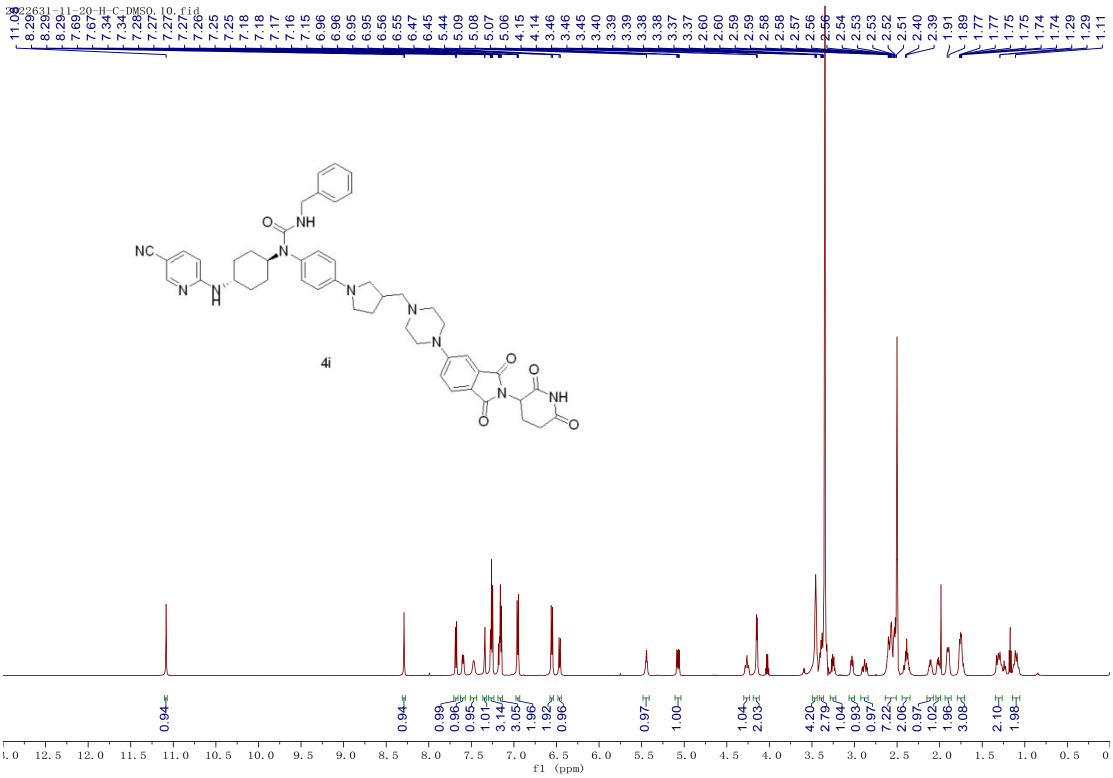
2022631-11-20-H-C-DMSO.10.fid
11.09 8.29 8.29 7.69 7.67 7.34 7.34 7.28 7.27 7.27 7.26 7.25 7.25 7.18 7.17 7.16 7.15 6.96 6.96 6.95 6.95 6.56 6.55 6.47 6.45 5.44 5.09 5.08 5.07 5.06 4.15 4.14 3.46 3.45 3.40 3.39 3.39 3.38 3.38 3.37 3.37 2.60 2.60 2.59 2.59 2.58 2.58 2.57 2.56 2.56 2.54 2.53 2.53 2.52 2.51 2.40 2.39 1.91 1.89 1.77 1.77 1.75 1.75 1.74 1.74 1.29 1.29 1.11
NC
NH
O
N
H
4i
NH
O
0.94 0.94 0.99 0.96 0.95 1.01 3.14 3.05 1.96 1.92 0.96 0.97 1.00 1.04 2.03 4.20 2.79 1.04 0.93 0.97 7.22 2.06 0.97 1.02 1.96 3.08 2.10 1.98
12.5 12.0 11.5 11.0 10.5 10.0 9.5 9.0 8.5 8.0 7.5 7.0 6.5 6.0 5.5 5.0 4.5 4.0 3.5 3.0 2.5 2.0 1.5 1.0 0.5 0
f1 (ppm)

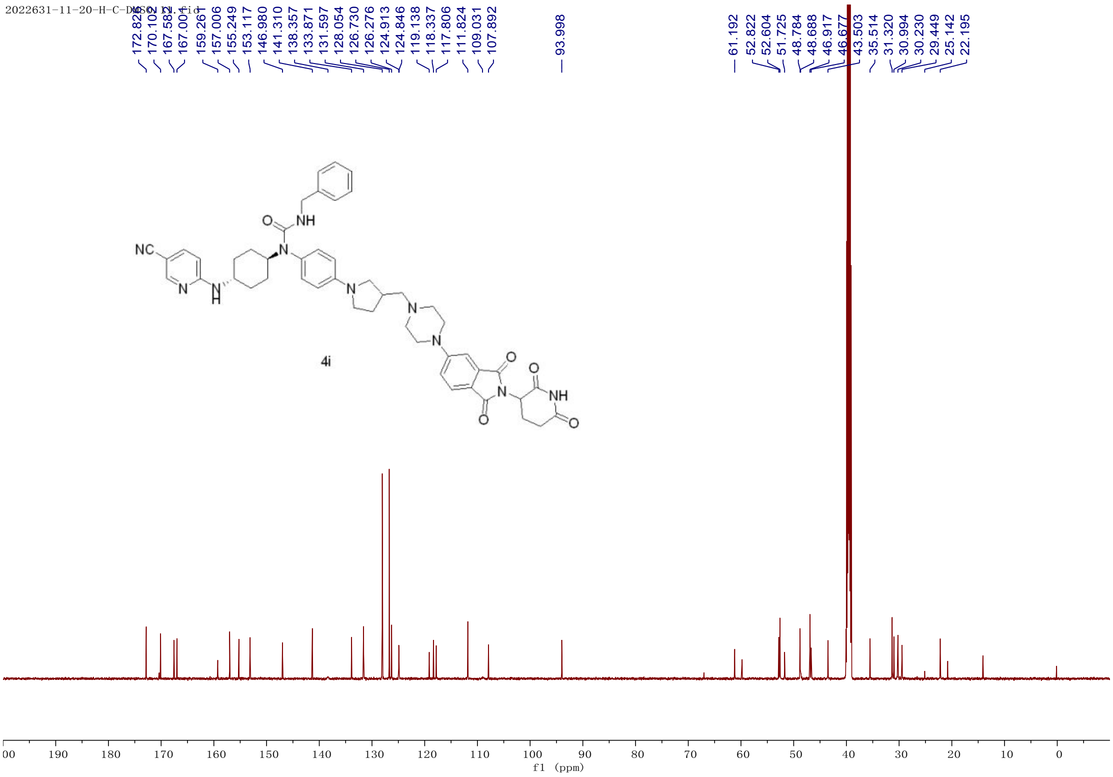
2022631-11-20-H-C-DMSO.11.fid
172.826 170.102 167.582 167.001 159.261 157.006 155.249 153.117 146.980 141.310 138.357 133.871 131.597 128.054 126.730 126.276 124.913 124.846 119.138 118.337 117.806 111.824 109.031 107.892
93.998
61.192 52.822 52.604 51.725 48.784 48.688 46.917 46.677 43.503 35.514 31.320 30.994 30.230 29.449 25.142 22.195
NC
NH
O
N
H
4i
NH
O
190 180 170 160 150 140 130 120 110 100 90 80 70 60 50 40 30 20 10 0
f1 (ppm)

Mass Spectrum SmartFormula Report

Analysis Info

Analysis Name	D:\Data\SHUJVFENXI\DINGKE-GROUP\2022631-zlc-11-20_RE2_01_45552.d	Acquisition Date	7/2/2024 12:32:48 PM
Method	6min.m	Operator	BDAL@DE
Sample Name	2022631-zlc-11-20	Instrument / Ser#	maXis 4G 21240
Comment			

Acquisition Parameter

Source Type	ESI	Ion Polarity	Positive	Set Nebulizer	1.0 Bar
Focus	Not active	Set Capillary	3000 V	Set Dry Heater	220 °C
Scan Begin	50 m/z	Set End Plate Offset	-500 V	Set Dry Gas	6.0 l/min
Scan End	1500 m/z	Set Collision Cell RF	600.0 Vpp	Set Divert Valve	Waste

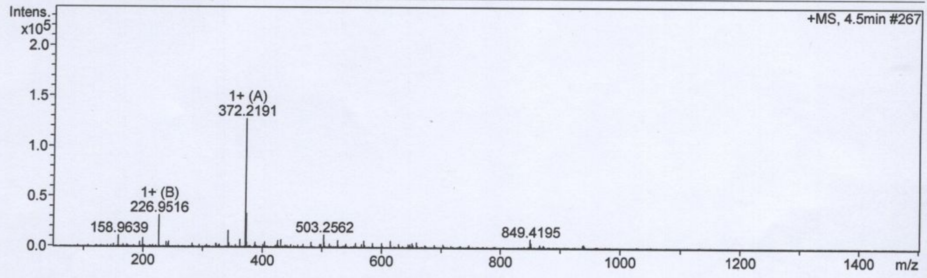

Meas. m/z	#	Formula	Score	m/z	err [ppm]	Mean err [ppm]	mSigma	rdb	e⁻ Conf	N-Rule
849.4195	1	C 48 H 53 N 10 O 5	100.00	849.4195	-0.1	-0.1	13.5	27.5	even	ok

**Data file:**	zlc-11-20-1.dx		
**Sequence Name:**	zlc-10-20	**Project Name:**	1260
**Sample name:**	zlc-11-20-1	**Operator:**	SYSTEM
**Instrument:**	1260	**Injection date:**	2024-07-01 19:35:47+08:00
**Inj. volume:**	10.000 µL	**Location:**	P1-F2
**Acq. method:**	normal.amx	**Type:**	Sample
**Processing method:**	GC_LC Area Percent_DefaultMethod.pmx	**Sample amount:**	0.00
**Manually modified:**	Manual Integration		

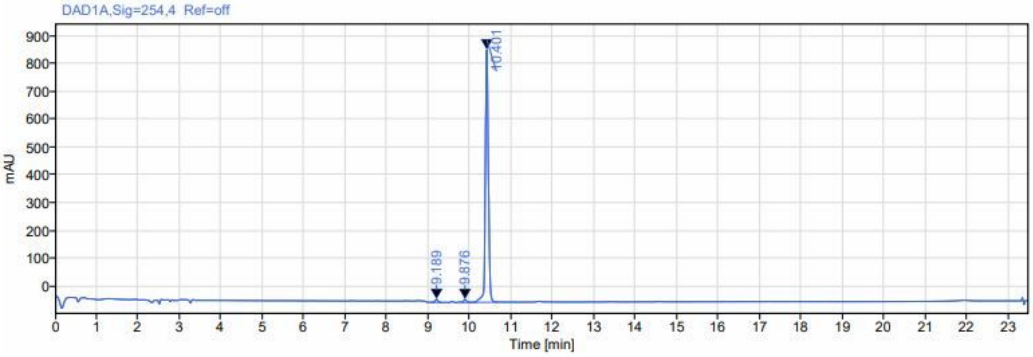

Signal: DAD1A,Sig=254,4 Ref=off

RT [min]	Type	Width [min]	Area	Height	Area%	Name
9.189	BB	0.27	45.53	10.57	0.96	
9.876	VB	0.29	51.14	10.57	1.08	
10.401	BV	0.57	4655.52	909.85	97.97	
		**Sum**	4752.19		100.00	

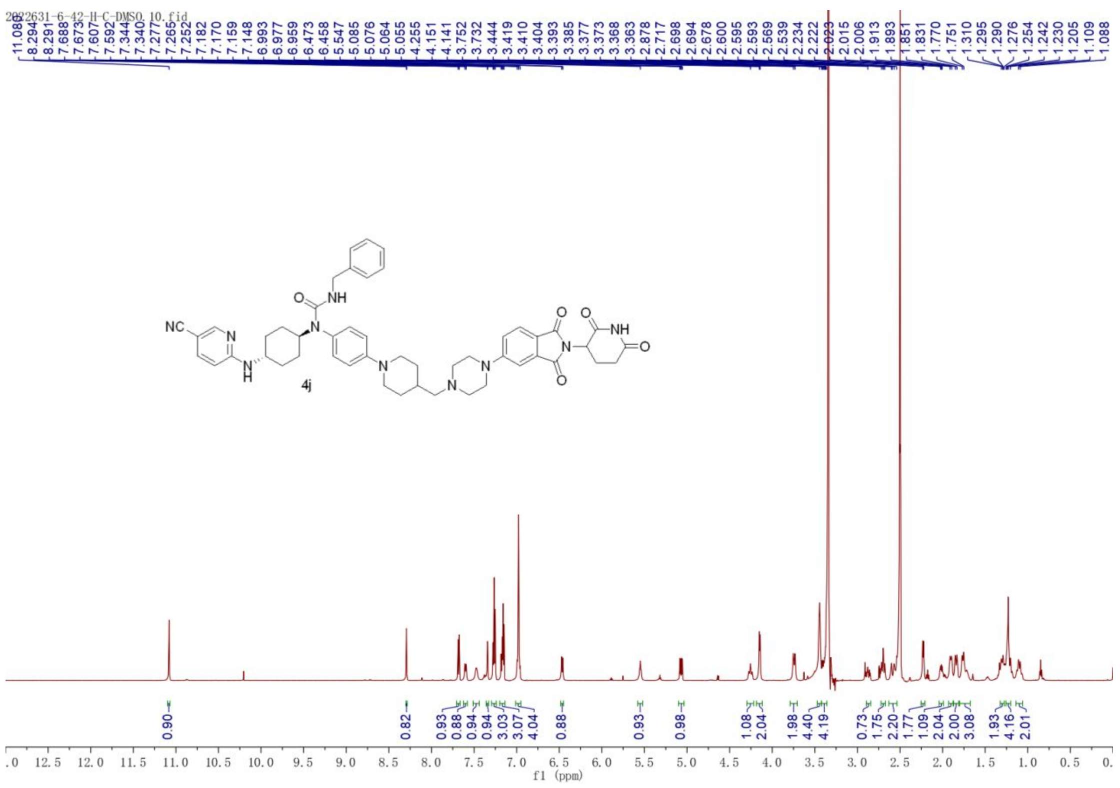
2022631-6-42-H-C-DMSO.10.fid
4j
f1 (ppm)

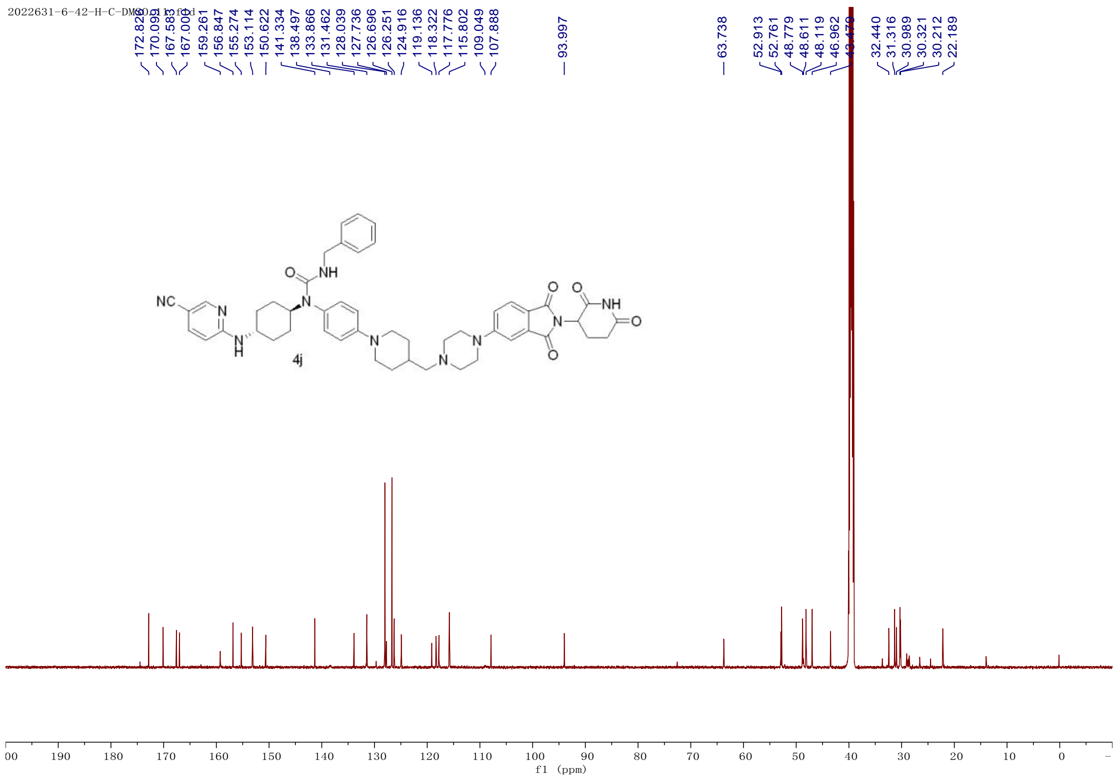
4j
f1 (ppm)

Mass Spectrum SmartFormula Report

Analysis Info

Analysis Name	D:\Data\SHUJVFENXI\DINGKE-GROUP\2022631-ZLC-6-42_BB2_01_37527.d	Acquisition Date	6/12/2023 4:01:39 PM
Method	20150915.m	Operator	BDAL@DE
Sample Name	2022631-ZLC-6-42	Instrument / Ser#	maXis 4G 21240
Comment			

Acquisition Parameter

Source Type	ESI	Ion Polarity	Positive	Set Nebulizer	1.0 Bar
Focus	Not active	Set Capillary	3000 V	Set Dry Heater	220 °C
Scan Begin	50 m/z	Set End Plate Offset	-500 V	Set Dry Gas	6.0 l/min
Scan End	1500 m/z	Set Collision Cell RF	600.0 Vpp	Set Divert Valve	Waste

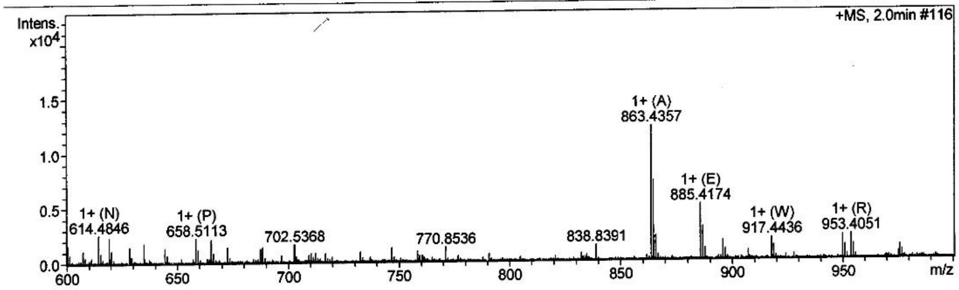

Meas. m/z	#	Formula	Score	m/z	err [ppm]	Mean err [ppm]	mSigma	rdb	e⁻ Conf	N-Rule
863.4357	1	C 49 H 55 N 10 O 5	100.00	863.4351	-0.7	-0.7	12.1	27.5	even	ok

**Data file:**	zlc-6-42.dx		
**Sequence Name:**	zlc-6-42	**Project Name:**	1260
**Sample name:**	zlc-6-42	**Operator:**	SYSTEM
**Instrument:**	1260	**Injection date:**	2023-06-01 20:40:00+08:00
**Inj. volume:**	10.000 µL	**Location:**	P1-F2
**Acq. method:**	normal.amx	**Type:**	Sample
**Processing method:**	GC_LC Area Percent_DefaultMethod.pmx	**Sample amount:**	0.00
**Manually modified:**	Manual Integration		

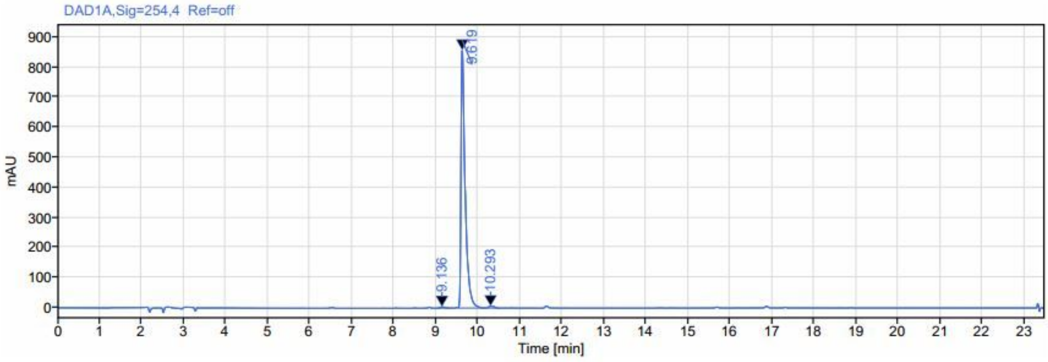

Signal: DAD1A,Sig=254,4 Ref=off

RT [min]	Type	Width [min]	Area	Height	Area%	Name
9.136	VB	0.31	35.09	4.74	0.58	
9.619	VB	0.65	5904.13	859.41	98.40	
10.293	BB	0.33	61.15	6.37	1.02	
		Sum	6000.37		100.00	

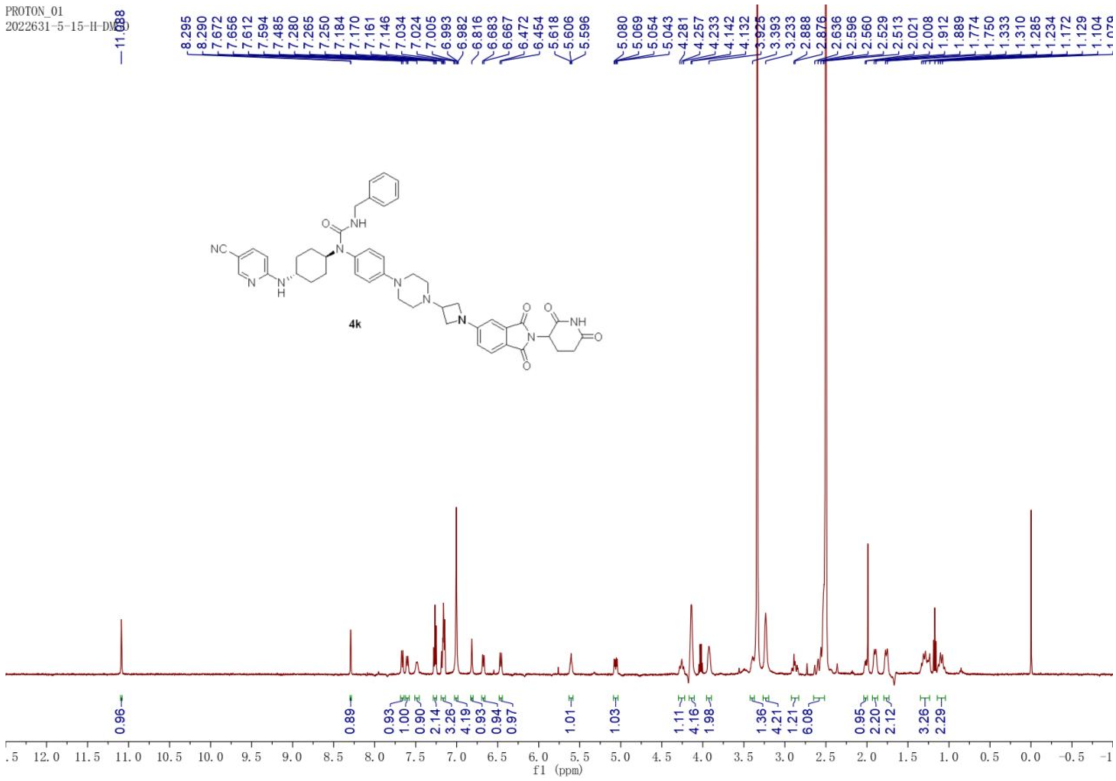
PROTON_01
4k
f1 (ppm)

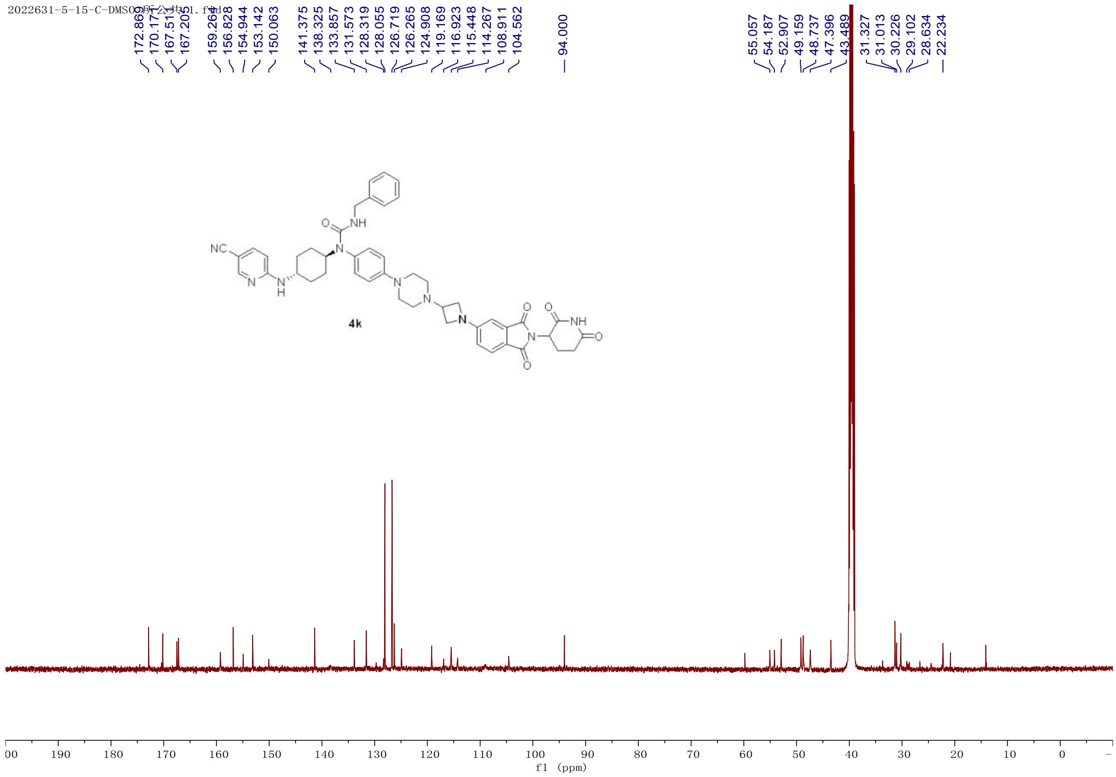
4k
f1 (ppm)

Mass Spectrum SmartFormula Report

Analysis Info			
Analysis Name	D:\Data\SHUJVFENXI\DINGKE-GROUP\2022631-ZLC-5-15_BA8_01_35730.d	Acquisition Date	3/21/2023 10:00:10 PM
Method	20150915.m	Operator	BDAL@DE
Sample Name	2022631-ZLC-5-15	Instrument / Ser#	maXis 4G 21240
Comment			

Acquisition Parameter

Source Type	ESI	Ion Polarity	Positive	Set Nebulizer	1.0 Bar
Focus	Not active	Set Capillary	3000 V	Set Dry Heater	220 °C
Scan Begin	50 m/z	Set End Plate Offset	-500 V	Set Dry Gas	6.0 l/min
Scan End	1500 m/z	Set Collision Cell RF	600.0 Vpp	Set Divert Valve	Waste

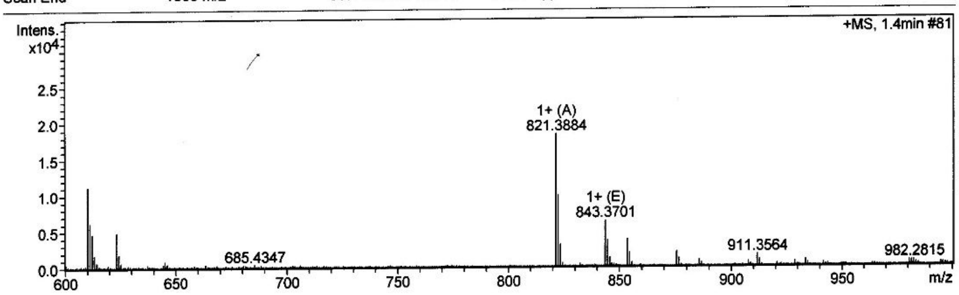

Meas. m/z	#	Formula	Score	m/z	err [ppm]	Mean err [ppm]	mSigma	rdb	e⁻ Conf	N-Rule
821.3884	1	C 46 H 49 N 10 O 5	100.00	821.3882	-0.2	0.3	9.1	27.5	even	ok

**Data file:**	zlc-5-15.dx		
**Sequence Name:**	SingleSample	**Project Name:**	1260
**Sample name:**	zlc-5-15	**Operator:**	SYSTEM
**Instrument:**	1260	**Injection date:**	2023-03-14 19:31:47+08:00
**Inj. volume:**	10.000 µL	**Location:**	P1-B1
**Acq. method:**	normal.amx	**Type:**	Sample
**Processing method:**	GC_LC 面积百分比_DefaultMethod.pmx	**Sample amount:**	0.00
**Manually modified:**	Manual Integration		

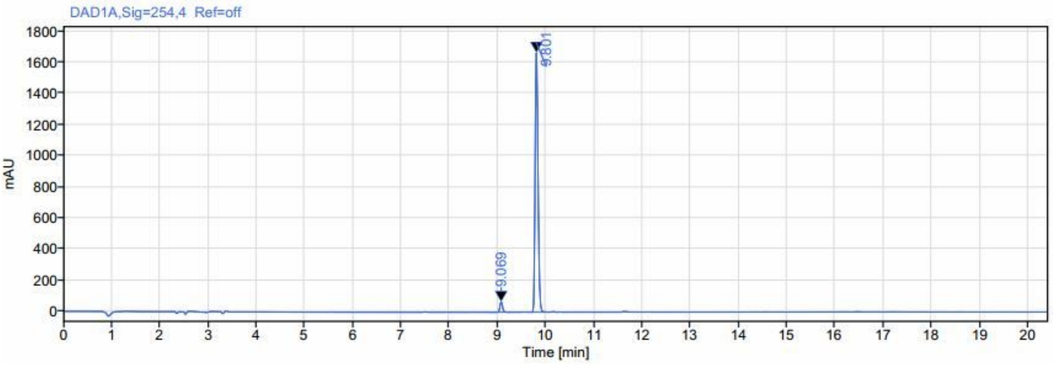

Signal: DAD1A,Sig=254,4 Ref=off

RT [min]	Type	Width [min]	Area	Height	Area%	Name
9.069	BB	0.46	249.75	65.92	3.46	
9.801	VV	0.38	6962.60	1668.78	96.54	
		**Sum**	7212.36		100.00	

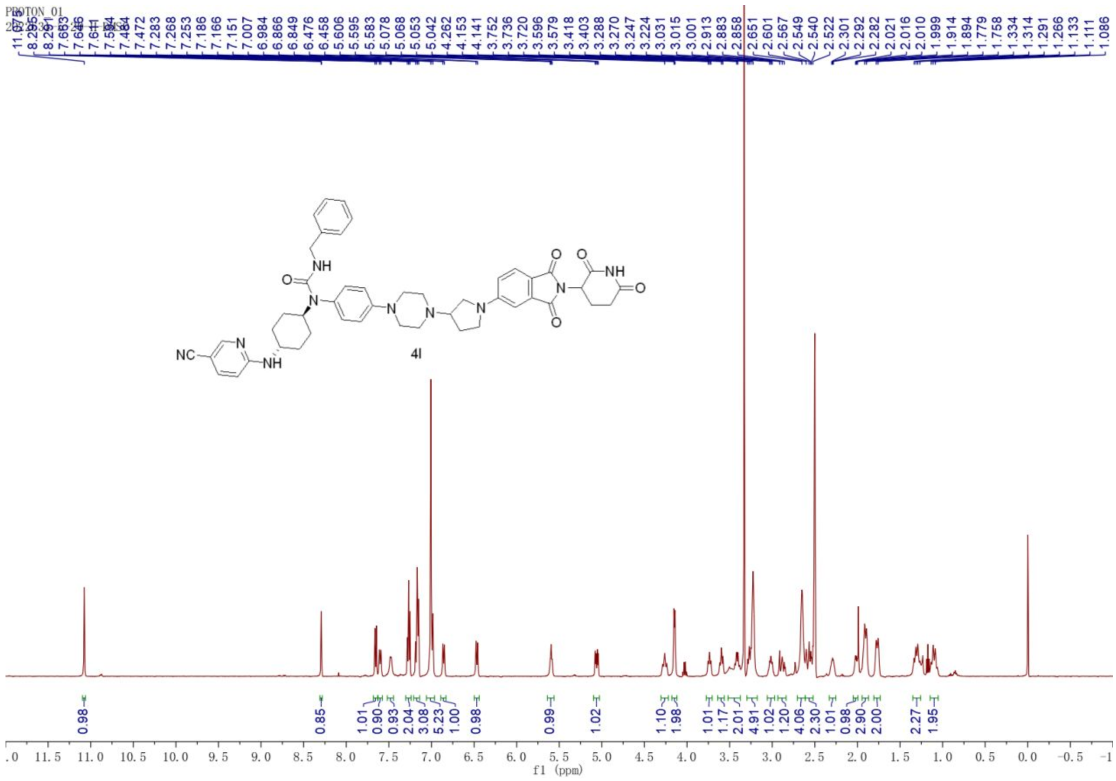
PROTON_01
11.087 8.295 8.291 7.653 7.636 7.611 7.594 7.484 7.472 7.283 7.268 7.253 7.186 7.166 7.151 7.007 6.984 6.866 6.849 6.476 6.458 5.606 5.595 5.583 5.078 5.068 5.053 5.042 4.262 4.153 4.141 3.752 3.736 3.720 3.596 3.579 3.418 3.403 3.288 3.270 3.247 3.224 3.031 3.015 3.001 2.913 2.883 2.858 2.651 2.601 2.567 2.549 2.540 2.522 2.301 2.292 2.282 2.021 2.016 2.010 1.999 1.914 1.894 1.779 1.758 1.334 1.314 1.291 1.266 1.133 1.111 1.086
NH
O
N
N
N
N
N
O
O
N
NH
O
O
NC
N
NH
4l
0.98 0.85 1.01 0.90 0.93 2.04 3.08 5.23 1.00 0.98 0.99 1.02 1.10 1.98 1.01 1.17 2.01 4.91 1.02 1.20 4.06 2.30 1.01 0.98 2.90 2.00 2.27 1.95
f1 (ppm)

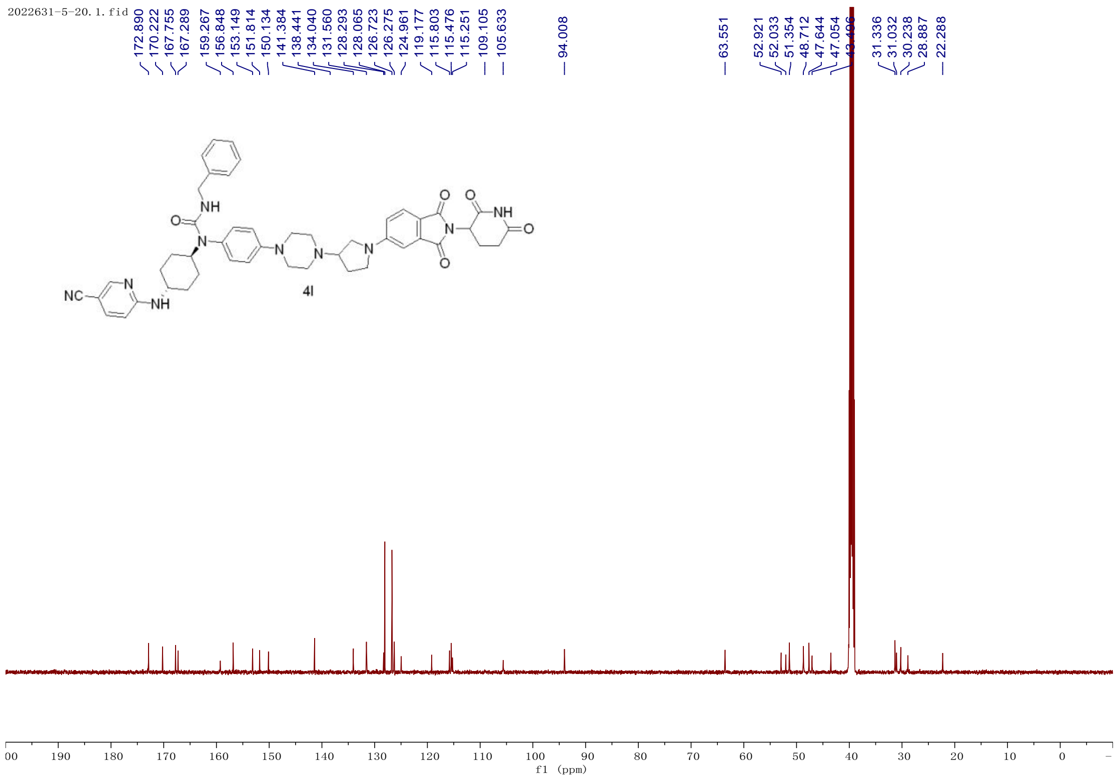
2022631-5-20.1.fid
172.890 170.222 167.755 167.289 159.267 156.848 153.149 151.814 150.134 141.384 138.441 134.040 131.560 128.293 128.065 126.723 126.275 124.961 119.177 115.803 115.476 115.251 109.105 105.633 94.008 63.551 52.921 52.033 51.354 48.712 47.644 47.054 43.406 31.336 31.032 30.238 28.887 22.288
NH
O
N
N
N
N
N
O
O
N
NH
O
O
NC
N
NH
4l
f1 (ppm)

Analysis Info

Analysis Name	D:\Data\SHUJVFENXI\DINGKE-GROUP\2022631-ZLC-5-20_BA6_01_35728.d	Acquisition Date	3/21/2023 9:49:09 PM
Method	20150915.m	Operator	BDAL@DE
Sample Name	2022631-ZLC-5-20	Instrument / Ser#	maXis 4G 21240
Comment			

Acquisition Parameter

Source Type	ESI	Ion Polarity	Positive	Set Nebulizer	1.0 Bar
Focus	Not active	Set Capillary	3000 V	Set Dry Heater	220 °C
Scan Begin	50 m/z	Set End Plate Offset	-500 V	Set Dry Gas	6.0 l/min
Scan End	1500 m/z	Set Collision Cell RF	600.0 Vpp	Set Divert Valve	Waste

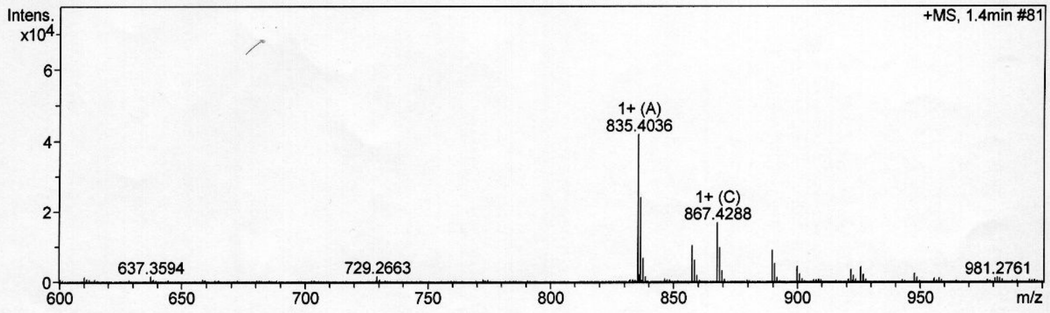

Meas. m/z	#	Formula	Score	m/z	err [ppm]	Mean err [ppm]	mSigma	rdb	e⁻ Conf	N-Rule
835.4036	1	C 47 H 51 N 10 O 5	100.00	835.4038	0.3	1.1	12.8	27.5	even	ok

**Data file:**	zlc-5-20-1.dx		
**Sequence Name:**	SingleSample	**Project Name:**	1260
**Sample name:**	zlc-5-20-1	**Operator:**	SYSTEM
**Instrument:**	1260	**Injection date:**	2023-03-19 19:36:10+08:00
**Inj. volume:**	10.000 µL	**Location:**	P1-E1
**Acq. method:**	normal.amx	**Type:**	Sample
**Processing method:**	GC_LC 面积百分比_DefaultMethod.pmx	**Sample amount:**	0.00
**Manually modified:**	Manual Integration		

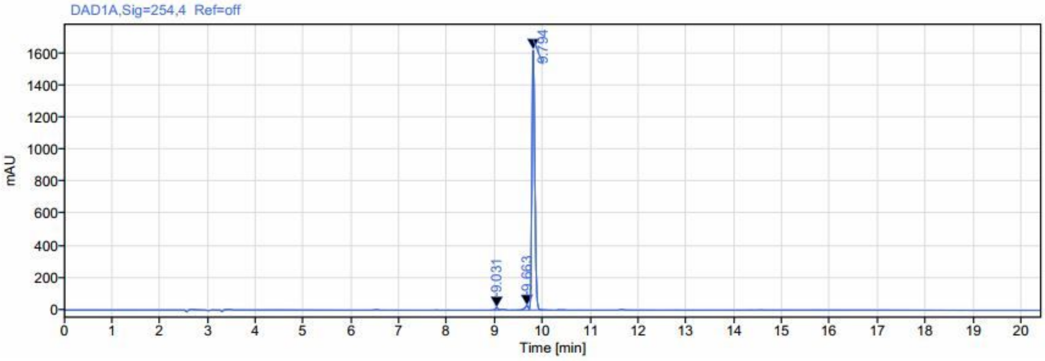

Signal: DAD1A,Sig=254,4 Ref=off

RT [min]	Type	Width [min]	Area	Height	Area%	Name
9.031	BV	0.13	60.58	16.54	0.86	
9.663	BV	0.29	147.58	28.39	2.10	
9.794	VB	0.29	6815.24	1622.30	97.04	
		**Sum**	7023.40		100.00	

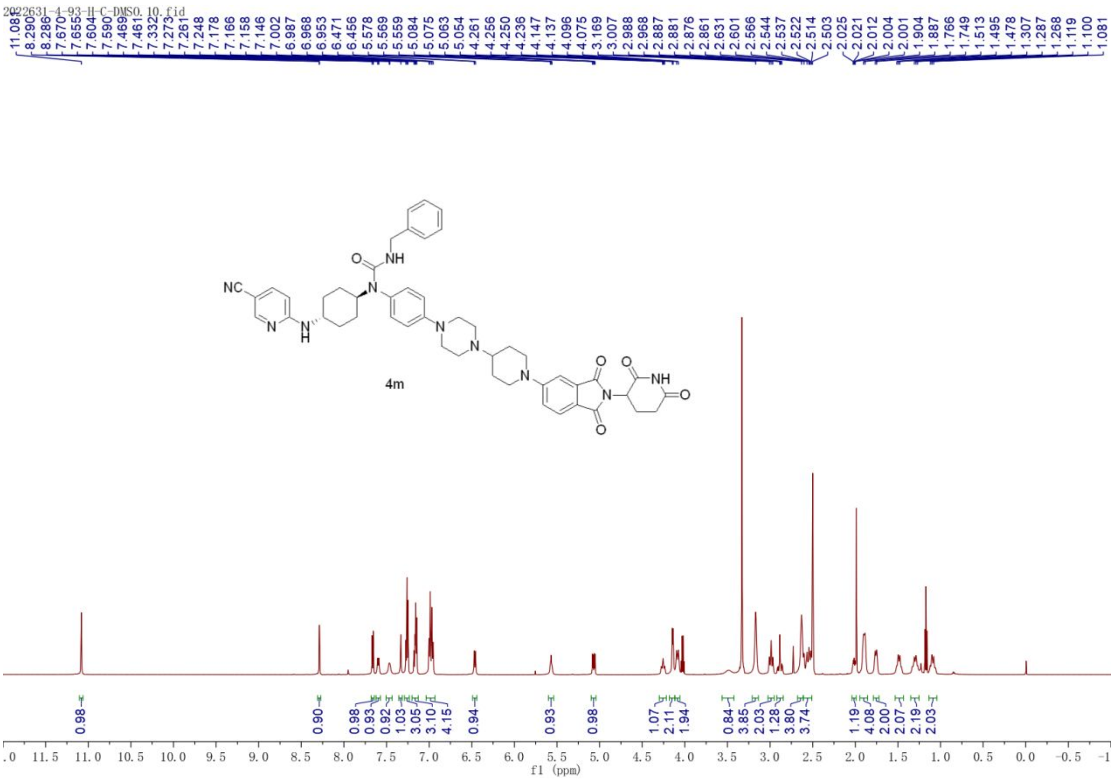
2022631-4-93-H-C-DMSO.10.fid
11.085 8.290 8.286 7.670 7.655 7.604 7.590 7.469 7.461 7.332 7.273 7.261 7.248 7.178 7.166 7.158 7.146 7.002 6.987 6.968 6.953 6.471 6.456 5.578 5.569 5.559 5.084 5.075 5.063 5.054 4.261 4.256 4.250 4.236 4.147 4.096 4.075 3.169 3.007 2.988 2.968 2.887 2.881 2.876 2.861 2.631 2.601 2.566 2.544 2.537 2.522 2.514 2.503 2.025 2.021 2.012 2.004 2.001 1.904 1.887 1.766 1.749 1.513 1.495 1.478 1.307 1.287 1.268 1.119 1.100 1.081
NC
NH
O
N
H
4m
O
NH
O
0.98 0.90 0.98 0.93 0.92 1.03 3.05 3.10 4.15 0.94 0.93 0.98 1.07 2.11 1.94 0.84 3.85 2.03 1.28 3.80 3.74 1.19 4.08 2.00 2.07 2.19 2.03
f1 (ppm)

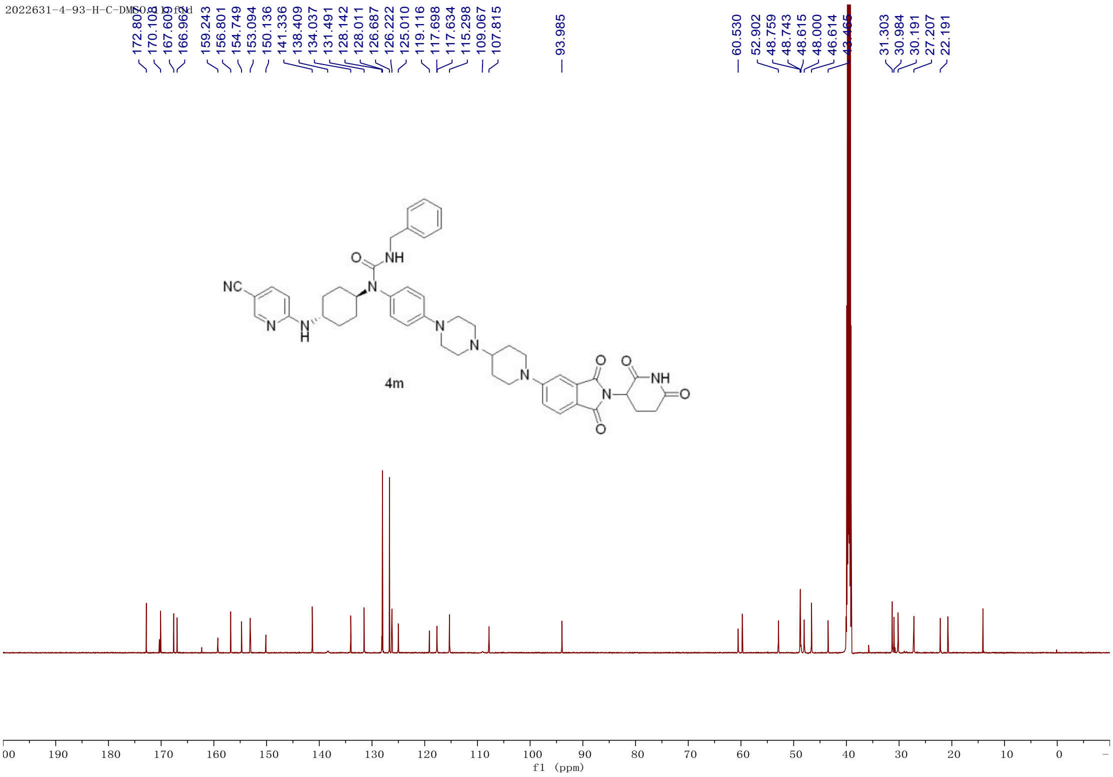
172.803 170.102 167.609 166.962 159.243 156.801 154.749 153.094 150.136 141.336 138.409 134.037 131.491 128.142 128.011 126.687 126.222 125.010 119.116 117.698 117.634 115.298 109.067 107.815 93.985 60.530 52.902 48.759 48.743 48.615 48.000 46.614 31.303 30.984 30.191 27.207 22.191
NC
NH
O
N
H
4m
O
NH
O
f1 (ppm)

Mass Spectrum SmartFormula Report

Analysis Info

Analysis Name	D:\Data\SHUJVFENXI\DINGKE-GROUP\2022631-ZLC-4-93_RC6_01_34697.d	Acquisition Date	2/27/2023 5:07:07 PM
Method	20150915.m	Operator	BDAL@DE
Sample Name	2022631-ZLC-4-93	Instrument / Ser#	maXis 4G 21240
Comment			

Acquisition Parameter

Source Type	ESI	Ion Polarity	Positive	Set Nebulizer	1.0 Bar
Focus	Not active	Set Capillary	3000 V	Set Dry Heater	220 °C
Scan Begin	50 m/z	Set End Plate Offset	-500 V	Set Dry Gas	6.0 l/min
Scan End	1500 m/z	Set Collision Cell RF	600.0 Vpp	Set Divert Valve	Waste

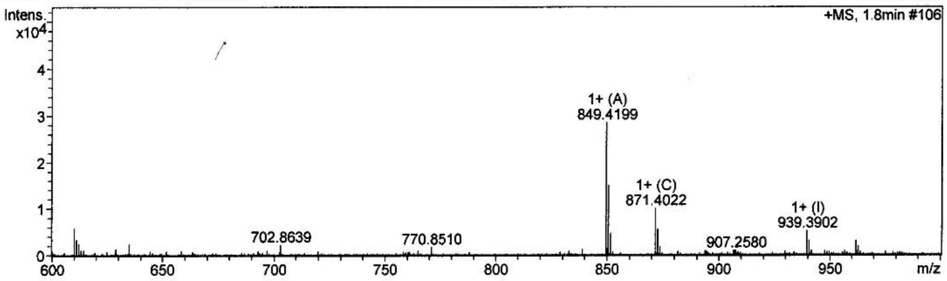

Meas. m/z	#	Formula	Score	m/z	err [ppm]	Mean err [ppm]	mSigma	rdb	e⁻ Conf	N-Rule
849.4199	1	C 48 H 53 N 10 O 5	100.00	849.4195	-0.4	0.1	17.3	27.5	even	ok
871.4022	1	C 48 H 52 N 10 Na O 5	100.00	871.4014	-0.8	-0.4	19.6	27.5	even	ok

**Data file:**	zlc-4-93.dx		
**Sequence Name:**	zlc-4-93	**Project Name:**	1260
**Sample name:**	zlc-4-93	**Operator:**	SYSTEM
**Instrument:**	1260	**Injection date:**	2023-02-22 09:50:32+08:00
**Inj. volume:**	10.000 µL	**Location:**	P1-B2
**Acq. method:**	normal.amx	**Type:**	Sample
**Processing method:**	GC_LC Area Percent_DefaultMethod.pmx	**Sample amount:**	0.00
**Manually modified:**	Manual Integration		

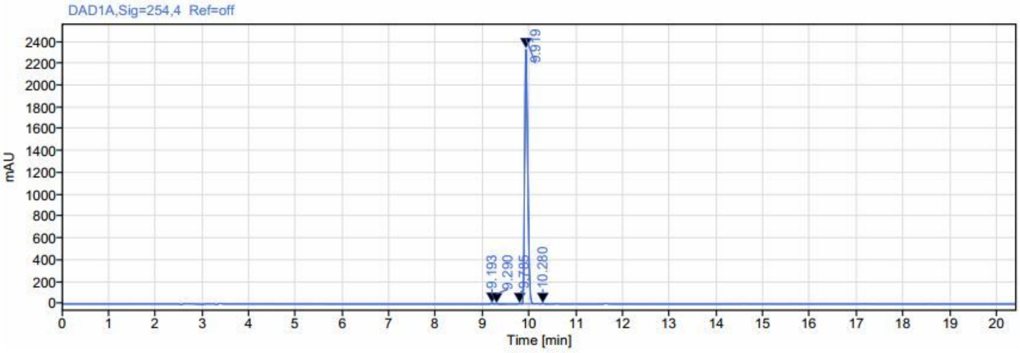

Signal: DAD1A,Sig=254,4 Ref=off

RT [min]	Type	Width [min]	Area	Height	Area%	Name
9.193	VV	0.13	35.55	9.18	0.33	
9.290	VB	0.23	20.50	4.46	0.19	
9.785	BV	0.13	22.10	5.86	0.20	
9.919	VV	0.28	10832.19	2341.30	99.08	
10.280	BB	0.17	22.45	5.64	0.21	
		**Sum**	10932.79		100.00	

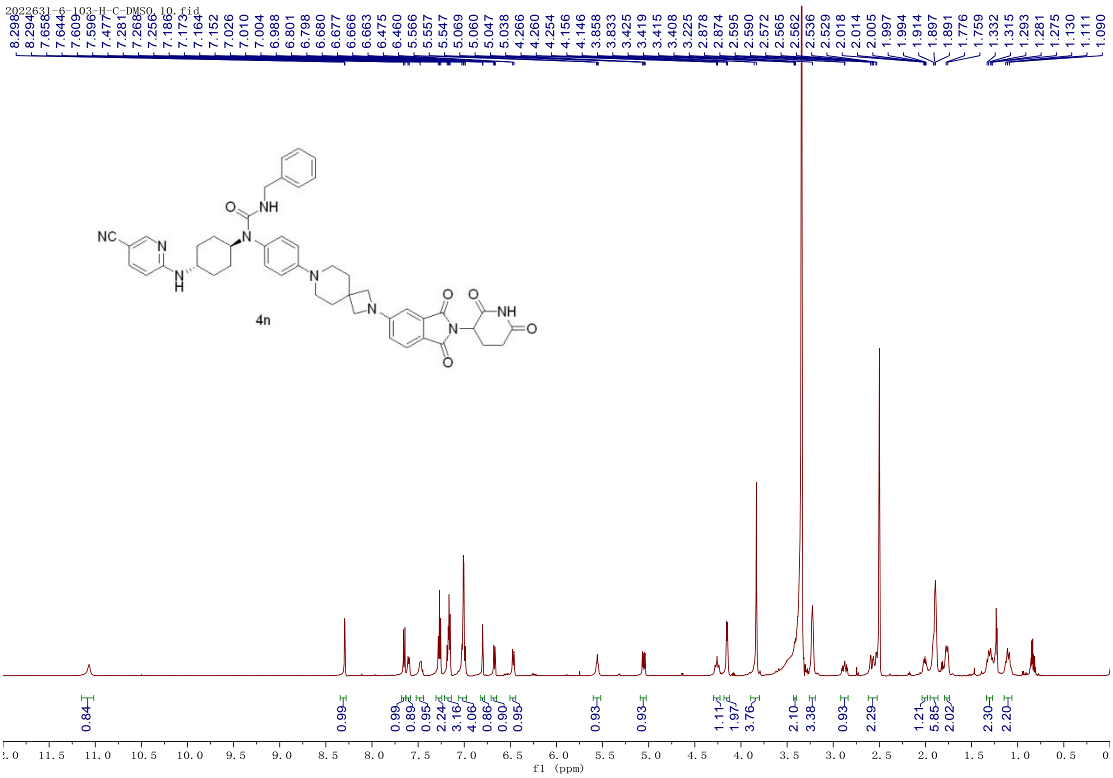
2022631-6-103-H-C-DMSO.10.fid
8.298 8.294 7.658 7.644 7.609 7.596 7.477 7.281 7.268 7.256 7.186 7.173 7.164 7.152 7.026 7.010 7.004 6.988 6.801 6.798 6.680 6.677 6.666 6.663 6.475 6.460 5.566 5.557 5.547 5.069 5.060 5.047 5.038 4.266 4.260 4.254 4.156 4.146 3.858 3.833 3.425 3.419 3.415 3.408 3.225 2.878 2.874 2.595 2.590 2.572 2.565 2.562 2.536 2.529 2.018 2.014 2.005 1.997 1.994 1.914 1.897 1.891 1.776 1.759 1.332 1.315 1.293 1.281 1.275 1.130 1.111 1.090
NC
N
NH
O
N
H
N
N
O
O
N
O
NH
O
4n
0.84 0.99 0.99 0.89 0.95 2.24 3.16 4.06 0.86 0.90 0.95 0.93 0.93 1.11 1.97 3.76 2.10 3.38 0.93 2.29 1.21 5.85 2.02 2.30 2.20
f1 (ppm)

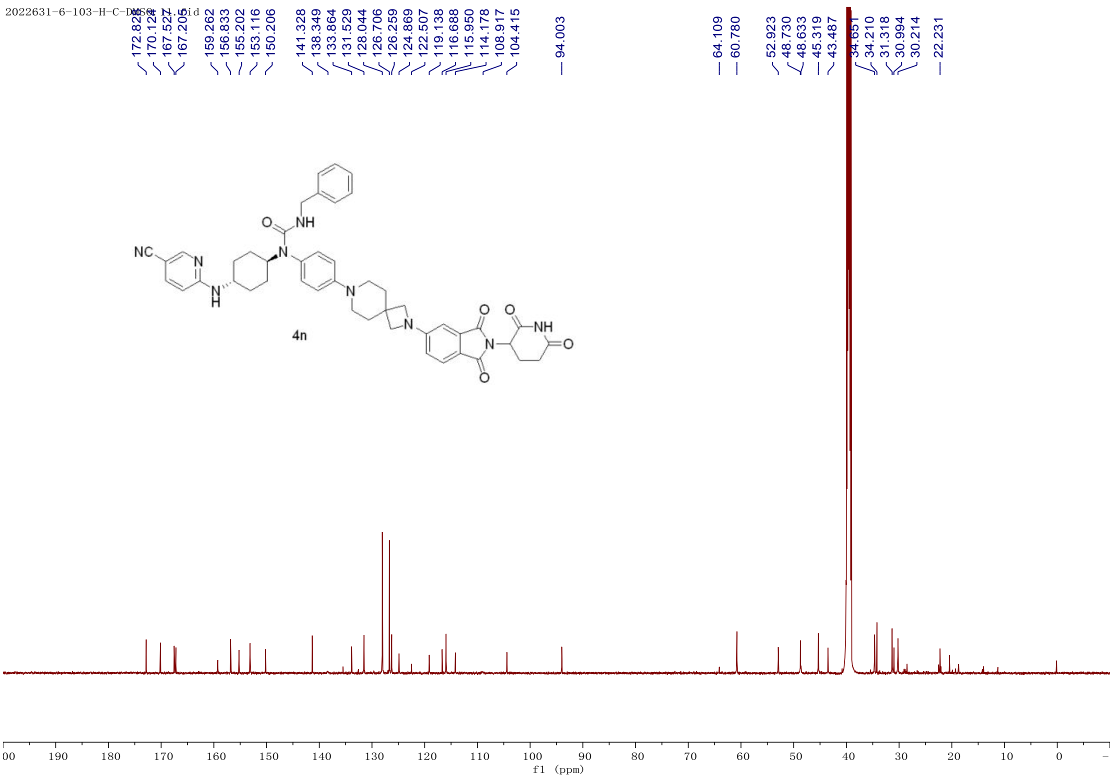
2022631-6-103-H-C-DMSO.fid
172.828 170.124 167.527 167.205 159.262 156.833 155.202 153.116 150.206 141.328 138.349 133.864 131.529 128.044 126.706 126.259 124.869 122.507 119.138 116.688 115.950 114.178 108.917 104.415 94.003 64.109 60.780 52.923 48.730 48.633 45.319 43.487 34.651 34.210 31.318 30.994 30.214 22.231
NC
N
NH
O
N
H
N
N
O
O
N
O
NH
O
4n
f1 (ppm)

Mass Spectrum SmartFormula Report

Analysis Info

Analysis Name	D:\Data\SHUJVFENXI\DINGKE-GROUP\2022631-ZLC-6-103_BA3_01_37892.d	Acquisition Date	6/30/2023 12:36:47 PM
Method	20150915.m	Operator	BDAL@DE
Sample Name	2022631-ZLC-6-103	Instrument / Ser#	maXis 4G 21240
Comment			

Acquisition Parameter

Source Type	ESI	Ion Polarity	Positive	Set Nebulizer	1.0 Bar
Focus	Not active	Set Capillary	3000 V	Set Dry Heater	220 °C
Scan Begin	50 m/z	Set End Plate Offset	-500 V	Set Dry Gas	6.0 l/min
Scan End	1500 m/z	Set Collision Cell RF	600.0 Vpp	Set Divert Valve	Waste

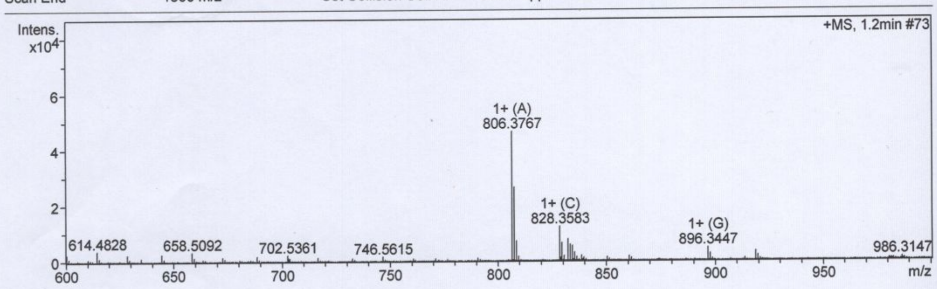

Meas. m/z	#	Formula	Score	m/z	err [ppm]	Mean err [ppm]	mSigma	rdb	e⁻ Conf	N-Rule
806.3767	1	C 46 H 48 N 9 O 5	100.00	806.3773	0.7	1.0	17.0	27.5	even	ok
828.3583	1	C 46 H 47 N 9 Na O 5	100.00	828.3592	1.2	1.5	4.0	27.5	even	ok

**Data file:**	zlc-6-103.dx		
**Sequence Name:**	zlc-6-101-6-102-6-103	**Project Name:**	1260
**Sample name:**	zlc-6-103	**Operator:**	SYSTEM
**Instrument:**	1260	**Injection date:**	2023-06-28 16:47:33+08:00
**Inj. volume:**	10.000 µL	**Location:**	P1-F4
**Acq. method:**	normal.amx	**Type:**	Sample
**Processing method:**	GC_LC Area Percent_DefaultMethod.pmx	**Sample amount:**	0.00
**Manually modified:**	Manual Integration		

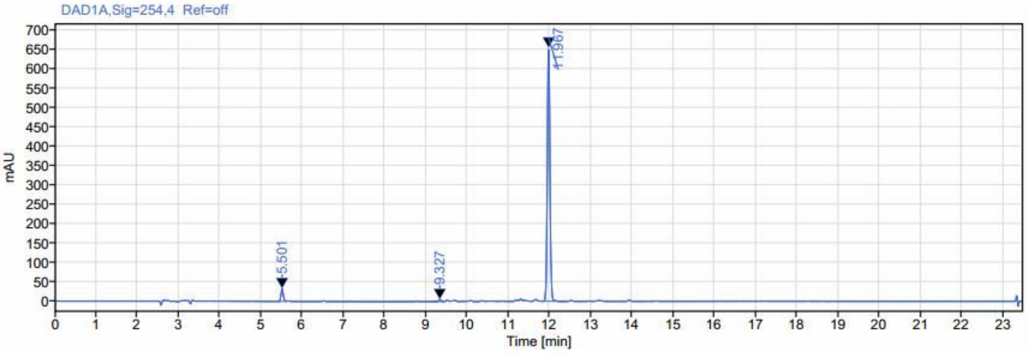

Signal: DAD1A,Sig=254,4 Ref=off

RT [min]	Type	Width [min]	Area	Height	Area%	Name
5.501	BV	0.21	129.07	33.06	4.21	
9.327	BV	0.27	22.20	5.41	0.72	
11.967	BB	0.57	2914.88	654.50	95.07	
		**Sum**	3066.15		100.00	

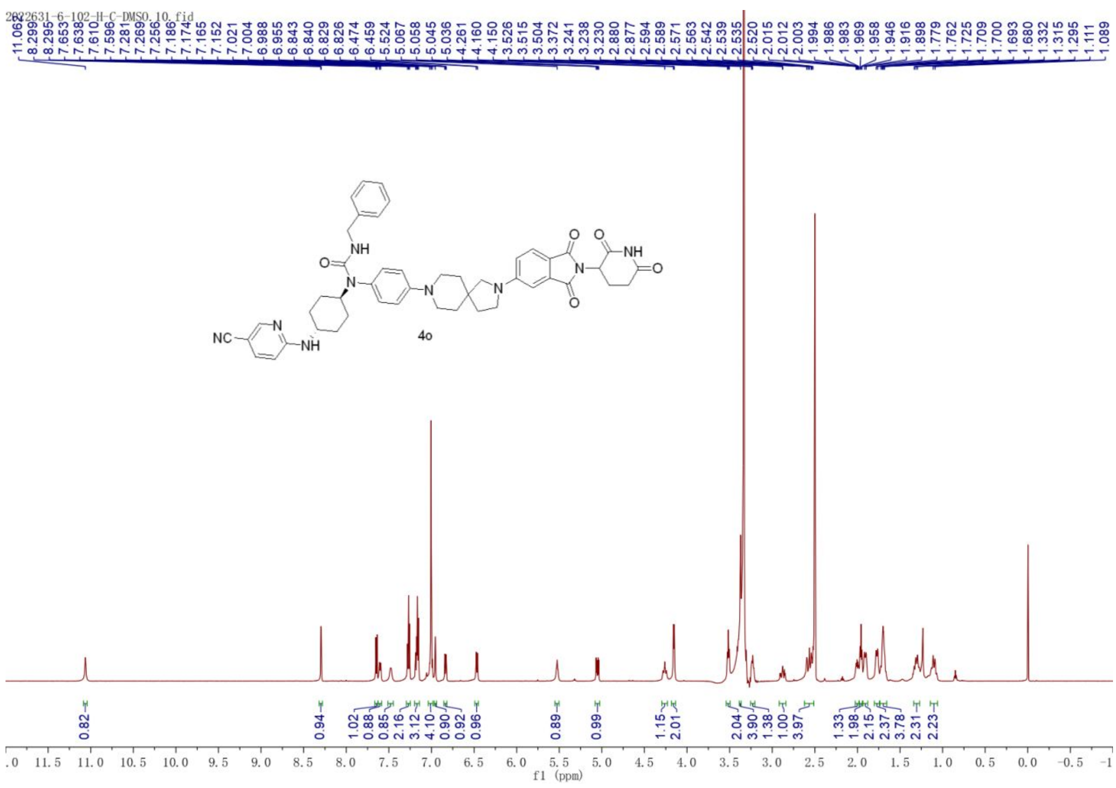
2022631-6-102-H-C-DMSO.10.fid
11.062
8.299
8.295
7.653
7.638
7.610
7.596
7.281
7.269
7.256
7.186
7.174
7.165
7.152
7.021
7.004
6.988
6.955
6.843
6.840
6.829
6.826
6.474
6.459
5.524
5.067
5.058
5.045
5.036
4.261
4.160
4.150
3.526
3.515
3.504
3.372
3.241
3.238
3.230
2.880
2.877
2.594
2.589
2.571
2.563
2.542
2.539
2.535
2.520
2.015
2.012
2.003
1.994
1.986
1.983
1.969
1.958
1.946
1.916
1.898
1.779
1.762
1.725
1.709
1.700
1.693
1.680
1.332
1.315
1.295
1.111
1.089
O
NH
O
N
NH
N
N
N
O
N
O
O
NH
O
NC
N
NH
4o
0.82
0.94
1.02
0.88
0.85
2.16
3.12
4.10
0.90
0.92
0.96
0.89
0.99
1.15
2.01
2.04
3.90
1.38
1.00
3.97
1.33
1.98
2.15
2.37
3.78
2.31
2.23
f1 (ppm)

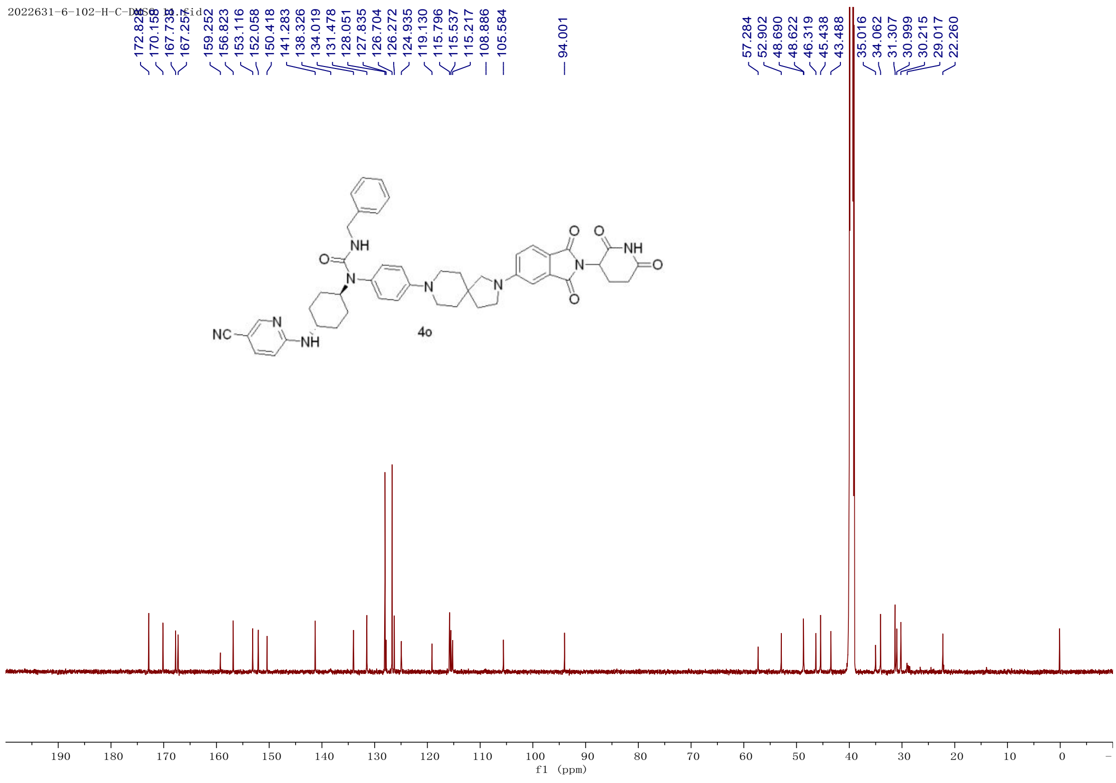
2022631-6-102-H-C-DMSO.11.fid
172.828
170.158
167.738
167.257
159.252
156.823
153.116
152.058
150.418
141.283
138.326
134.019
131.478
128.051
127.835
126.704
126.272
124.935
119.130
115.796
115.537
115.217
108.886
105.584
94.001
57.284
52.902
48.690
48.622
46.319
45.438
43.488
35.016
34.062
31.307
30.999
30.215
29.017
22.260
4o
f1 (ppm)

Mass Spectrum SmartFormula Report

Analysis Info

Analysis Name	D:\Data\SHUJVFENXI\DINGKE-GROUP\2022631-ZLC-6-102_BA4_01_37891.d	Acquisition Date	6/30/2023 12:31:17 PM
Method	20150915.m	Operator	BDAL@DE
Sample Name	2022631-ZLC-6-102	Instrument / Ser#	maXis 4G 21240
Comment			

Acquisition Parameter

Source Type	ESI	Ion Polarity	Positive	Set Nebulizer	1.0 Bar
Focus	Not active	Set Capillary	3000 V	Set Dry Heater	220 °C
Scan Begin	50 m/z	Set End Plate Offset	-500 V	Set Dry Gas	6.0 l/min
Scan End	1500 m/z	Set Collision Cell RF	600.0 Vpp	Set Divert Valve	Waste

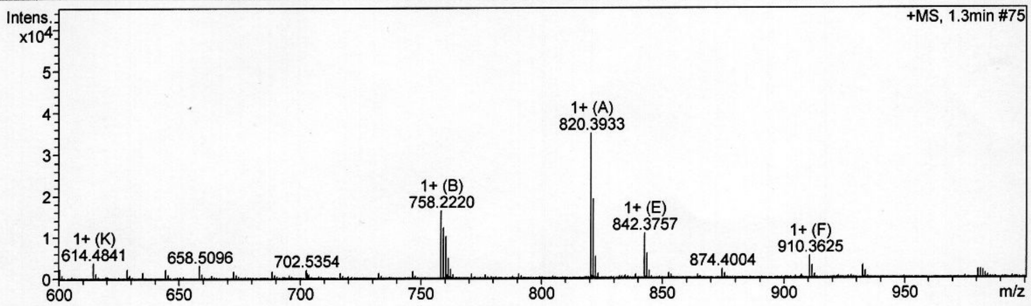

Meas. m/z	#	Formula	Score	m/z	err [ppm]	Mean err [ppm]	mSigma	rdb	e⁻ Conf	N-Rule
820.3933	1	C 47 H 50 N 9 O 5	100.00	820.3929	-0.5	-0.0	2.8	27.5	even	ok
842.3757	1	C 47 H 49 N 9 Na O 5	100.00	842.3749	-1.0	-0.7	8.5	27.5	even	ok

**Data file:**	zlc-6-102.dx		
**Sequence Name:**	zlc-6-101-6-102-6-103	**Project Name:**	1260
**Sample name:**	zlc-6-102	**Operator:**	SYSTEM
**Instrument:**	1260	**Injection date:**	2023-06-28 16:22:29+08:00
**Inj. volume:**	10.000 µL	**Location:**	P1-F3
**Acq. method:**	normal.amx	**Type:**	Sample
**Processing method:**	GC_LC Area Percent_DefaultMethod.pmx	**Sample amount:**	0.00
**Manually modified:**	Manual Integration		

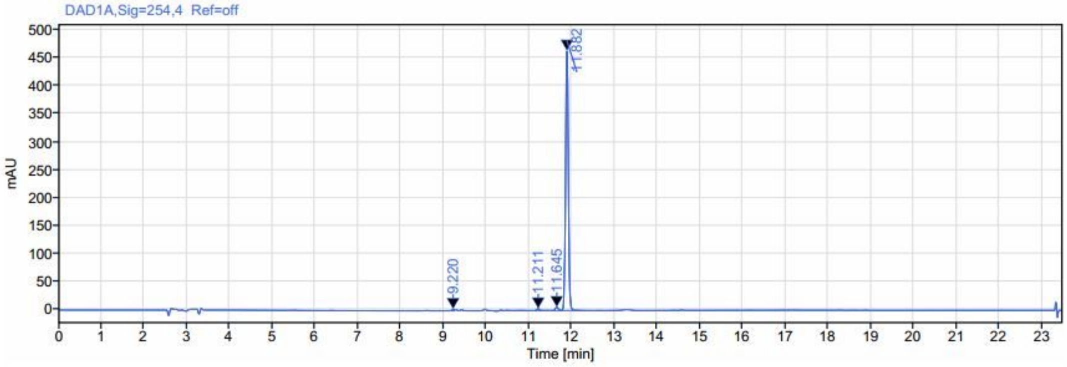

Signal: DAD1A,Sig=254,4 Ref=off

RT [min]	Type	Width [min]	Area	Height	Area%	Name
9.220	BV	0.13	10.78	2.77	0.50	
11.211	BV	0.24	16.71	3.56	0.77	
11.645	VV	0.23	32.02	5.89	1.48	
11.882	VB	0.48	2098.85	463.43	97.24	
		**Sum**	2158.36		100.00	

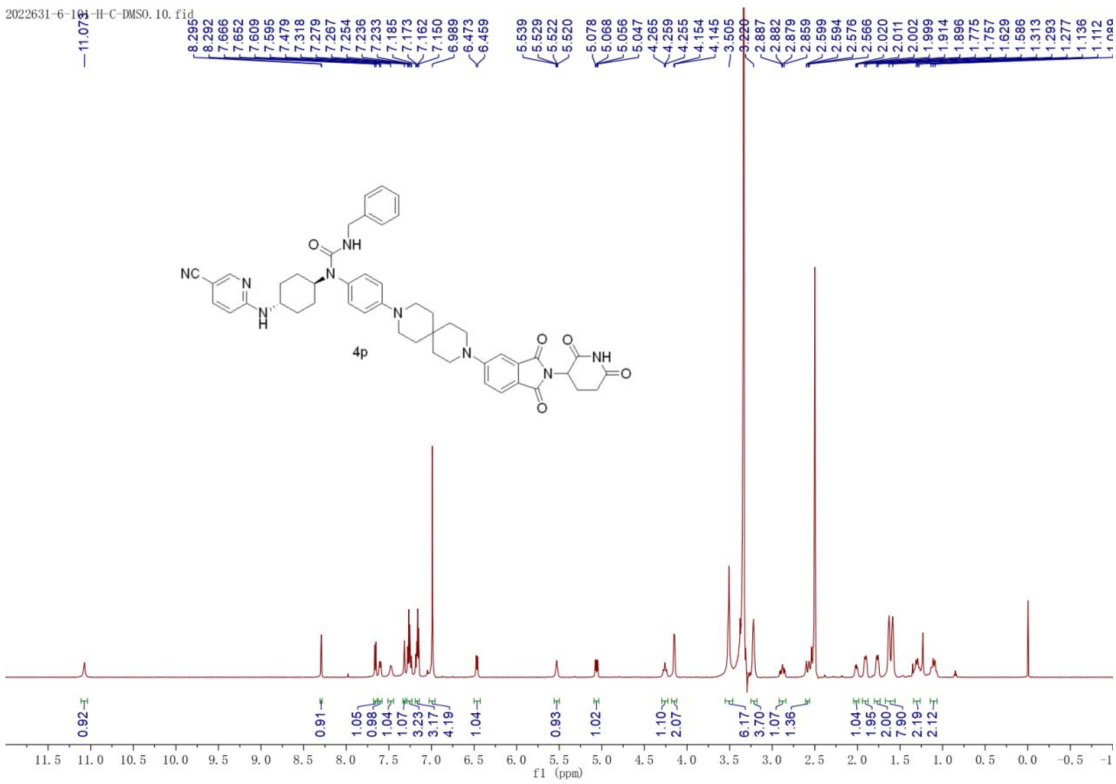
2022631-6-101-H-C-DMSO.10.fid
11.07
8.295 8.292 7.666 7.652 7.609 7.595 7.479 7.318 7.279 7.267 7.254 7.236 7.233 7.185 7.173 7.162 7.150 6.989 6.473 6.459
5.539 5.529 5.522 5.520 5.078 5.068 5.056 5.047 4.265 4.259 4.255 4.154 4.145 3.505 3.220 2.887 2.882 2.879 2.859 2.599 2.594 2.576 2.566 2.020 2.011 2.002 1.999 1.914 1.896 1.775 1.757 1.629 1.586 1.313 1.293 1.277 1.136 1.112
NC
N
N
H
O
NH
N
N
N
O
O
N
O
NH
O
4p
0.92 0.91 1.05 0.98 1.04 1.07 3.23 3.17 4.19 1.04 0.93 1.02 1.10 2.07 6.17 3.70 1.07 1.36 1.04 1.95 2.00 7.90 2.19 2.12
11.5 11.0 10.5 10.0 9.5 9.0 8.5 8.0 7.5 7.0 6.5 6.0 5.5 5.0 4.5 4.0 3.5 3.0 2.5 2.0 1.5 1.0 0.5 0.0 -0.5 -1
f1 (ppm)

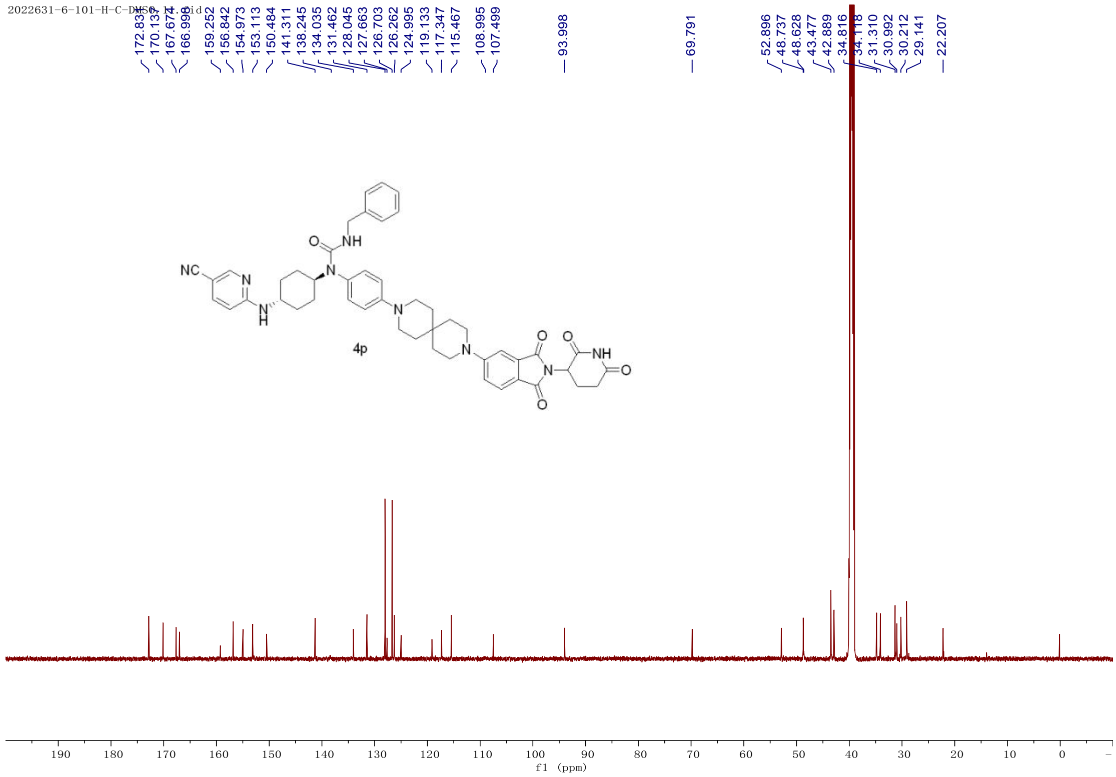
2022631-6-101-H-C-DMSO
172.836 170.137 167.674 166.996 159.252 156.842 154.973 153.113 150.484 141.311 138.245 134.035 131.462 128.045 127.663 126.703 126.262 124.995 119.133 117.347 115.467 108.995 107.499
93.998
69.791
52.896 48.737 48.628 43.477 42.889 34.816 34.118 31.310 30.992 30.212 29.141 22.207
NC
N
N
H
O
NH
N
N
N
O
O
N
O
NH
O
4p
190 180 170 160 150 140 130 120 110 100 90 80 70 60 50 40 30 20 10 0
f1 (ppm)

Mass Spectrum SmartFormula Report

Analysis Info

Analysis Name	D:\Data\SHUJVFENXI\DINGKE-GROUP\2022631-ZLC-6-101_BA5_01_37890.d	Acquisition Date	6/30/2023 12:25:46 PM
Method	20150915.m	Operator	BDAL@DE
Sample Name	2022631-ZLC-6-101	Instrument / Ser#	maXis 4G 21240
Comment			

Acquisition Parameter

Source Type	ESI	Ion Polarity	Positive	Set Nebulizer	1.0 Bar
Focus	Not active	Set Capillary	3000 V	Set Dry Heater	220 °C
Scan Begin	50 m/z	Set End Plate Offset	-500 V	Set Dry Gas	6.0 l/min
Scan End	1500 m/z	Set Collision Cell RF	600.0 Vpp	Set Divert Valve	Waste

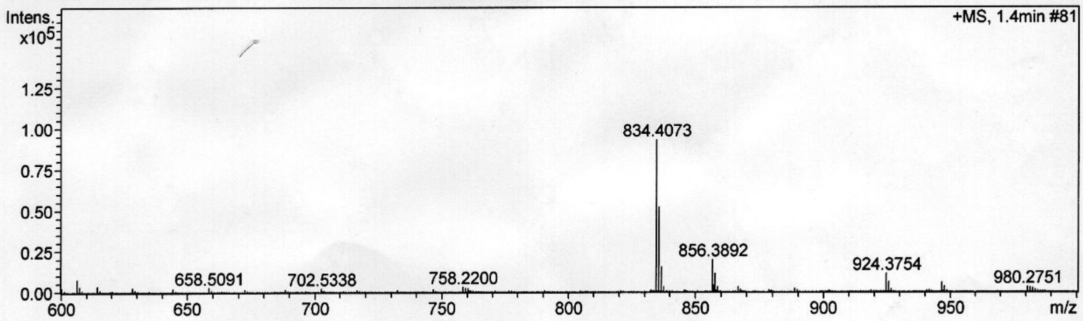

Meas. m/z	#	Formula	Score	m/z	err [ppm]	Mean err [ppm]	mSigma	rdb	e⁻ Conf	N-Rule
834.4073	1	C 48 H 52 N 9 O 5	100.00	834.4086	1.6	1.9	1.6	27.5	even	ok

**Data file:**	zlc-6-101.dx		
**Sequence Name:**	zlc-6-101	**Project Name:**	1260
**Sample name:**	zlc-6-101	**Operator:**	SYSTEM
**Instrument:**	1260	**Injection date:**	2023-06-28 22:26:57+08:00
**Inj. volume:**	10.000 µL	**Location:**	P1-F5
**Acq. method:**	normal.amx	**Type:**	Sample
**Processing method:**	GC_LC Area Percent_DefaultMethod.pmx	**Sample amount:**	0.00
**Manually modified:**	Manual Integration		

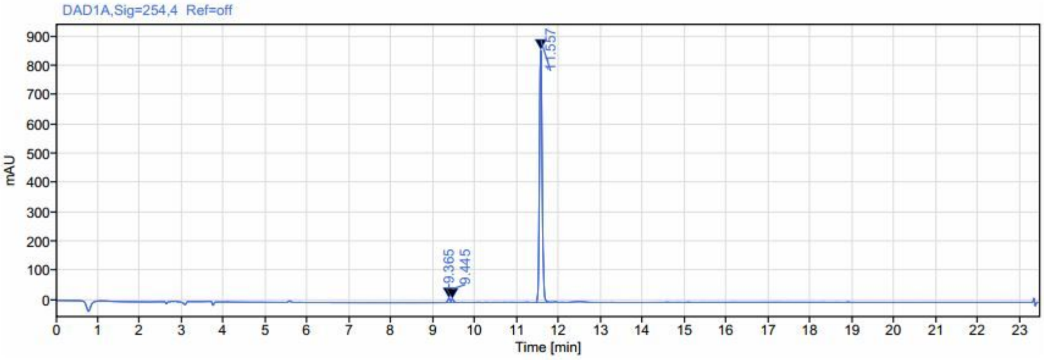

Signal: DAD1A,Sig=254,4 Ref=off

RT [min]	Type	Width [min]	Area	Height	Area%	Name
9.365	BV	0.15	55.55	13.92	1.39	
9.445	VB	0.19	45.69	11.59	1.14	
11.557	BV	0.40	3890.47	861.82	97.46	
		**Sum**	3991.71		100.00	

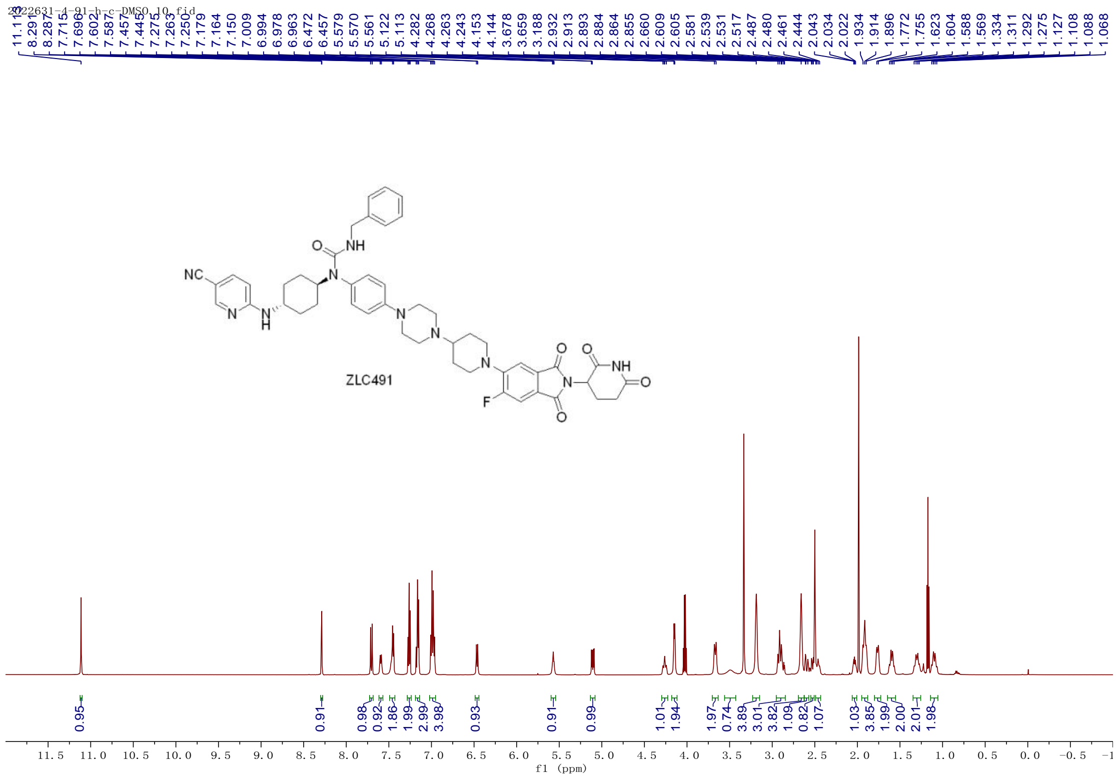
2022631-4-91-h-c-DMSO.10.fid
11.11
8.291
8.287
7.715
7.696
7.602
7.587
7.451
7.445
7.275
7.263
7.250
7.179
7.164
7.150
7.009
6.994
6.978
6.963
6.472
6.457
5.579
5.570
5.561
5.122
5.113
4.282
4.268
4.263
4.243
4.153
4.144
3.678
3.659
3.188
2.932
2.913
2.893
2.884
2.864
2.855
2.660
2.609
2.605
2.581
2.539
2.531
2.517
2.487
2.480
2.461
2.444
2.043
2.034
2.022
1.934
1.914
1.896
1.772
1.755
1.623
1.604
1.588
1.569
1.334
1.311
1.292
1.275
1.127
1.108
1.088
1.068
ZLC491
0.95
0.91
0.98
0.92
1.86
1.99
2.99
3.98
0.93
0.91
0.99
1.01
1.94
1.97
0.74
3.89
3.01
3.82
1.09
0.82
1.07
1.03
3.85
1.99
2.00
2.01
1.98
11.5 11.0 10.5 10.0 9.5 9.0 8.5 8.0 7.5 7.0 6.5 6.0 5.5 5.0 4.5 4.0 3.5 3.0 2.5 2.0 1.5 1.0 0.5 0.0 -0.5 -1
f1 (ppm)

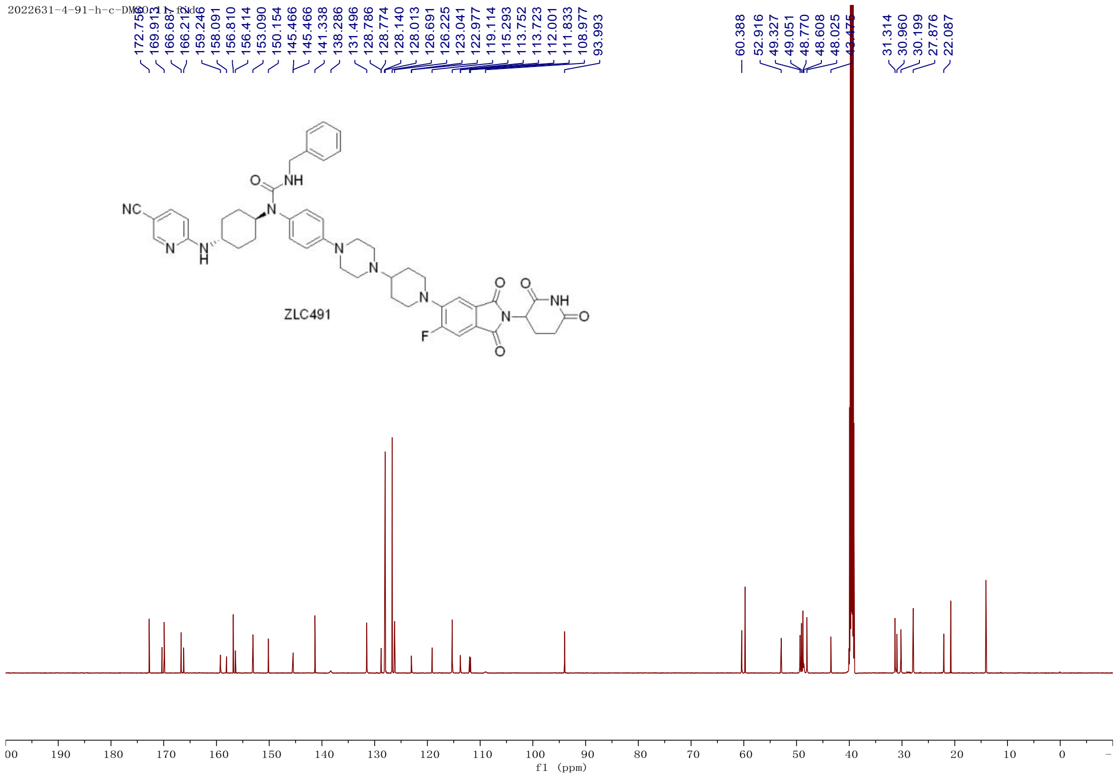
2022631-4-91-h-c-DMSO
172.75
169.913
166.687
166.21
159.246
158.091
156.810
156.414
153.090
150.154
145.466
145.466
141.338
138.286
131.496
128.786
128.774
128.140
128.013
126.691
126.225
123.041
122.977
119.114
115.293
113.752
113.723
112.001
111.833
108.977
93.993
60.388
52.916
49.327
49.051
48.770
48.608
48.025
43.47
31.314
30.960
30.199
27.876
22.087
ZLC491
00 190 180 170 160 150 140 130 120 110 100 90 80 70 60 50 40 30 20 10 0
f1 (ppm)

Mass Spectrum SmartFormula Report

Analysis Info

Analysis Name	D:\Data\SHUJVFENXI\DINGKE-GROUP\2022631-ZLC-4-91_RC7_01_34698.d	Acquisition Date	2/27/2023 5:12:06 PM
Method	20150915.m	Operator	BDAL@DE
Sample Name	2022631-ZLC-4-91	Instrument / Ser#	maXis 4G 21240
Comment			

Acquisition Parameter

Source Type	ESI	Ion Polarity	Positive	Set Nebulizer	1.0 Bar
Focus	Not active	Set Capillary	3000 V	Set Dry Heater	220 °C
Scan Begin	50 m/z	Set End Plate Offset	-500 V	Set Dry Gas	6.0 l/min
Scan End	1500 m/z	Set Collision Cell RF	600.0 Vpp	Set Divert Valve	Waste

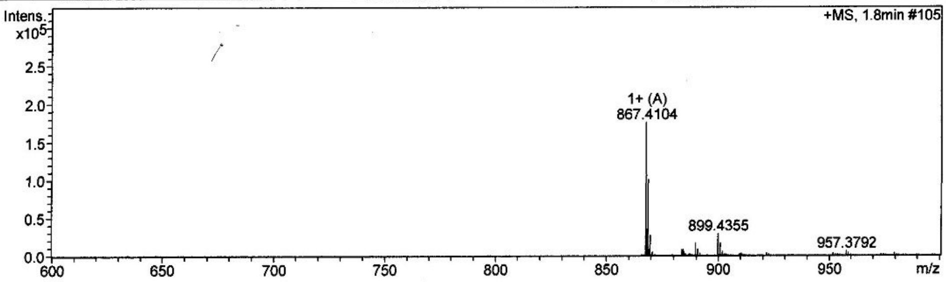

Meas. m/z	#	Formula	Score	m/z	err [ppm]	Mean err [ppm]	mSigma	rdb	e⁻ Conf	N-Rule
867.4104	1	C 48 H 52 F N 10 O 5	100.00	867.4101	-0.4	0.1	7.4	27.5	even	ok

**Data file:**	zlc-4-91-2.dx		
**Sequence Name:**	zlc-4-91-2	**Project Name:**	1260
**Sample name:**	zlc-4-91-2	**Operator:**	SYSTEM
**Instrument:**	1260	**Injection date:**	2023-02-22 16:27:42+08:00
**Inj. volume:**	10.000 µL	**Location:**	P1-B1
**Acq. method:**	normal.amx	**Type:**	Sample
**Processing method:**	GC_LC Area Percent_DefaultMethod.pmx	**Sample amount:**	0.00
**Manually modified:**	Manual Integration		

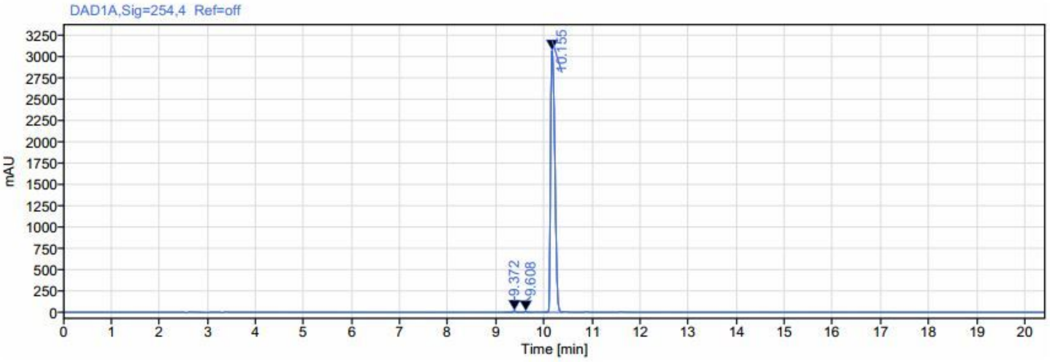

Signal: DAD1A,Sig=254,4 Ref=off

RT [min]	Type	Width [min]	Area	Height	Area%	Name
9.372	BB	0.36	67.02	17.38	0.35	
9.608	BB	0.19	42.34	11.30	0.22	
10.155	VV	0.37	18988.21	3077.38	99.43	
		**Sum**	19097.57		100.00	

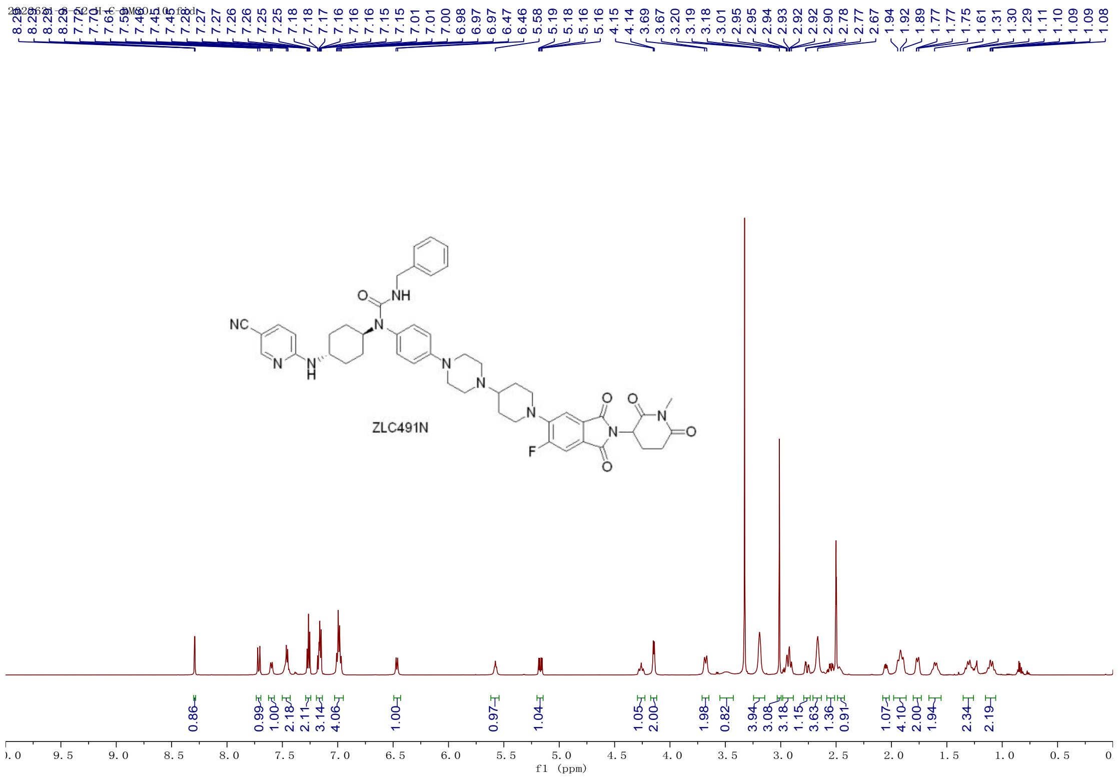
ZLC491N
f1 (ppm)

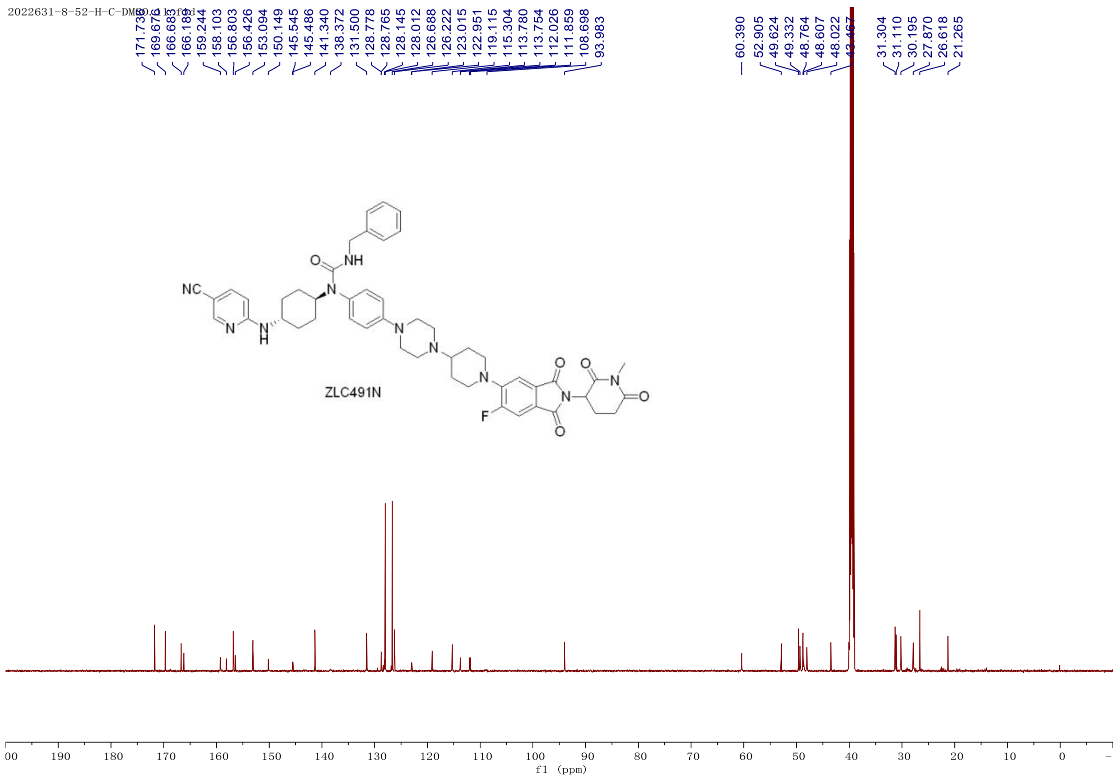
2022631-8-52-H-C-DMSO [illegible]
ZLC491N
f1 (ppm)

Instrument: Thermo Scientific Q Exactive HF Orbitrap-FTMS

Card Serial Number: E233173

Sample Serial Number: zlc-8-52

Operator: Songw Date: 2023/11/16

Operation Mode: ESI Positive Ion Mode

Elemental composition search on mass 881.4257

m/z= 876.4257-886.4257

m/z	Theo. Mass	Delta (ppm)	RDB equiv.	Composition
881.4257	881.4257	-0.07	27.5	$C_{49}H_{54}O_5N_{10}F$
	881.4269	-1.36	23.5	$C_{46}H_{55}O_6N_{10}F_2$

**Data file:**	zlc-8-52-1.dx		
**Sequence Name:**	SingleSample	**Project Name:**	1260
**Sample name:**	zlc-8-52-1	**Operator:**	SYSTEM
**Instrument:**	1260	**Injection date:**	2023-11-09 11:49:56+08:00
**Inj. volume:**	30.000 µL	**Location:**	P1-F6
**Acq. method:**	normal.amx	**Type:**	Sample
**Processing method:**	INM.pmx	**Sample amount:**	0.00
**Manually modified:**	Manual Integration		

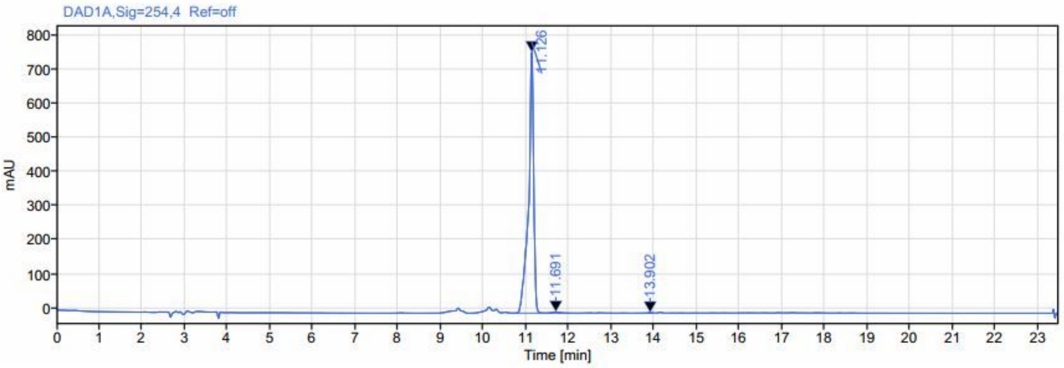

Signal: DAD1A,Sig=254,4 Ref=off

RT [min]	Type	Width [min]	Area	Height	Area%	Name
11.126	BV	0.76	6489.75	765.00	99.14	
11.691	VB	0.59	43.10	3.57	0.66	
13.902	BV	0.47	12.97	1.87	0.20	
		**Sum**	6545.82		100.00	

The ^{19}F NMR of ZLC491

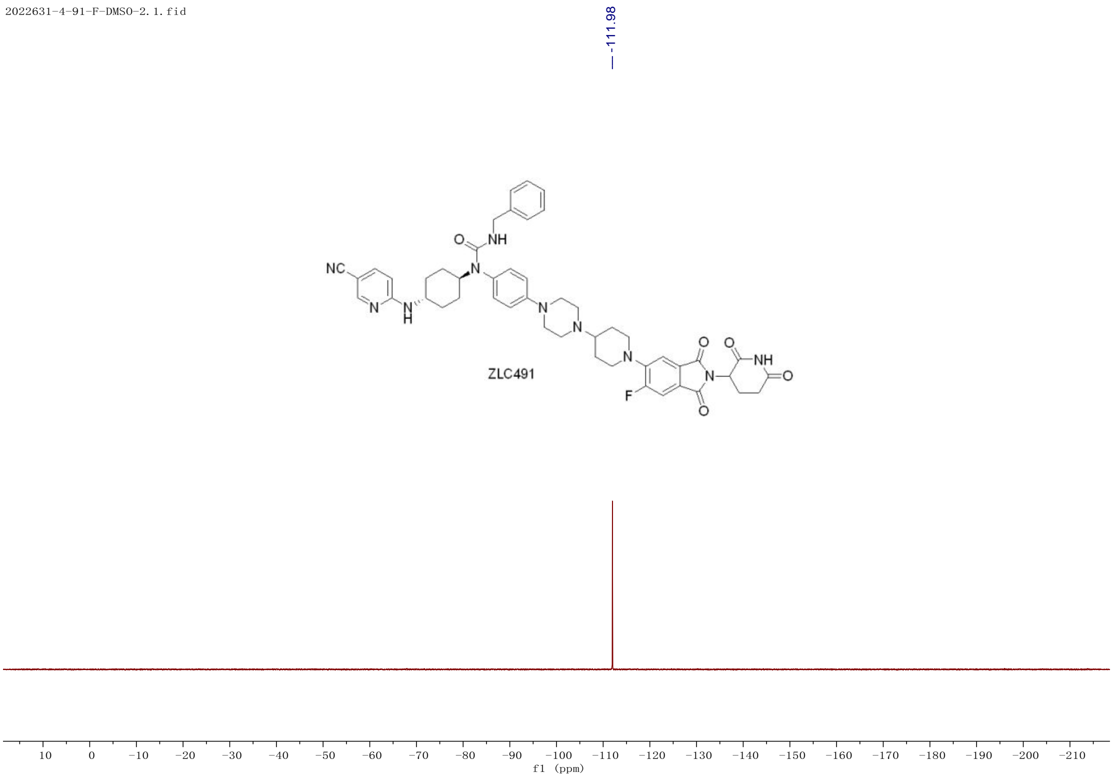

Supplement: Supplementary file 1 — jm4c01596_si_001.pdf [file jm4c01596_si_001.pdf]
